# Supplementary material for: Falling short: Inadequate target attainment in guideline-based therapy of nontuberculous mycobacterial disease
Source: medRxiv. 2026 Sep 27:2026.09.24.26363927. Preprint. [Version 1] doi: 10.64898/2026.09.24.26363927 (PMC13622600; doi:10.64898/2026.09.24.26363927)
Supplement: Supplementary file 1 [file media-3.pdf]

Supplementary Dataset 2

Contents

A Comparison of literature versus reimplemented AUC values 7

B Introduction 8

C Amikacin 9
- C.1 Amikacin model parameters 9
- C.2 Amikacin model equations 9
- C.3 Amikacin model flowchart 9
- C.4 Amikacin model AUC distributions 10
- C.5 Amikacin model concentration time-curves 11

D Bedaquiline 13
- D.1 Bedaquiline model parameters 13
- D.2 Bedaquiline model equations 13
- D.3 BDQ model flowchart 14
- D.4 Bedaquiline model AUC distributions 15
- D.5 Bedaquiline model concentration time-courses 16

E Clofazimine 18
- E.1 Clofazimine model parameters 18
- E.2 Clofazimine model equations 18
- E.3 Clofazimine model flowchart 19
- E.4 Clofazimine model AUC distributions 19
- E.5 Clofazimine model concentration time-courses 20

F Clarithromycin 22
- F.1 Clarithromycin model parameters 22
- F.2 Clarithromycin model equations 22
- F.3 Clarithromycin model flowchart 23
- F.4 Clarithromycin model AUC distributions 23
- F.5 Clarithromycin model concentration time-courses 24

G Ethambutol 28
- G.1 Ethambutol model parameters 28
- G.2 Ethambutol model equations 28
- G.3 Ethambutol model flowchart 29
- G.4 Ethambutol model AUC distributions 30
- G.5 Ethambutol model concentration time-courses 31

H Cefoxitin 33
- H.1 Cefoxitin model parameters 33
- H.2 Cefoxitin model equations 33
- H.3 Cefoxitin model flowchart 34
- H.4 Cefoxitin model AUC distributions 35
- H.5 Cefoxitin model concentration time-courses 36

I Imipenem 38
- I.1 Imipenem model parameters 38
- I.2 Imipenem model equations 38
- I.3 Imipenem model flowchart 38
- I.4 Imipenem model AUC distributions 39

I.5 Imipenem model concentration time-courses . . . 40

J Linezolid **42**
J.1 Linezolid model parameters . . . 42
J.2 Linezolid model equations . . . 42
J.3 Linezolid model flowchart . . . 43
J.4 Linezolid model AUC distributions . . . 44
J.5 Linezolid model concentration time-courses . . . 45

K Moxifloxacin **47**
K.1 Moxifloxacin model parameters . . . 47
K.2 Moxifloxacin model equations . . . 47
K.3 Moxifloxacin model flowchart . . . 47
K.4 Moxifloxacin model AUC distributions . . . 48
K.5 Moxifloxacin model concentration time-courses . . . 49

L Rifabutin **51**
L.1 Rifabutin model parameters . . . 51
L.2 Rifabutin model equations . . . 51
L.3 Rifabutin model flowchart . . . 52
L.4 Rifabutin model AUC distributions . . . 53
L.5 Rifabutin model concentration time-courses . . . 54

M Rifampin **56**
M.1 Rifampin model parameters . . . 56
M.2 Rifampin model equations . . . 56
M.3 Rifampin model flowchart . . . 57
M.4 Rifampin model AUC distributions . . . 58
M.5 Rifampin model concentration time-courses . . . 59

N Tigecycline **61**
N.1 Tigecycline model parameters . . . 61
N.2 Tigecycline model equations . . . 61
N.3 Tigecycline model flowchart . . . 61
N.4 Tigecycline model AUC distributions . . . 62
N.5 Tigecycline model concentration time-courses . . . 63

O Omadacycline **65**
O.1 Omadacycline model parameters . . . 65
O.2 Omadacycline model equations . . . 65
O.3 Omadacycline model flowchart . . . 66
O.4 Omadacycline model AUC distributions . . . 67
O.5 Omadacycline model concentration time-courses . . . 68

List of Figures

S2 Schematic of the two-compartment population pharmacokinetic model for amikacin, showing drug transfer between the central (C_1) and peripheral (C_2) compartments, systemic clearance (CL), and intercompartmental transfer (Q). 9

S3 Steady-state $AUC_{0–24}$ distributions across 10,000 simulated patient profiles for amikacin at 15 mg/kg q24h. Distributions reflect interindividual variability drawn from the parameter distributions in Table S2. 10

S4 Simulated steady-state central compartment concentration-time profiles for amikacin across 10,000 virtual patients receiving 15 mg/kg q24h. 11

S5 Simulated steady-state central compartment concentration-time profiles for amikacin showing the mean and 90% prediction interval across 10,000 virtual patients receiving 15 mg/kg q24h. 12

S6 Schematic of the ten-compartment population pharmacokinetic model for bedaquiline, showing the absorption compartment (q_0), five transit compartments (q_1–q_5), final transit compartment (q), central compartment (C), and two peripheral compartments (C_2) and (C_3), with systemic clearance (CL) and intercompartmental transfer (Q_2, Q_3). 14

S7 Steady-state $AUC_{0–24}$ distributions across 10,000 simulated patient profiles for bedaquiline at 400 mg q24h for two weeks followed by 200 mg three times weekly. Distributions reflect interindividual variability drawn from the parameter distributions in Table S3. 15

S8 Simulated steady-state central compartment concentration-time profiles for bedaquiline across 10,000 virtual patients during the maintenance phase of 200 mg three times weekly (following 400 mg q24h for two weeks). 16

S9 Simulated steady-state central compartment concentration-time profiles for bedaquiline showing the mean and 90% prediction interval across 10,000 virtual patients during the maintenance phase of 200 mg three times weekly (following 400 mg q24h for two weeks). 17

S10 Schematic of the three-compartment population pharmacokinetic model for clofazimine, showing the absorption compartment (q_0), central compartment (C), and two peripheral compartments (C_{p1}) and (C_{p2}), with systemic clearance (CL/F) and intercompartmental transfer (Q1/F, Q2/F). 19

S11 Steady-state $AUC_{0–24}$ distributions across 10,000 simulated patient profiles for clofazimine at 100 mg q24h. Distributions reflect interindividual variability drawn from the parameter distributions in Table S4. 19

S12 Simulated steady-state central compartment concentration-time profiles for clofazimine across 10,000 virtual patients receiving 100 mg q24h. 20

S13 Simulated steady-state central compartment concentration-time profiles for clofazimine showing the mean and 90% prediction interval across 10,000 virtual patients receiving 100 mg q24h. 21

S14 Schematic of the three-compartment population pharmacokinetic model for clarithromycin, showing the absorption compartment (q_0), central compartment (C), peripheral compartment (C_2), and epithelial compartment (C_3), with systemic clearance (CL/F) and intercompartmental transfer rate constants (K_12, K_21, K_13, K_31). 23

S15 Steady-state $AUC_{0–24}$ distributions across 10,000 simulated patient profiles for clarithromycin at 500 mg q12h. Distributions reflect interindividual variability drawn from the parameter distributions in Table S5. 23

S16 Simulated steady-state central compartment concentration-time profiles for clarithromycin across 10,000 virtual patients receiving 500 mg q12h. 24

S17 Simulated steady-state epithelial lining fluid concentration-time profiles for clarithromycin across 10,000 virtual patients receiving 500 mg q12h. 25

S18 Simulated steady-state central compartment concentration-time profiles for clarithromycin showing the mean and 90% prediction interval across 10,000 virtual patients receiving 500 mg q12h. 26

S19 Simulated steady-state epithelial lining fluid concentration-time profiles for clarithromycin showing the mean and 90% prediction interval across 10,000 virtual patients receiving 500 mg q12h. 27

S20 Schematic of the two-compartment population pharmacokinetic model for ethambutol with transit-compartment absorption, showing the absorption input (q), central compartment (C), and peripheral compartment (C_p), with systemic clearance (CL_F), intercompartmental clearance (Q_F), and HIV covariate effect on bioavailability (F). 29
S21 Steady-state $\text{AUC}_{0\text{–}24}$ distributions across 10,000 simulated patient profiles for ethambutol at 15 mg/kg q24h. Distributions reflect interindividual variability drawn from the parameter distributions in Table S6. 30
S22 Simulated steady-state central compartment concentration-time profiles for ethambutol across 10,000 virtual patients at 15 mg/kg q24h. 31
S23 Simulated steady-state central compartment concentration-time profiles for ethambutol showing the mean and 90% prediction interval across 10,000 virtual patients at 15 mg/kg q24h. . . 32
S24 Schematic of the one-compartment population pharmacokinetic model for cefoxitin, showing the central compartment (C) with systemic clearance (CL) and a short IV infusion input. Drug is eliminated from the central compartment by a first-order process. 34
S25 Steady-state $\text{AUC}_{0\text{–}24}$ distribution across the simulated virtual-patient population for cefoxitin at 4 g q12h administered as a 3-hour IV infusion. The distribution reflects interindividual variability in V_c and CL drawn from the parameters in Table S7. 35
S26 Simulated steady-state central-compartment concentration-time profiles for cefoxitin across the virtual-patient population receiving 4 g q12h as a 3-hour IV infusion. 36
S27 Simulated steady-state central-compartment concentration-time profiles for cefoxitin showing the median (50th percentile) and the 5th–95th percentile (90%) prediction interval across the virtual-patient population receiving 4 g q12h as a 3-hour IV infusion. 37
S28 Schematic of the two-compartment population pharmacokinetic model for imipenem, showing the central compartment (C) and peripheral compartment (C_p), with systemic clearance (CL_F), intercompartmental clearance (Q_F), and 3-hour IV infusion input. 38
S29 Steady-state $\text{AUC}_{0\text{–}24}$ distributions across 10,000 simulated patient profiles for imipenem at 1000 mg q12h administered as a 3-hour IV infusion. Distributions reflect interindividual variability drawn from the parameter distributions in Table S8. 39
S30 Simulated steady-state central compartment concentration-time profiles for imipenem across 10,000 virtual patients receiving 1000 mg q12h as a 3-hour IV infusion. 40
S31 Simulated steady-state central compartment concentration-time profiles for imipenem showing the mean and 90% prediction interval across 10,000 virtual patients receiving 1000 mg q12h as a 3-hour IV infusion. 41
S32 Schematic of the eight-compartment population pharmacokinetic model for linezolid, showing the absorption compartment (q_0), five transit compartments (q_1–q_5), final pre-absorption compartment (q), and central compartment (C), with absorption rate constant (K_a), transit rate constant (k_tr), bioavailability (F), and systemic clearance (CL). 43
S33 Steady-state $\text{AUC}_{0\text{–}24}$ distributions across 10,000 simulated patient profiles for linezolid at 600 mg q24h. Distributions reflect interindividual variability drawn from the parameter distributions in Table S9. 44
S34 Simulated steady-state central compartment concentration-time profiles for linezolid across 10,000 virtual patients receiving 600 mg q24h. 45
S35 Simulated steady-state central compartment concentration-time profiles for linezolid showing the mean and 90% prediction interval across 10,000 virtual patients receiving 600 mg q24h. . 46
S36 Schematic of the one-compartment population pharmacokinetic model for moxifloxacin, showing the absorption compartment (C_q) and central compartment (C), with first-order absorption rate constant (ka) and systemic clearance (CL_F). 47
S37 Steady-state $\text{AUC}_{0\text{–}24}$ distributions across 10,000 simulated patient profiles for moxifloxacin at 400 mg q24h and 800 mg q24h. Distributions reflect interindividual variability drawn from the parameter distributions in Table S10. 48
S38 Simulated steady-state central compartment concentration-time profiles for moxifloxacin across 10,000 virtual patients receiving 400 mg q24h and 800 mg q24h. 49

S39 Simulated steady-state central compartment concentration-time profiles for moxifloxacin showing the mean and 90% prediction interval across 10,000 virtual patients receiving 400 mg q24h and 800 mg q24h. 50
S40 Schematic of the population pharmacokinetic model for rifabutin, showing the transit-compartment absorption input (m_{abs}), rifabutin central compartment ($C_{C,RFB}$), rifabutin peripheral compartment ($C_{P,RFB}$), des-rifabutin central compartment ($C_{C,dRFB}$), and des-rifabutin peripheral compartment ($C_{P,dRFB}$), with first-pass metabolism fraction (F_m), absorption rate constant (k_a), systemic clearances (CL_1, CL_2), intercompartmental clearances (Q_1, Q_2), and central compartment conversion clearance (Q_3). 52
S41 Steady-state $AUC_{0–24}$ distributions across 10,000 simulated patient profiles for rifabutin at 300 mg q24h. Distributions reflect interindividual variability drawn from the parameter distributions in Table S11. 53
S42 Simulated steady-state central compartment concentration-time profiles for rifabutin across 10,000 virtual patients receiving 300 mg q24h. 54
S43 Simulated steady-state central compartment concentration-time profiles for rifabutin showing the mean and 90% prediction interval across 10,000 virtual patients receiving 300 mg q24h. . 55
S44 Schematic of the one-compartment population pharmacokinetic model for rifampin with transit-compartment absorption, showing the absorption compartment (q_0) and central compartment (C), with absorption rate constant (k_a), transit rate constant (k_tr), bioavailability (F), and systemic clearance (CL/F). 57
S45 Steady-state $AUC_{0–24}$ distributions across 10,000 simulated patient profiles for rifampin at 600 mg q24h. Distributions reflect interindividual variability drawn from the parameter distributions in Table S12. 58
S46 Simulated steady-state central compartment concentration-time profiles for rifampin across 10,000 virtual patients receiving 600 mg q24h. 59
S47 Simulated steady-state central compartment concentration-time profiles for rifampin showing the mean and 90% prediction interval across 10,000 virtual patients receiving 600 mg q24h. . 60
S48 Schematic of the two-compartment population pharmacokinetic model for tigecycline, showing the central compartment (C) and peripheral compartment (C_p), with systemic clearance (CL_F), intercompartmental clearance (Q_F), and 3-hour IV infusion input with loading dose. . 61
S49 Steady-state $AUC_{0–24}$ distributions across 10,000 simulated patient profiles for tigecycline at 25 mg q12h administered as a 3-hour IV infusion. Distributions reflect interindividual variability drawn from the parameter distributions in Table S13. 62
S50 Simulated steady-state central compartment concentration-time profiles for tigecycline across 10,000 virtual patients receiving a loading dose followed by 25 mg q12h as a 3-hour IV infusion. 63
S51 Simulated steady-state central compartment concentration-time profiles for tigecycline showing the mean and 90% prediction interval across 10,000 virtual patients receiving a loading dose followed by 25 mg q12h as a 3-hour IV infusion. 64
S52 Schematic of the four-compartment population pharmacokinetic model for omadacycline, showing two parallel absorption compartments (q_1, q_2), central compartment (C), and peripheral compartment (P), with absorption rate constants (k_a,1, k_a,2), lag time (T_lag), systemic clearance (CL_F,1), and intercompartmental clearance (CL_F,2). 66
S53 Steady-state $AUC_{0–24}$ distributions across 10,000 simulated patient profiles for omadacycline at 300 mg q24h. Distributions reflect interindividual variability drawn from the parameter distributions in Table S14. 67
S54 Simulated steady-state central compartment concentration-time profiles for omadacycline across 10,000 virtual patients receiving 300 mg q24h. 68
S55 Simulated steady-state central compartment concentration-time profiles for omadacycline showing the mean and 90% prediction interval across 10,000 virtual patients receiving 300 mg q24h. 69

List of Tables

S1 Pharmacokinetic model summary across antibiotics: model structure, dosing used in the present work, typical literature AUC range, mean AUC and C_{max} from the reimplemented model, and supporting references. . . . 7
S2 Population pharmacokinetic parameters for the two-compartment amikacin model. Values represent typical population means; IIV is expressed as percent coefficient of variation ($\%CV$). 9
S3 Population pharmacokinetic parameters for the ten-compartment bedaquiline model. Values represent typical population means; IIV is expressed as percent coefficient of variation ($\%CV$). 13
S4 Population pharmacokinetic parameters for the three-compartment clofazimine model. Values represent typical population means; IIV and IOV are expressed as percent coefficient of variation ($\%CV$). . . . 18
S5 Population pharmacokinetic parameters for the three-compartment clarithromycin model. Values represent typical population means; IIV is expressed as percent coefficient of variation ($\%CV$). . . . 22
S6 Population pharmacokinetic parameters for the transit-compartment ethambutol model. Values represent typical population means; IIV and IOV are expressed as percent coefficient of variation ($\%CV$). . . . 28
S7 Population pharmacokinetic parameters for the one-compartment cefoxitin model. Values are the typical population means reported in the original source [32]; interindividual variability (IIV) is expressed as percent coefficient of variation ($\%CV$). . . . 33
S8 Population pharmacokinetic parameters for the two-compartment imipenem model. Values represent typical population means; IIV is expressed as percent coefficient of variation ($\%CV$). 38
S9 Population pharmacokinetic parameters for the transit-compartment linezolid model. Values represent typical population means; IIV and IOV are expressed as percent coefficient of variation ($\%CV$). . . . 42
S10 Population pharmacokinetic parameters for the one-compartment moxifloxacin model. Values represent typical population means; IIV is expressed as percent coefficient of variation ($\%CV$). 47
S11 Population pharmacokinetic parameters for the transit-compartment rifabutin model with parallel des-rifabutin metabolite tracking. Values represent typical population means; IIV is expressed as percent coefficient of variation ($\%CV$). . . . 51
S12 Population pharmacokinetic parameters for the transit-compartment rifampin model. Values represent typical population means; θ denotes the dose-type effect coefficient, η denotes the interindividual variability variance, and κ denotes the interoccasional variability variance. . . 56
S13 Population pharmacokinetic parameters for the two-compartment tigecycline model. Values represent typical population means; IIV is expressed as percent coefficient of variation ($\%CV$). 61
S14 Population pharmacokinetic parameters for the two-compartment omadacycline model with parallel absorption. Values represent typical population means; IIV is expressed as percent coefficient of variation ($\%CV$). . . . 65

A Comparison of literature versus reimplemented AUC values

Table S1: Pharmacokinetic model summary across antibiotics: model structure, dosing used in the present work, typical literature AUC range, mean AUC and C_{max} from the reimplemented model, and supporting references.

Antibiotic	# Comp.	Variability	Dose	Typical AUC range (mg·h/L)	Reimplemented Means ± s.d.		References & comments
					AUC (mg·h/L)	C_{max} (mg/L)	
Amikacin (AMK) [44]	2	IIV	15 mg/kg 1h infusion QD	AUC: ∼150; C_{max}: 35–50 mg/L at end of 30–60 min infusion	219.49 ± 46.39	59.00 ± 6.78	AUC: 154.53 ± 29.91 mg·h/L in 6 HV [15]; 152.82 ± 50.83 mg·h/L [21]. C_{max}: 35–50 mg/L [39]; 35–45 mg/L median [23]; 55.30–16.88 mg/L [21].
Bedaquiline (BDQ) [27]	10	IIV	400/200 mg	∼20–24 at steady state	18.99 ± 4.97	1.39 ± 0.38	21.648 mg·h/L at wk 8 [13]; ∼25 mg·h/L [29].
Cefoxitin (FOX) [32]	3	IIV	4 g BID	≈448 for a 4 g 5-min infusion BID; ≈500 for a 4 g 8-h infusion BID	830.10 ± 460.47	92.73 ± 34.68	Concentration–time profiles replotted and AUCs estimated from [11] and [25], assuming dose proportionality.
Clarithromycin (CLR) [20]	3	IIV	500 mg BID	36 (range 17–67)	29.27 ± 3.92	1.70 ± 0.22	15–20 mg·h/L for ONE 500 mg dose [36]; 36 mg·h/L total AUC for TWO 500 mg doses [4].
Clofazimine (CFZ) [1]	4	IIV, IOV	100 mg QD	4–16	6.93 ± 1.38	0.34 ± 0.07	Weekly AUC reported as 60 mg·h/L → ∼8.5 daily; C_{ave} of 0.36 μg/mL [2] → AUC ≈ 8.64 mg·h/L; 15.97 mg·h/L (IQR 11.7–21.7) [18], but the same paper shows daily AUC = 7.3 mg·h/L at 200 mg, implying ∼4 mg·h/L at 100 mg.
Ethambutol (EMB) [24]	3	IIV, IOV	15 mg/kg QD	13–23 at 15 mg/kg	22.15 ± 6.15	2.76 ± 0.82	∼25–29 mg·h/L at 25 mg/kg [34]; 23.6 mg·h/L (IQR 20.5–28.9) at 15–25 mg/kg [12]; 13 mg·h/L at 15 mg/kg [19].
Imipenem (IPM) [9]	2	IIV	1000 mg 3h infusion BID	120–288	229.05 ± 40.94	25.97 ± 3.05	63.9 ± 9.1 mg·h/L after ONE 1-g infusion → ∼128 mg·h/L BID [40]; 109.8 mg·h/L (103.2–116.8) after ONE 1-g infusion → ∼220 mg·h/L BID [45]; 144.22 ± 73.63 mg·h/L after ONE 2-h 1-g infusion → ∼288 mg·h/L BID [22].
Linezolid (LZD) [3]	2	IIV, IOV	600 mg QD	80–130	183.92 ± 77.57	14.42 ± 4.06	84.6 mg·h/L (64.7–125.0) [28]; 138.1 ± 27.6 on D1 and 234 ± 55.2 on D7 at 600 mg BID [8]; 80–128 mg·h/L [42]; AUC_{0-12} = 96.73 ± 56.45 mg·h/L [14].
Moxifloxacin (MXF) [38]	2	IIV	400 mg QD	20–55 at 400 mg QD	43.37 ± 13.41	3.96 ± 1.09	27–48 mg·h/L [31]; 55 (36–79) mg·h/L [35]; ∼40 mg·h/L [49]; 32.78 mg·h/L (IQR 22.75–47.31) [33]; 19–30 mg·h/L [10].
Rifampicin (RIF) [46]	2	IIV	600 mg QD	15–60	33.19 ± 19.37	6.34 ± 2.62	Meta-analysis summary estimate of 38.73 mg·h/L at steady state across 35 studies [43].
Rifabutin (RBT) [16]	5	IIV	300 mg QD	3–5	4.50 ± 2.17	0.45 ± 0.20	2.71 (1.39–3.98) mg·h/L at wk 2–4; 2.97 (0.60–4.67) mg·h/L at wk 4–5; 4.36 (1.73–6.09) mg·h/L at wk 6–7 [17]; 4.08 mg·h/L [7]; 5.4 mg·h/L [41].
Tigecycline (TIG) [6]	2	IIV	25 mg 1h infusion BID	1.6–3.5	3.68 ± 1.68	0.37 ± 0.10	2.3 mg·h/L [30]; 1.6 mg·h/L (NDA document); AUC_{0-12} = 1.784 ± 0.173 mg·h/L at 25 mg → AUC_{0-24} ≈ 3.5 mg·h/L [47].
Omadacycline (OMC) [48]	4	IIV	300 mg QD	9–20	20.13 ± 4.24	1.63 ± 0.30	19.4–20.4 mg·h/L [48]; 11.156 mg·h/L [26]; 10.19 (8.35–11.01) [37]; 8.5–13 mg·h/L ([5], Table S5).

B Introduction

All of the following antibiotic models are available for download at github.itap.purdue.edu/ElsjePienaarGroup. Several equations recur across the drug-specific models and are collected here; subsequent sections reference them as needed rather than restating them.

Infusion rate

The infusion rate used by all intravenously administered drugs is given by:

$$\texttt{infusion rate} = R(t) = \begin{cases} \frac{\texttt{dose}}{\tau_{\text{inf}}} & 0 \le t < \tau_{\text{inf}} \\ 0 & t \ge \tau_{\text{inf}} \end{cases} \tag{1}$$

where $R(t)$ is the infusion rate as a function of time, $\texttt{dose}$ is the administered dose amount (mg) for the dosing period, τ_{inf} is the infusion duration (h), and t is the elapsed time within the current dosing period.

Oral absorption

The gamma constant used in the oral absorption compartment is given by:

$$\Gamma_{\texttt{n}} = \sqrt{2\pi} \cdot \texttt{n}^{\texttt{n}+0.5} \cdot \exp(-\texttt{n}) \tag{2}$$

where Γ_n is the constant used in the oral absorption compartment and $\texttt{n}$ is the number of transport compartments.

Interindividual and interobservational variability

Interindividual and interobservational variability are introduced by:

$$\theta_i = \theta \cdot \exp \eta_i \tag{3}$$

where θ_i is the parameter value for the i-th subject, θ is the mean value, and η_i is a random interindividual or interobservational variable, normally distributed with a mean of zero and variance ω^2.

The coefficient of variation is related to the log-domain variance by:

$$\text{CV} = \sqrt{\exp(\omega^2) - 1} \tag{4}$$

Equation 4 is used to convert between the coefficient of variation of the parameter values and the variance in the log-domain (ω^2), depending on which value is reported in the source paper.

The infusion rate (Equation 1) is used by all intravenous (IV) drugs (AMK, FOX, IMI, TIG). The gamma (Γ) term (Equation 2) is used by EMB and RIF. The CV relationship (Equation 4) is used by every model carrying an interindividual variability (IIV) or interobservational variability (IOV) to determine the patient-specific coefficient values for the Monte Carlo simulations.

C Amikacin

C.1 Amikacin model parameters

Table S2: Population pharmacokinetic parameters for the two-compartment amikacin model. Values represent typical population means; IIV is expressed as percent coefficient of variation ($\%CV$).

Parameter	Mean	IIV % CV	Units
`Vc`	8.92	15	L
`Vp`	11.4	25	L
`CL`	3.6	21	L/h
`Q`	4.43	30	L/h

`Vc` represents volume of distribution for the central compartment; `Vp` represents the volume of distribution for the peripheral compartment; `CL` represents the system clearance; `Q` represents the transfer rate between the central compartment and the peripheral compartment.

C.2 Amikacin model equations

This model was recreated from the model proposed in [44]. The parameter values used in the patient simulations were calculated using Equation 4. The model structure was a two compartment model as a central compartment (C_1) and a peripheral compartment (C_2) as shown in Figure S2.

$$\frac{d(C_1)}{dt} = \texttt{infusion rate} + \frac{1}{\texttt{Vc}}\Big[(\texttt{Q}\cdot C_1) - (\texttt{Q}\cdot C_2) - (\texttt{CL}\cdot C_1)\Big] \tag{5}$$

$$\frac{d(C_2)}{dt} = \frac{1}{\texttt{Vp}}\Big[(\texttt{Q}\cdot C_1) - (\texttt{Q}\cdot C_2)\Big] \tag{6}$$

C.3 Amikacin model flowchart

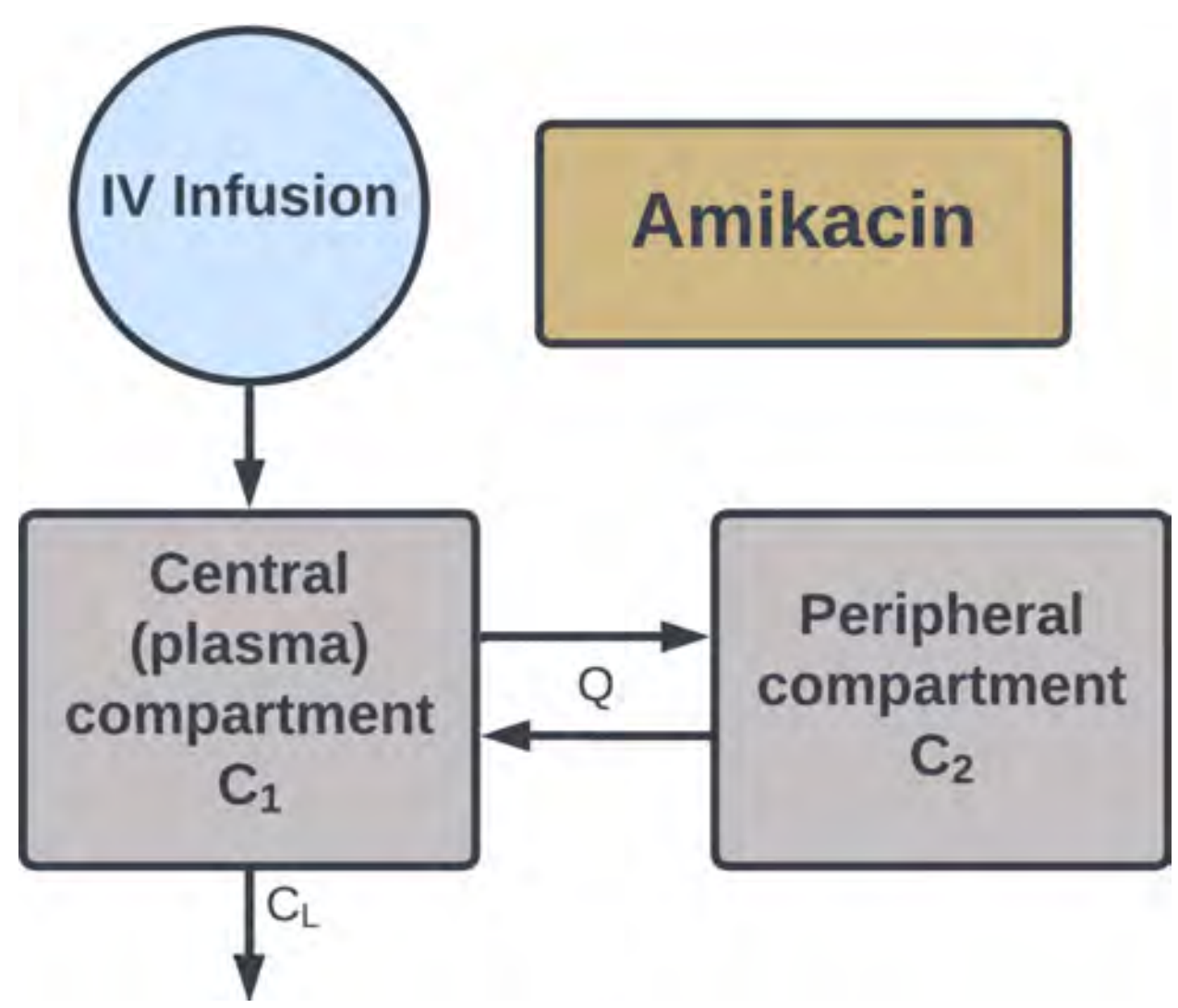

Figure S2: Schematic of the two-compartment population pharmacokinetic model for amikacin, showing drug transfer between the central (C_1) and peripheral (C_2) compartments, systemic clearance (`CL`), and intercompartmental transfer (`Q`).

C.4 Amikacin model AUC distributions

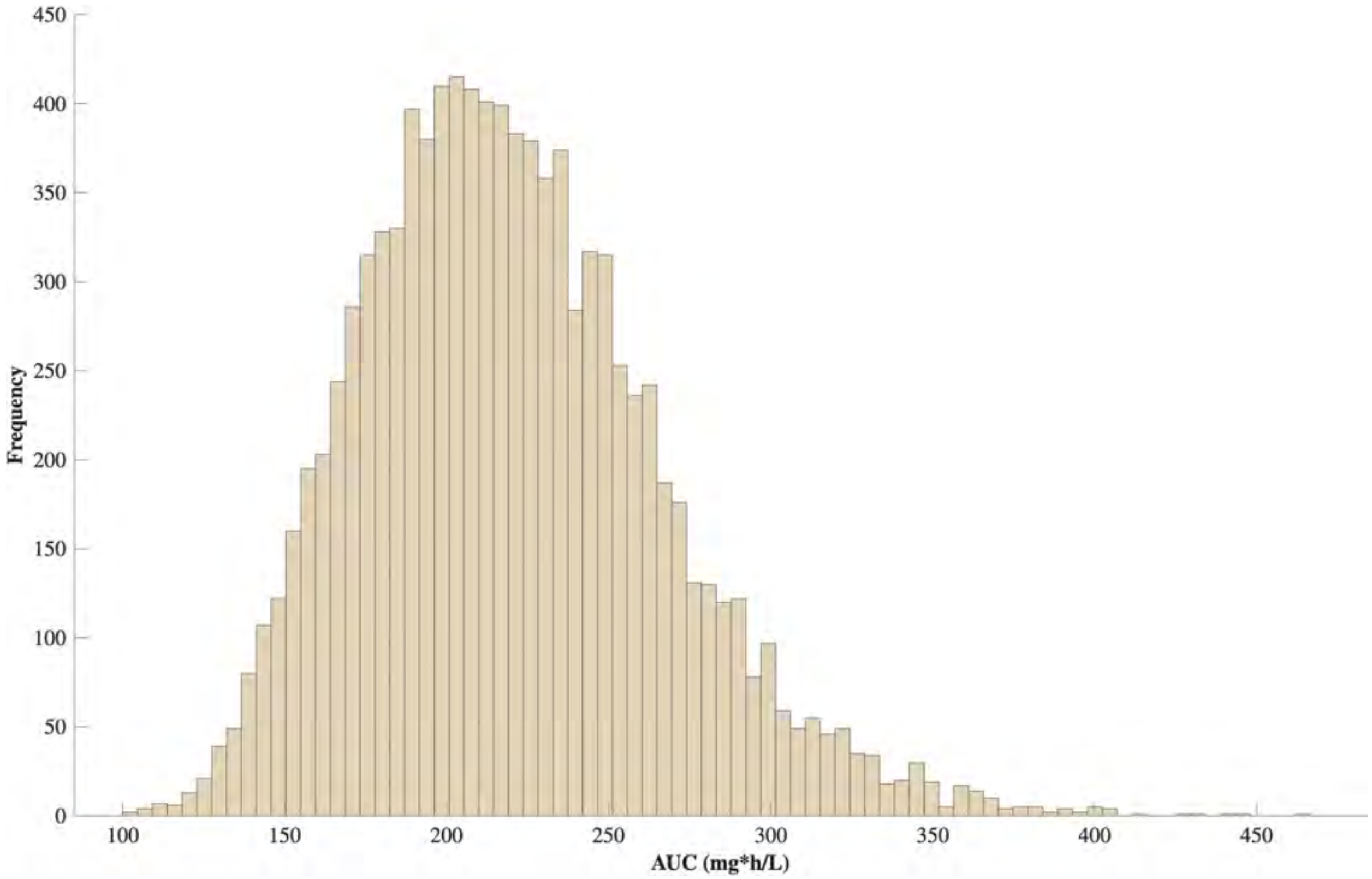

Figure S3: Steady-state AUC_{0-24} distributions across 10,000 simulated patient profiles for amikacin at 15 mg/kg q24h. Distributions reflect interindividual variability drawn from the parameter distributions in Table S2.

C.5 Amikacin model concentration time-curves

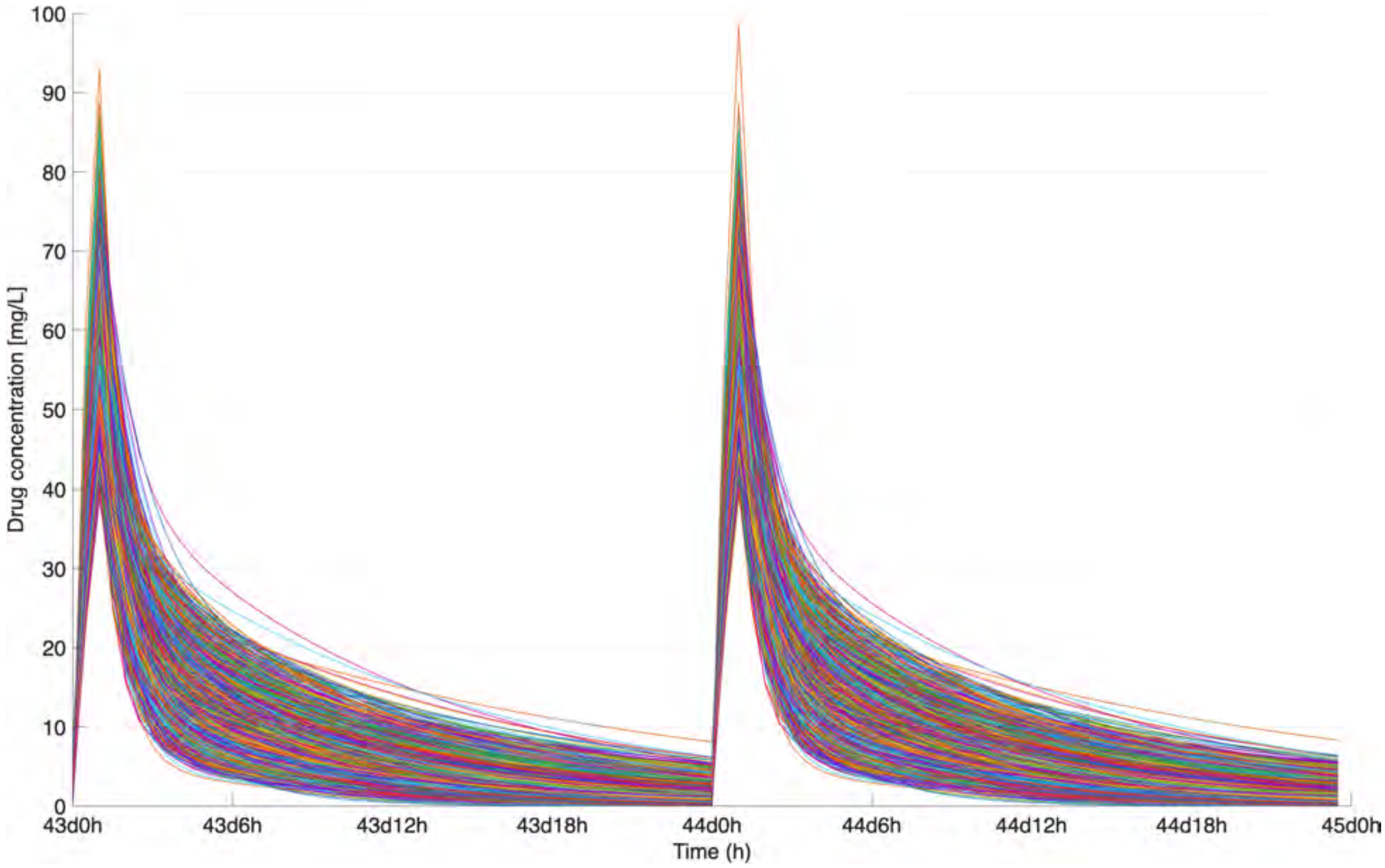

Figure S4: Simulated steady-state central compartment concentration-time profiles for amikacin across 10,000 virtual patients receiving 15 mg/kg q24h.

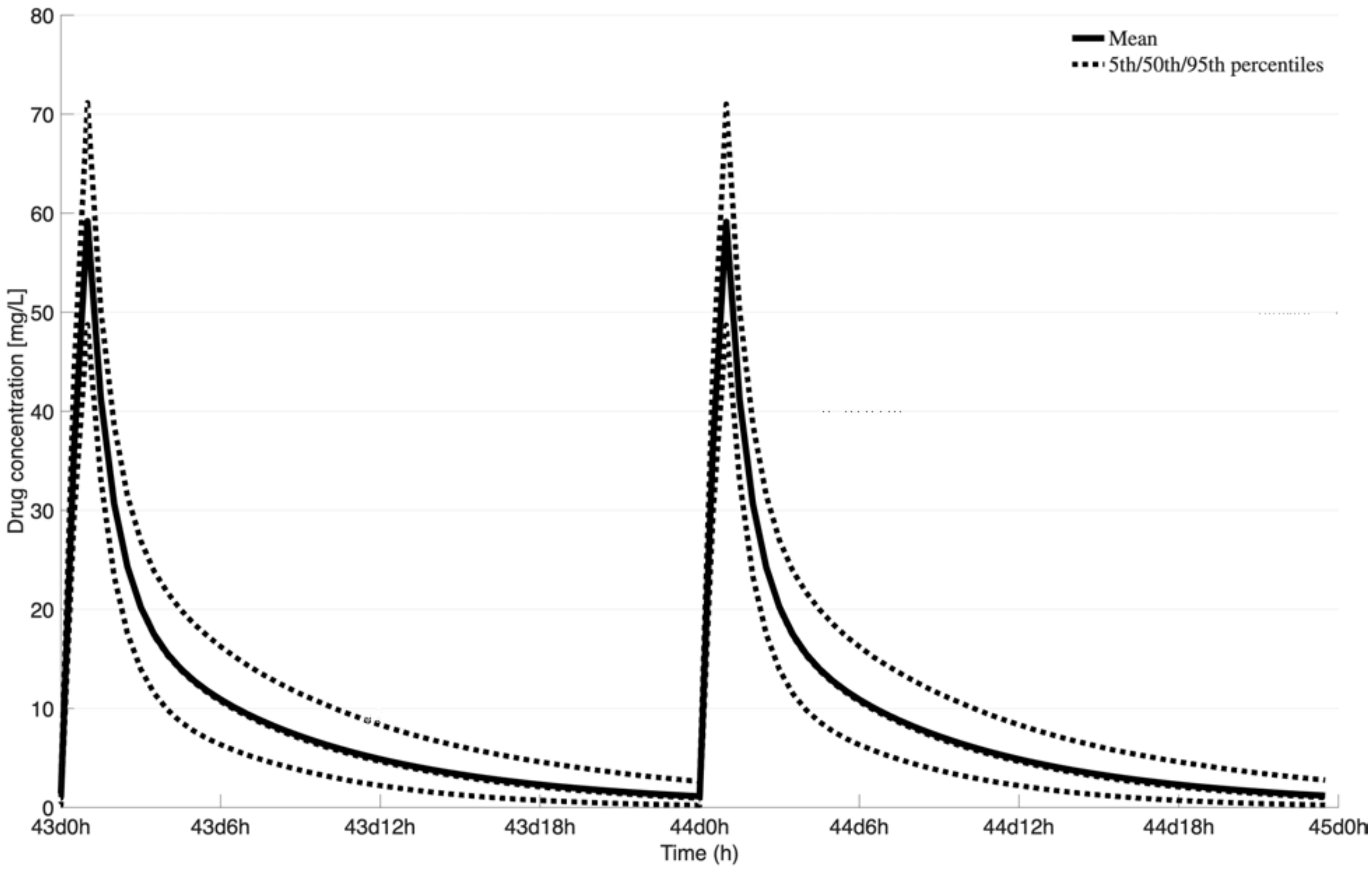

Figure S5: Simulated steady-state central compartment concentration-time profiles for amikacin showing the mean and 90% prediction interval across 10,000 virtual patients receiving 15 mg/kg q24h.

D Bedaquiline

D.1 Bedaquiline model parameters

Table S3: Population pharmacokinetic parameters for the ten-compartment bedaquiline model. Values represent typical population means; IIV is expressed as percent coefficient of variation ($\%CV$).

Parameter	Mean	IIV % CV	Units	Parameter	Mean	IIV % CV	Units
V_C	120	51	L	Q_2	6.8	26	L/h
V_2	79	27	L	Q_3	7.3	59	L/h
V_3	750	45	L	k_a	0.9	29	1/h
CL	6.7	30	L/h	k_tr	2.6	49	1/h

V_C is volume in central compartment; V_2 is volume in peripheral 2 compartment; V_3 is volume in peripheral 3 compartment; CL is clearance rate constant; Q_2 is inter-compartmental clearance constant between central and peripheral 2; Q_3 is inter-compartmental clearance constant between central and peripheral 3 compartment; k_a is first order absorption rate constant; k_tr is transit rate constant between transit compartments.

D.2 Bedaquiline model equations

This model was recreated from the model proposed in [27]. The parameter values used in the patient simulations were calculated using Equation 4. The model structure was a 10 compartment model with an absorption compartment ($q0$), 5 transit compartments ($q1 - q5$), a final transit compartment (q), a central compartment (C), and two peripheral compartments (C_2 & C_3) as shown in Figure S6.

$$\frac{d(q_0)}{dt} = -\texttt{k\_tr} \cdot (q0) \tag{7}$$

$$\frac{d(q_i)}{dt} = \texttt{k\_tr}\Big((q_{i-1}) - (q_i)\Big), \quad i = 1, \ldots, 5 \tag{8}$$

$$\frac{d(q)}{dt} = -\texttt{k\_a} \cdot (q) + \texttt{k\_tr} \cdot (q_5) \tag{9}$$

$$\frac{d(C)}{dt} = \frac{1}{\texttt{V\_C}}\Big[\texttt{k\_a} \cdot (q) - \texttt{Q\_2}\big((C) - (C_2)\big) - \texttt{Q\_3}\big((C) - (C_3)\big) - \texttt{CL} \cdot (C)\Big] \tag{10}$$

$$\frac{d(C_2)}{dt} = \frac{1}{\texttt{V\_2}}\Big[\texttt{Q\_2}\big((C) - (C_2)\big)\Big] \tag{11}$$

$$\frac{d(C_3)}{dt} = \frac{1}{\texttt{V\_3}}\Big[\texttt{Q\_3}\big((C) - (C_3)\big)\Big] \tag{12}$$

D.3 BDQ model flowchart

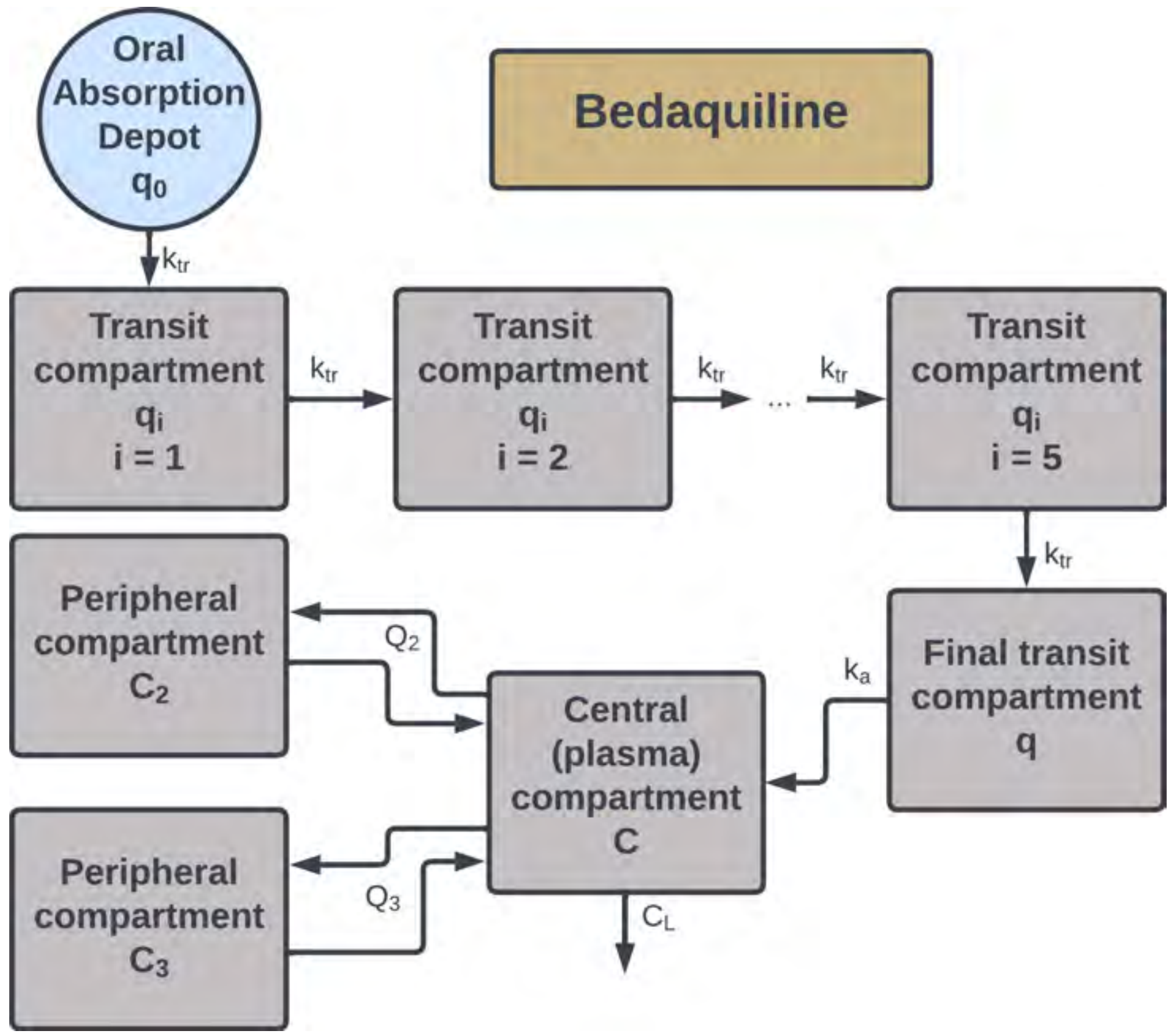

Figure S6: Schematic of the ten-compartment population pharmacokinetic model for bedaquiline, showing the absorption compartment (q_0), five transit compartments (q_1–q_5), final transit compartment (q), central compartment (C), and two peripheral compartments (C_2) and (C_3), with systemic clearance (`CL`) and intercompartmental transfer (`Q_2`, `Q_3`).

D.4 Bedaquiline model AUC distributions

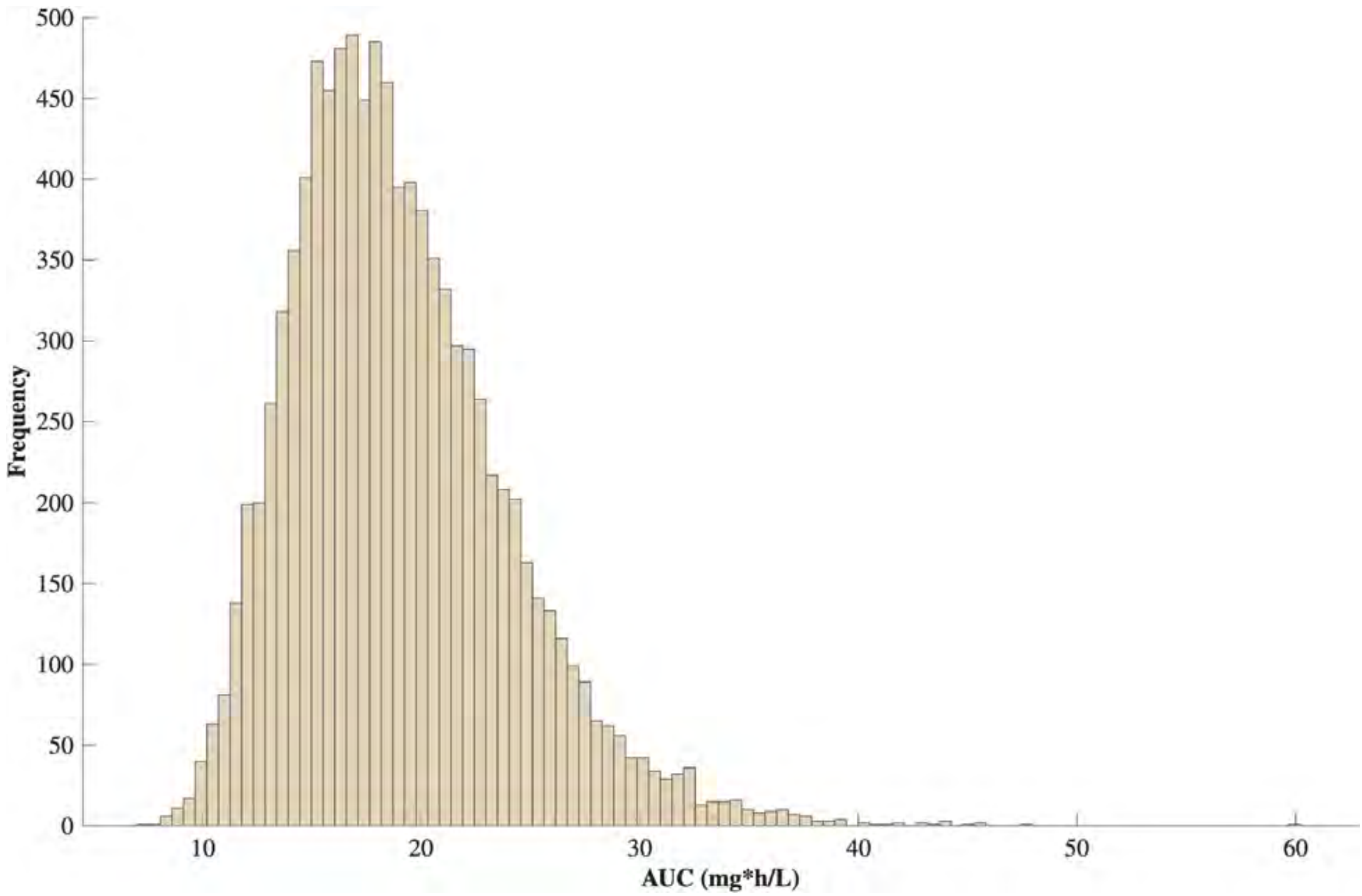

Figure S7: Steady-state AUC_{0-24} distributions across 10,000 simulated patient profiles for bedaquiline at 400 mg q24h for two weeks followed by 200 mg three times weekly. Distributions reflect interindividual variability drawn from the parameter distributions in Table S3.

D.5 Bedaquiline model concentration time-courses

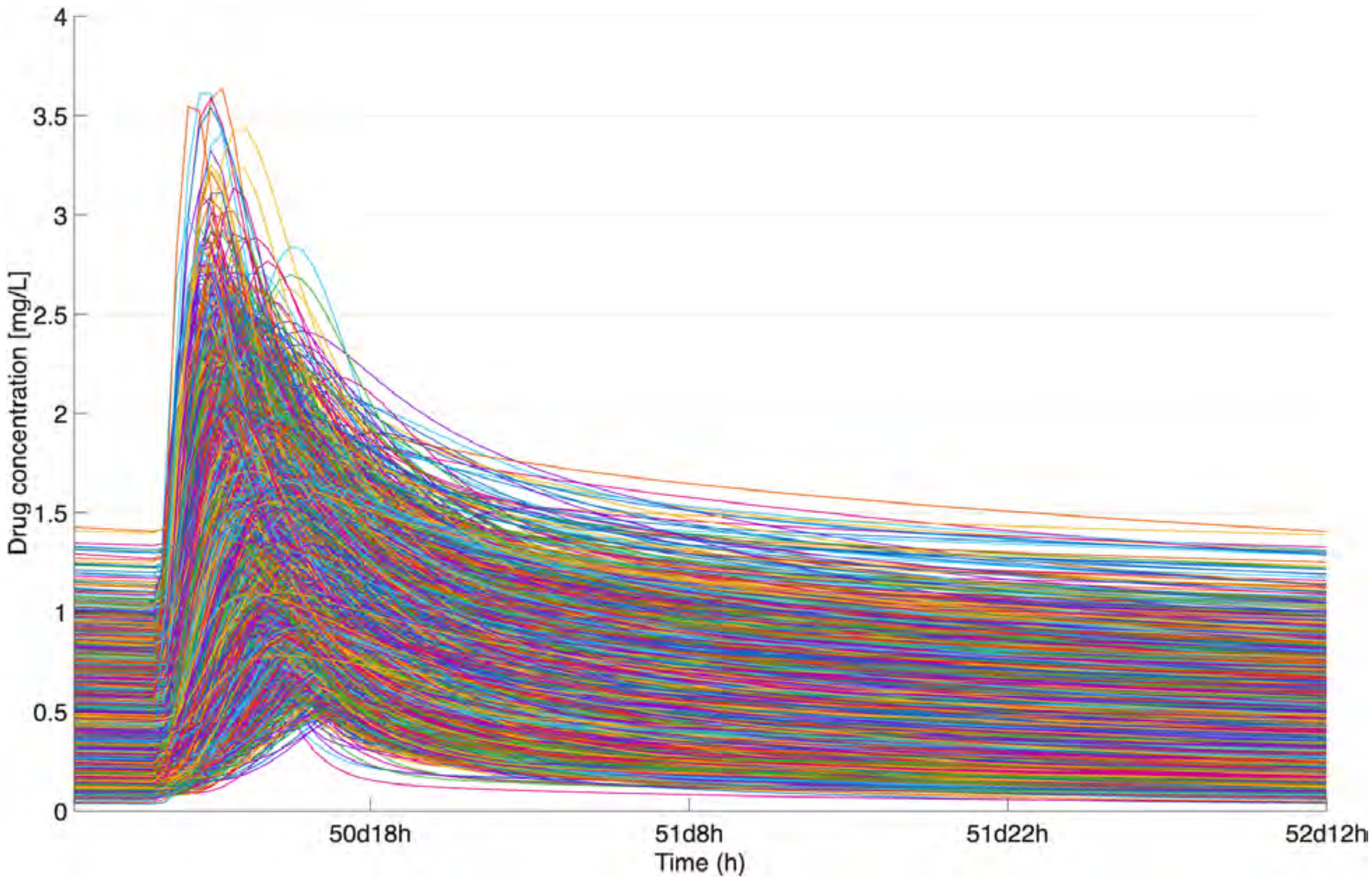

Figure S8: Simulated steady-state central compartment concentration-time profiles for bedaquiline across 10,000 virtual patients during the maintenance phase of 200 mg three times weekly (following 400 mg q24h for two weeks).

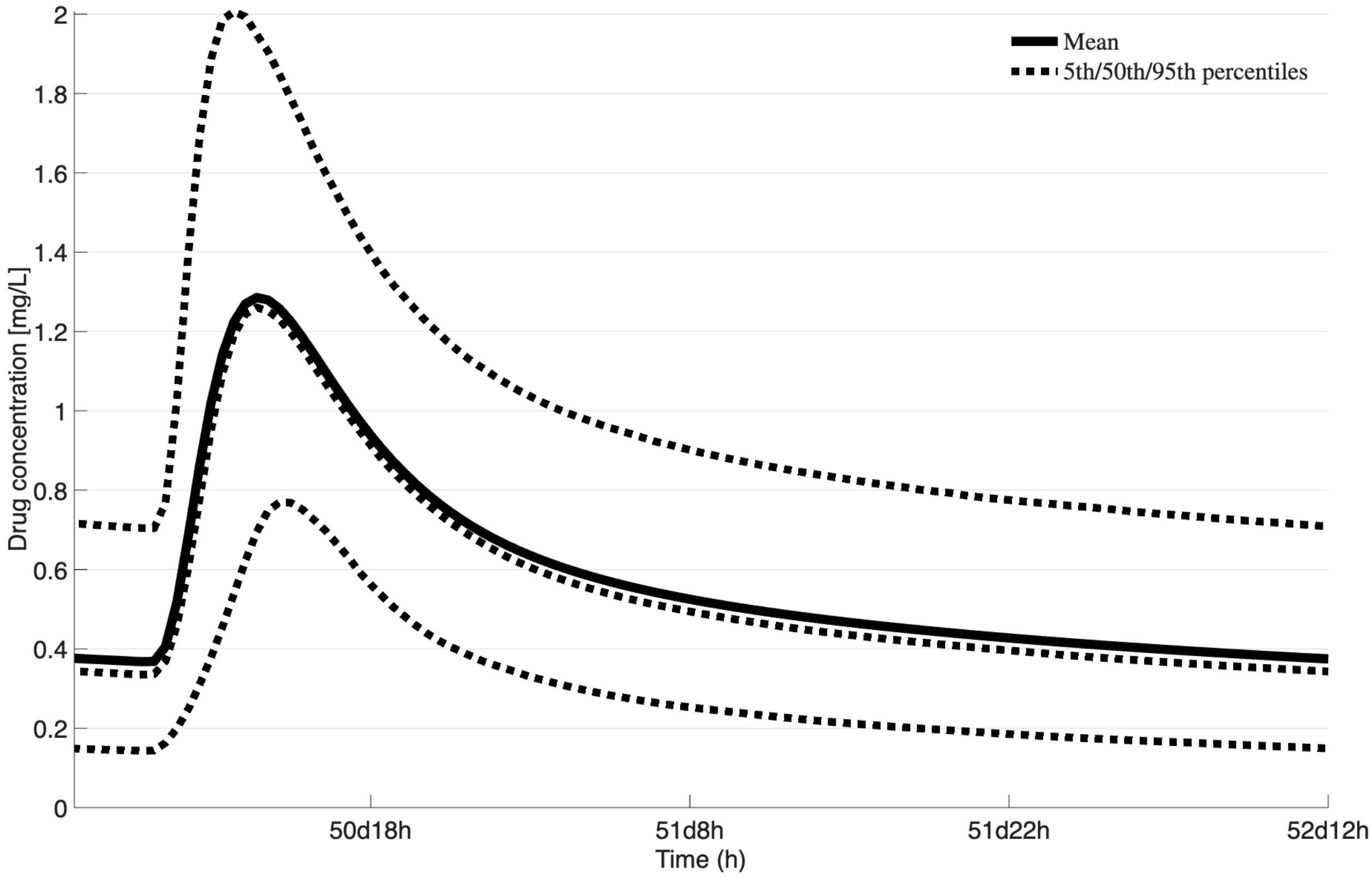

Figure S9: Simulated steady-state central compartment concentration-time profiles for bedaquiline showing the mean and 90% prediction interval across 10,000 virtual patients during the maintenance phase of 200 mg three times weekly (following 400 mg q24h for two weeks).

E Clofazimine

E.1 Clofazimine model parameters

Table S4: Population pharmacokinetic parameters for the three-compartment clofazimine model. Values represent typical population means; IIV and IOV are expressed as percent coefficient of variation ($\%CV$).

Parameter	Mean	IIV % CV	IOV % CV	Units
V_C	262	23.5	–	L
V_p1	10500	29.6	–	L
V_p2	889	54.6	–	L
CL/F	11.5	25.6	–	L/h
n	4.75	–	–	[–]
Q1/F	56.3	–	–	L/h
Q2/F	86	–	–	L/h
k_a	0.209	–	32.6	1/h
MTT	1.41	–	46.6	1/h

V_C is volume in central compartment; V_p1 is volume in peripheral 1 compartment; V_p2 is volume in peripheral 2 compartment; CL/F is clearance rate constant; Q1/F is inter-compartmental clearance constant between central and peripheral 1; Q2/F is inter-compartmental clearance constant between central and peripheral 2 compartment; k_a is first order absorption rate constant; MTT is the mean transit time between transit compartments; n is the number of transit compartments.

E.2 Clofazimine model equations

This model was recreated from the model proposed in [1]. The parameter values used in the patient simulations were calculated using Equation 4. The model structure was a 4 compartment model with an absorption compartment ($q0$), a central compartment (C), and two peripheral compartments (C_{p1} & C_{p2}).

$$\texttt{k\_tr} = \frac{\texttt{n}+1}{\texttt{MTT}} \tag{13}$$

$$\texttt{q0\_frac} = \Big(\texttt{k\_tr} \cdot t\Big)^{\texttt{n}} \tag{14}$$

$$\frac{d(q0)}{dt} = \texttt{dose} \cdot \texttt{F} \cdot \texttt{k\_tr} \cdot \texttt{q0\_frac} - \texttt{k\_a} \cdot (q0) \tag{15}$$

$$\frac{d(C)}{dt} = \frac{1}{\texttt{V\_C}}\Big[\texttt{k\_a} \cdot (q0) - \texttt{Q1/F}\big(C - C_{p1}\big) - \texttt{Q2/F}\big(C - C_{p2}\big) - \texttt{CL} \cdot (C)\Big] \tag{16}$$

$$\frac{d(C_{p1})}{dt} = \frac{1}{\texttt{V\_pq}}\Big[\texttt{Q1/F}\big(C - C_{p1}\big)\Big] \tag{17}$$

$$\frac{d(C_{p2})}{dt} = \frac{1}{\texttt{V\_p2}}\Big[\texttt{Q2/F}\big(C - C_{p2}\big)\Big] \tag{18}$$

E.3 Clofazimine model flowchart

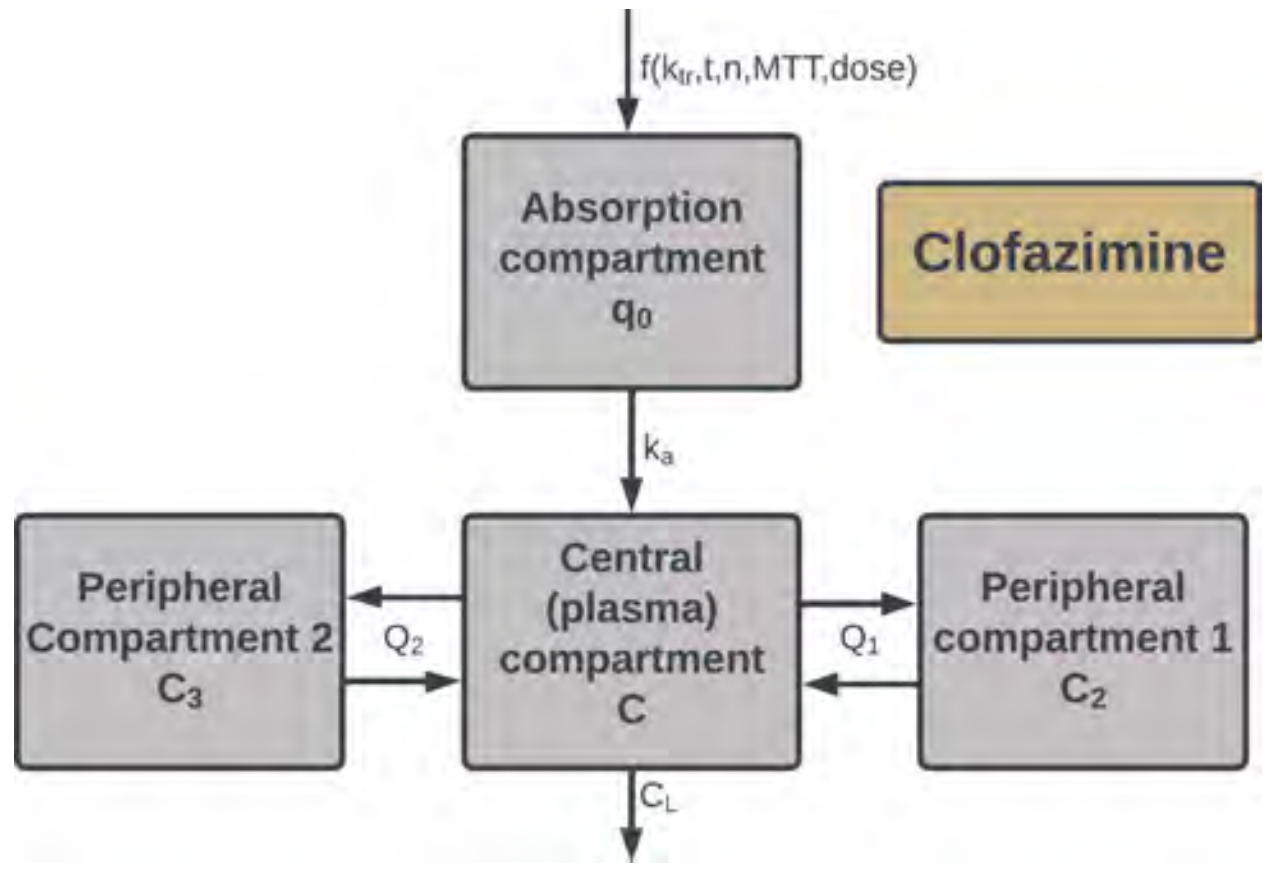

Figure S10: Schematic of the three-compartment population pharmacokinetic model for clofazimine, showing the absorption compartment (q_0), central compartment (C), and two peripheral compartments (C_{p1}) and (C_{p2}), with systemic clearance (`CL/F`) and intercompartmental transfer (`Q1/F`, `Q2/F`).

E.4 Clofazimine model AUC distributions

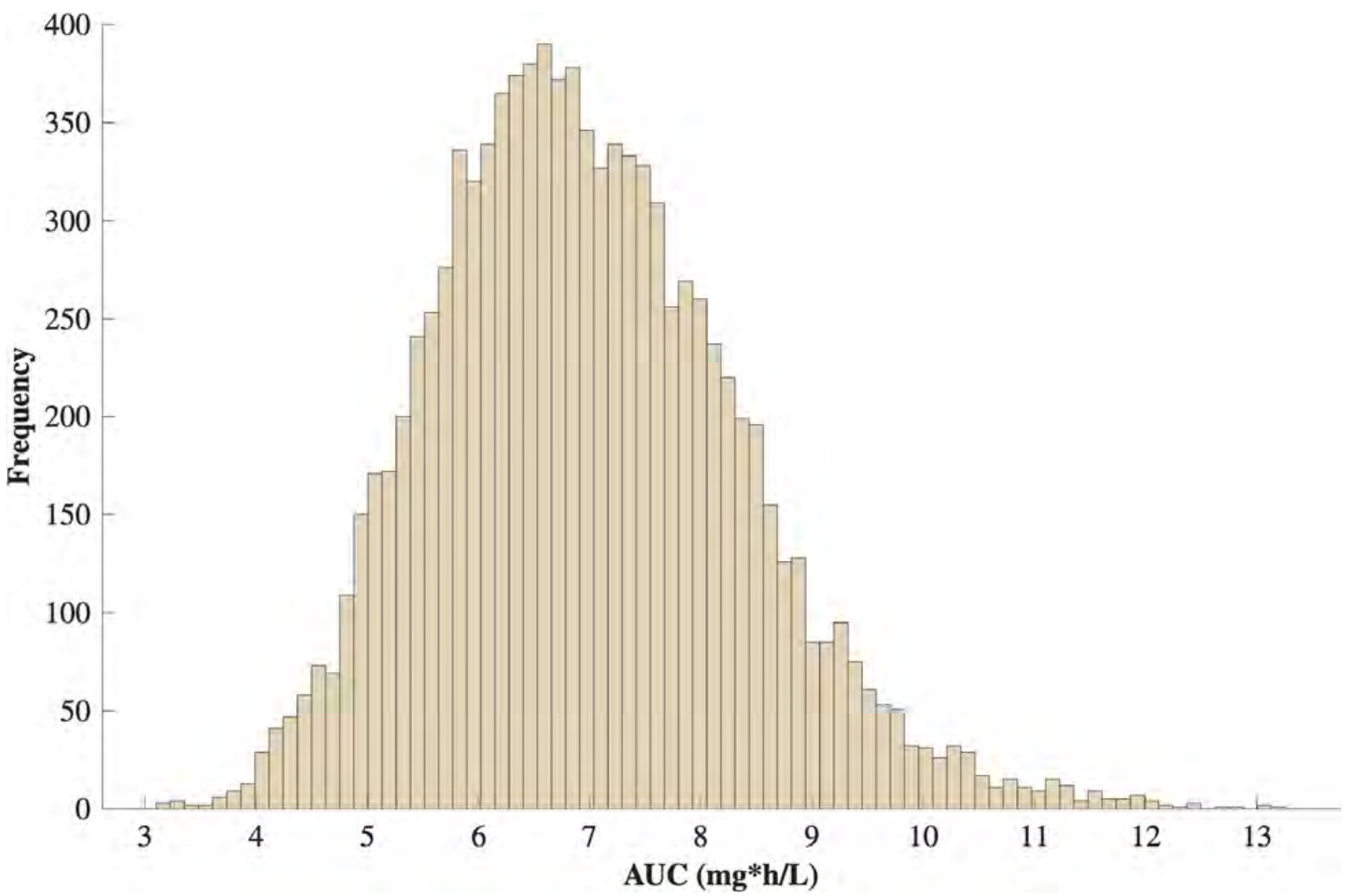

Figure S11: Steady-state AUC_{0-24} distributions across 10,000 simulated patient profiles for clofazimine at 100 mg q24h. Distributions reflect interindividual variability drawn from the parameter distributions in Table S4.

E.5 Clofazimine model concentration time-courses

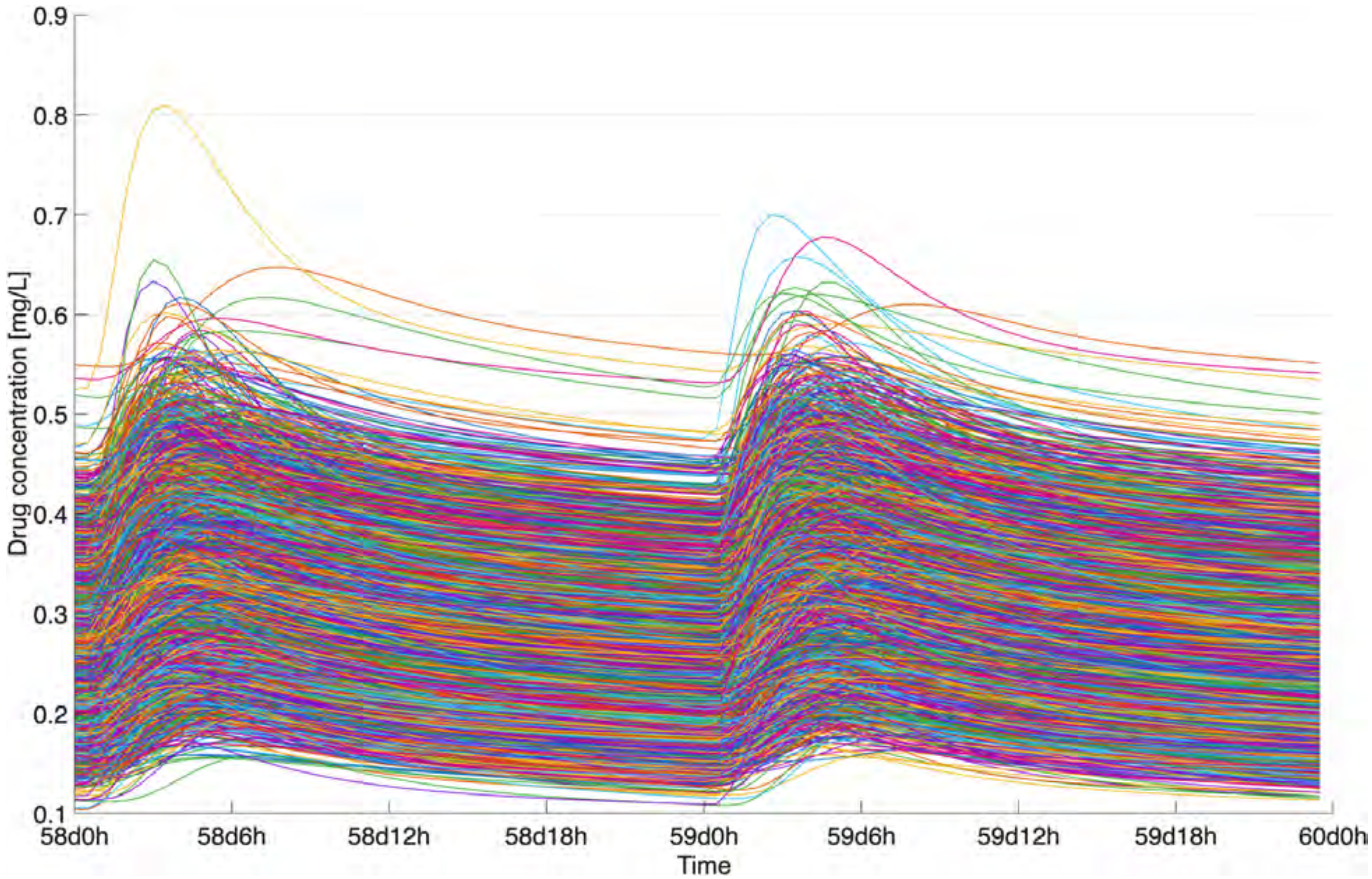

Figure S12: Simulated steady-state central compartment concentration-time profiles for clofazimine across 10,000 virtual patients receiving 100 mg q24h.

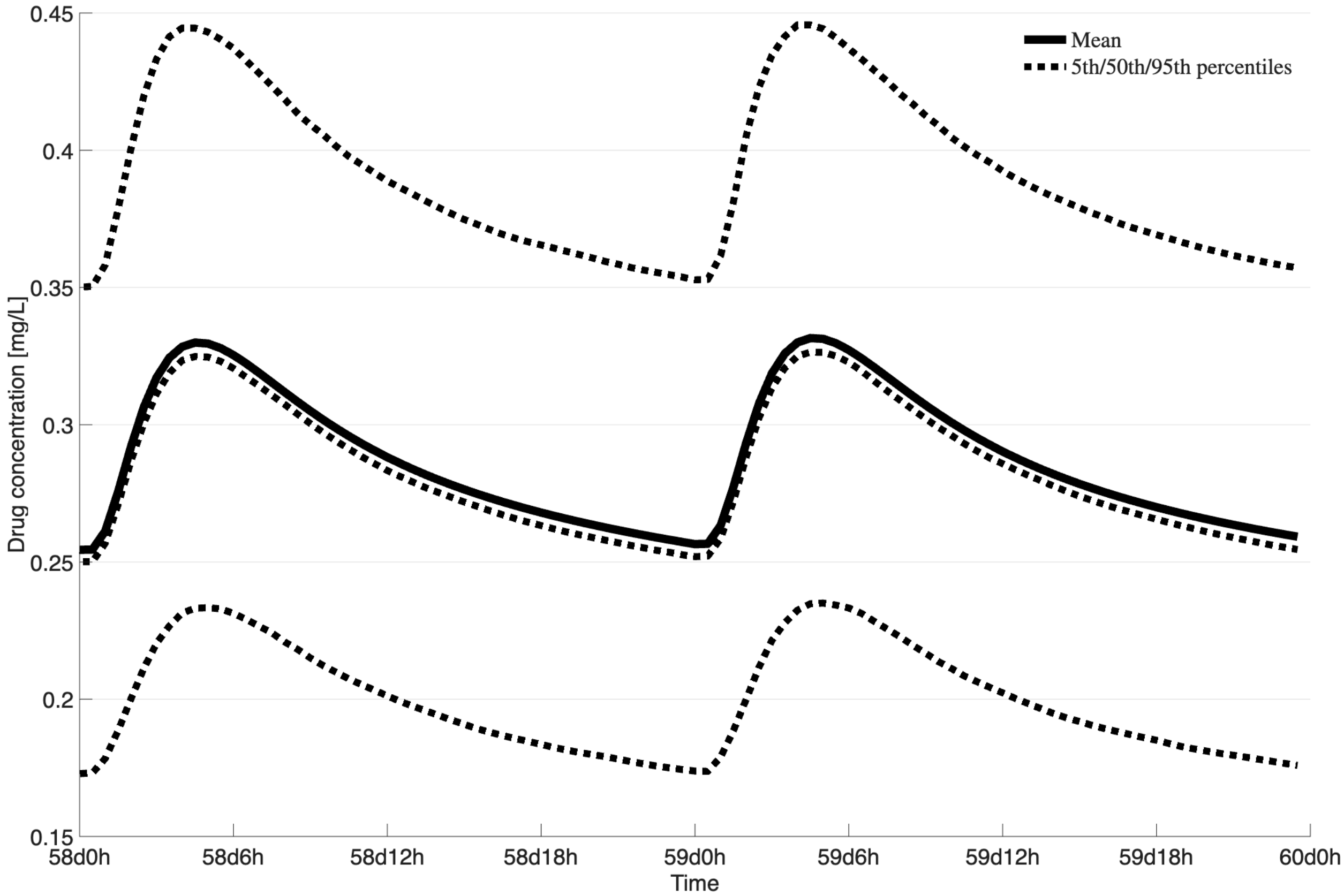

Figure S13: Simulated steady-state central compartment concentration-time profiles for clofazimine showing the mean and 90% prediction interval across 10,000 virtual patients receiving 100 mg q24h.

F Clarithromycin

F.1 Clarithromycin model parameters

Table S5: Population pharmacokinetic parameters for the three-compartment clarithromycin model. Values represent typical population means; IIV is expressed as percent coefficient of variation ($\%CV$).

Parameter	Mean	IIV % CV	Units
V1_F	204.7	12.0	L
V2_F	168.9	–	L
V3_F	67.1	34.0	L
CL/F	34.4	13.2	L/h
k_a	0.680	33.9	1/h
K_12	0.019	–	1/h
K_21	0.434	–	1/h
K_13	0.667	0.133	1/h
K_31	0.260	0.128	1/h

V1_F is volume in central compartment; V2_F is volume in peripheral compartment; V3_F is volume in epithelial compartment; CL/F is clearance rate constant; k_a is first order absorption rate constant; K_12 is inter-compartmental clearance constant between central and peripheral; K_21 is inter-compartmental clearance constant between peripheral and central; K_13 is inter-compartmental clearance constant between central and epithelial compartment; K_31 is inter-compartmental clearance constant between epithelial and central compartment.

F.2 Clarithromycin model equations

This model was recreated from the model proposed in [20]. The parameter values used in the patient simulations were calculated using Equation 4. The model structure was a 3 compartment model consisting of an absorption compartment (q_0), a central compartment (C), a peripheral compartment (C_2), and an epithelial compartment (C_3).

$$\frac{d(C)}{dt} = \frac{1}{\texttt{V1\_F}}\Big[\texttt{k\_a}\cdot q_0 - \texttt{K\_12}\cdot\texttt{V1\_F}\cdot C + \texttt{K\_21}\cdot\texttt{V2\_F}\cdot C_2 - \texttt{K\_13}\cdot\texttt{V1\_F}\cdot C + \texttt{K\_31}\cdot\texttt{V3\_F}\cdot C_3 - \texttt{CL/F}\cdot C\Big] \quad (19)$$

$$\frac{d(C_2)}{dt} = \frac{1}{\texttt{V2\_F}}\Big[\texttt{K\_12}\cdot\texttt{V1\_F}\cdot C - \texttt{K\_21}\cdot\texttt{V2\_F}\cdot C_2\Big] \quad (20)$$

$$\frac{d(C_3)}{dt} = \frac{1}{\texttt{V3\_F}}\Big[\texttt{K\_13}\cdot\texttt{V1\_F}\cdot C - \texttt{K\_31}\cdot\texttt{V3\_F}\cdot C_3\Big] \quad (21)$$

F.3 Clarithromycin model flowchart

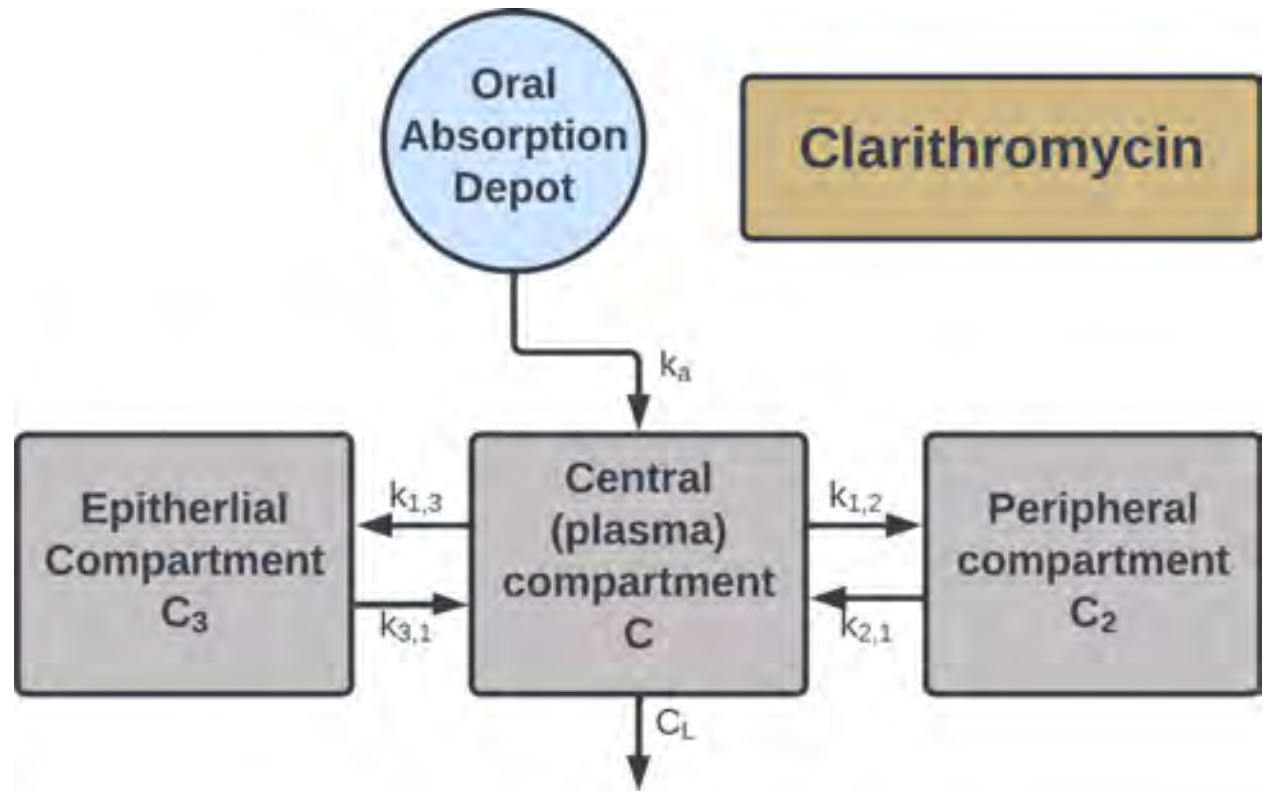

Figure S14: Schematic of the three-compartment population pharmacokinetic model for clarithromycin, showing the absorption compartment (q_0), central compartment (C), peripheral compartment (C_2), and epithelial compartment (C_3), with systemic clearance (`CL/F`) and intercompartmental transfer rate constants (`K_12`, `K_21`, `K_13`, `K_31`).

F.4 Clarithromycin model AUC distributions

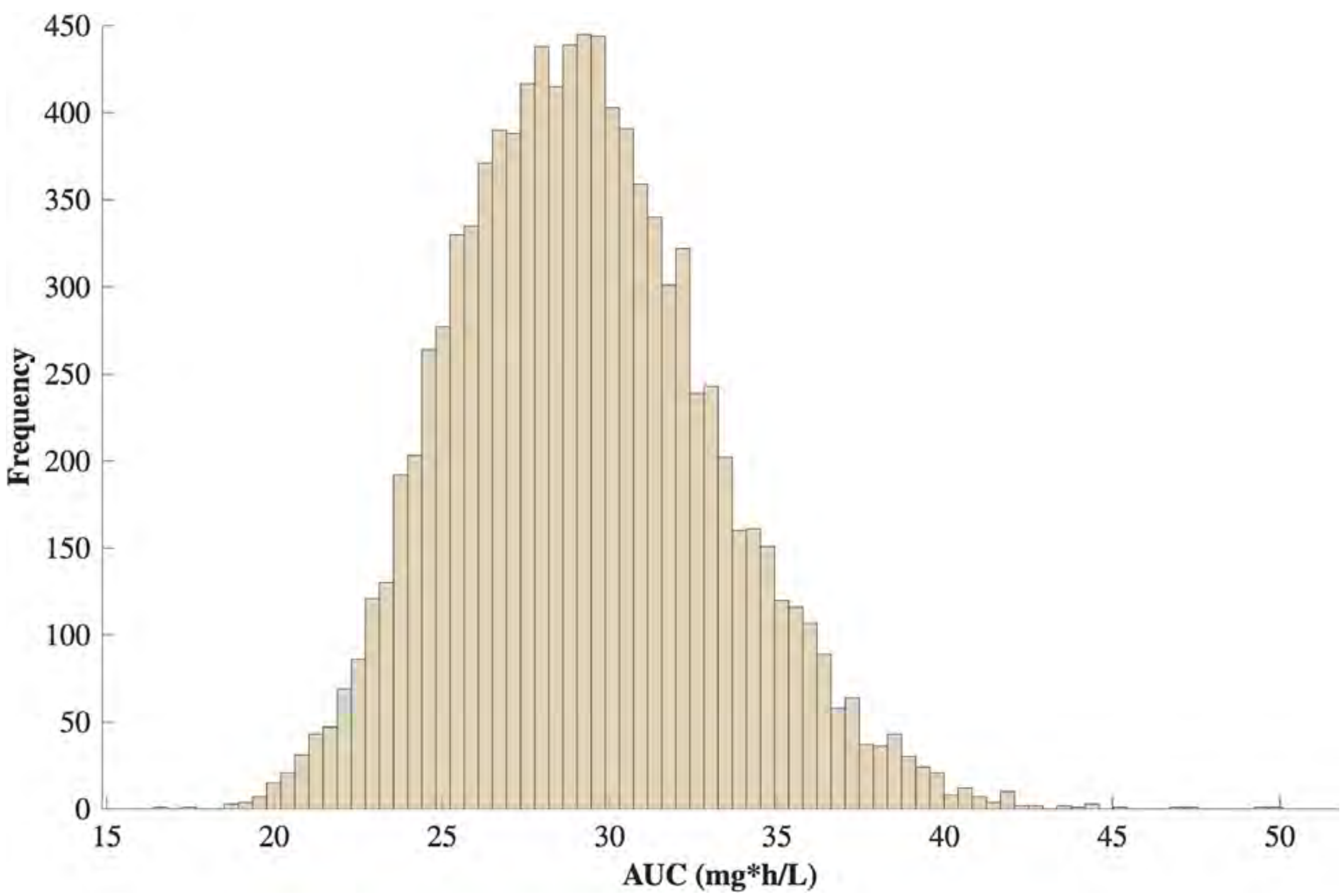

Figure S15: Steady-state $AUC_{0–24}$ distributions across 10,000 simulated patient profiles for clarithromycin at 500 mg q12h. Distributions reflect interindividual variability drawn from the parameter distributions in Table S5.

F.5 Clarithromycin model concentration time-courses

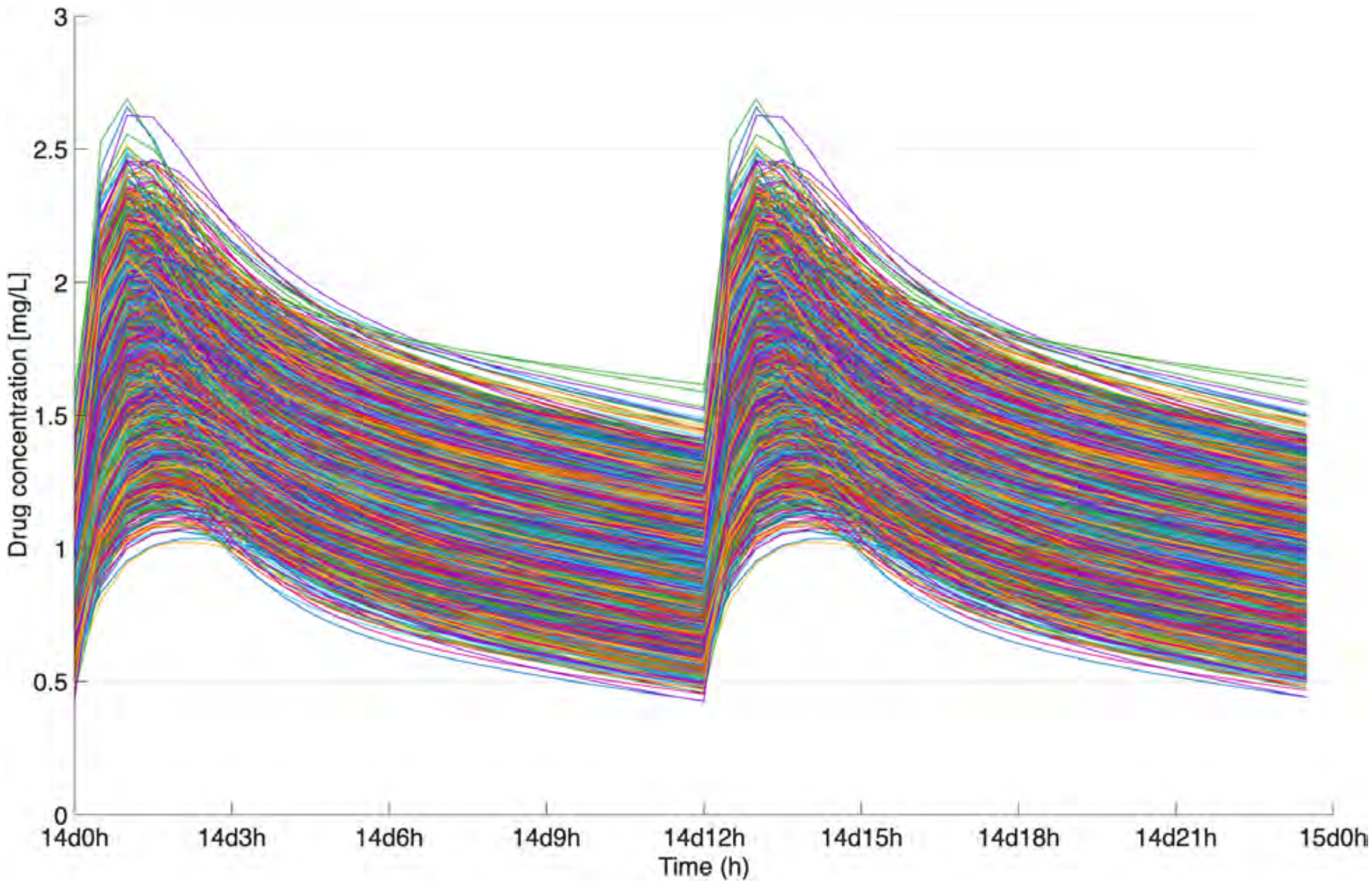

Figure S16: Simulated steady-state central compartment concentration-time profiles for clarithromycin across 10,000 virtual patients receiving 500 mg q12h.

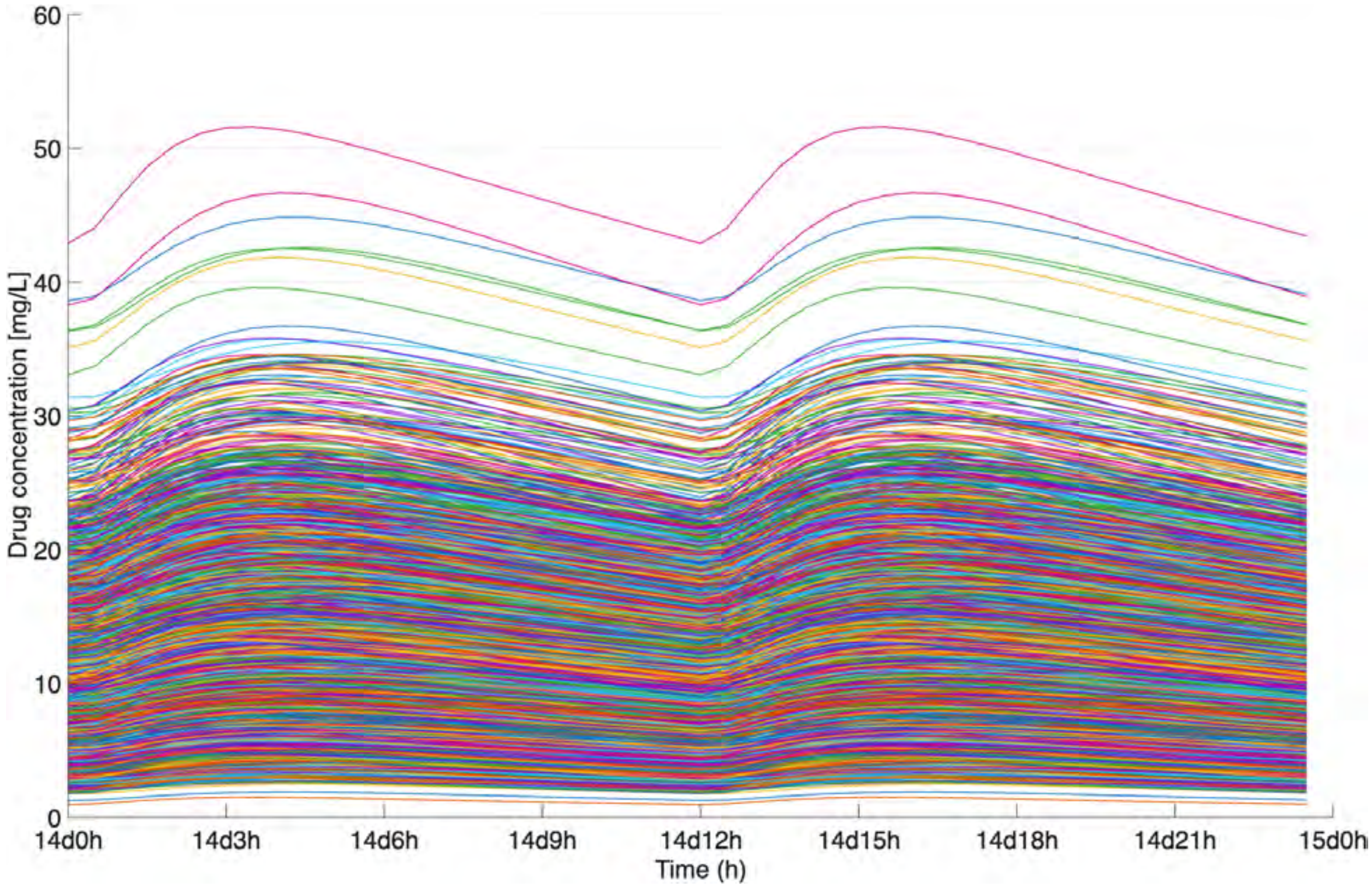

Figure S17: Simulated steady-state epithelial lining fluid concentration-time profiles for clarithromycin across 10,000 virtual patients receiving 500 mg q12h.

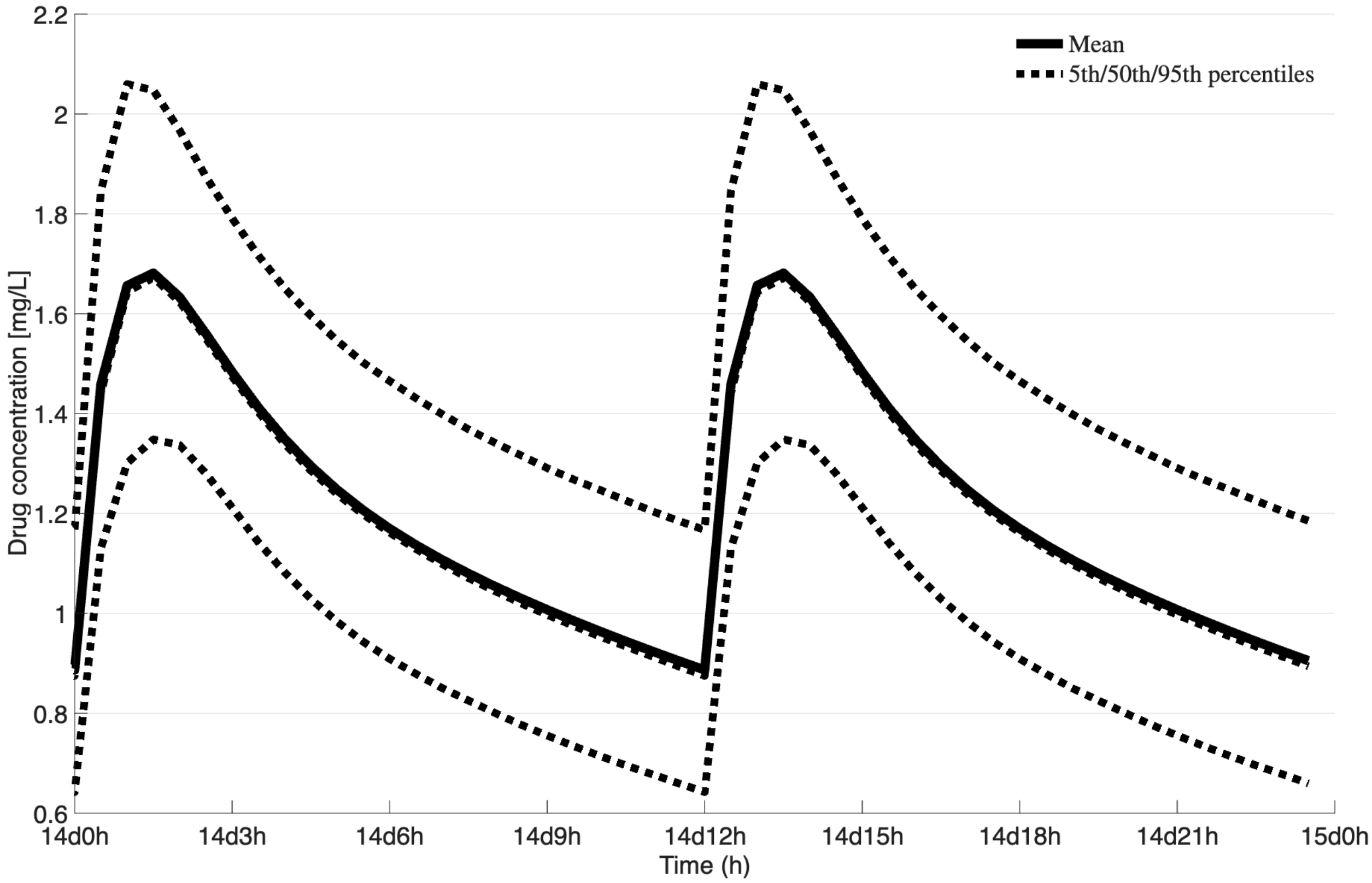

Figure S18: Simulated steady-state central compartment concentration-time profiles for clarithromycin showing the mean and 90% prediction interval across 10,000 virtual patients receiving 500 mg q12h.

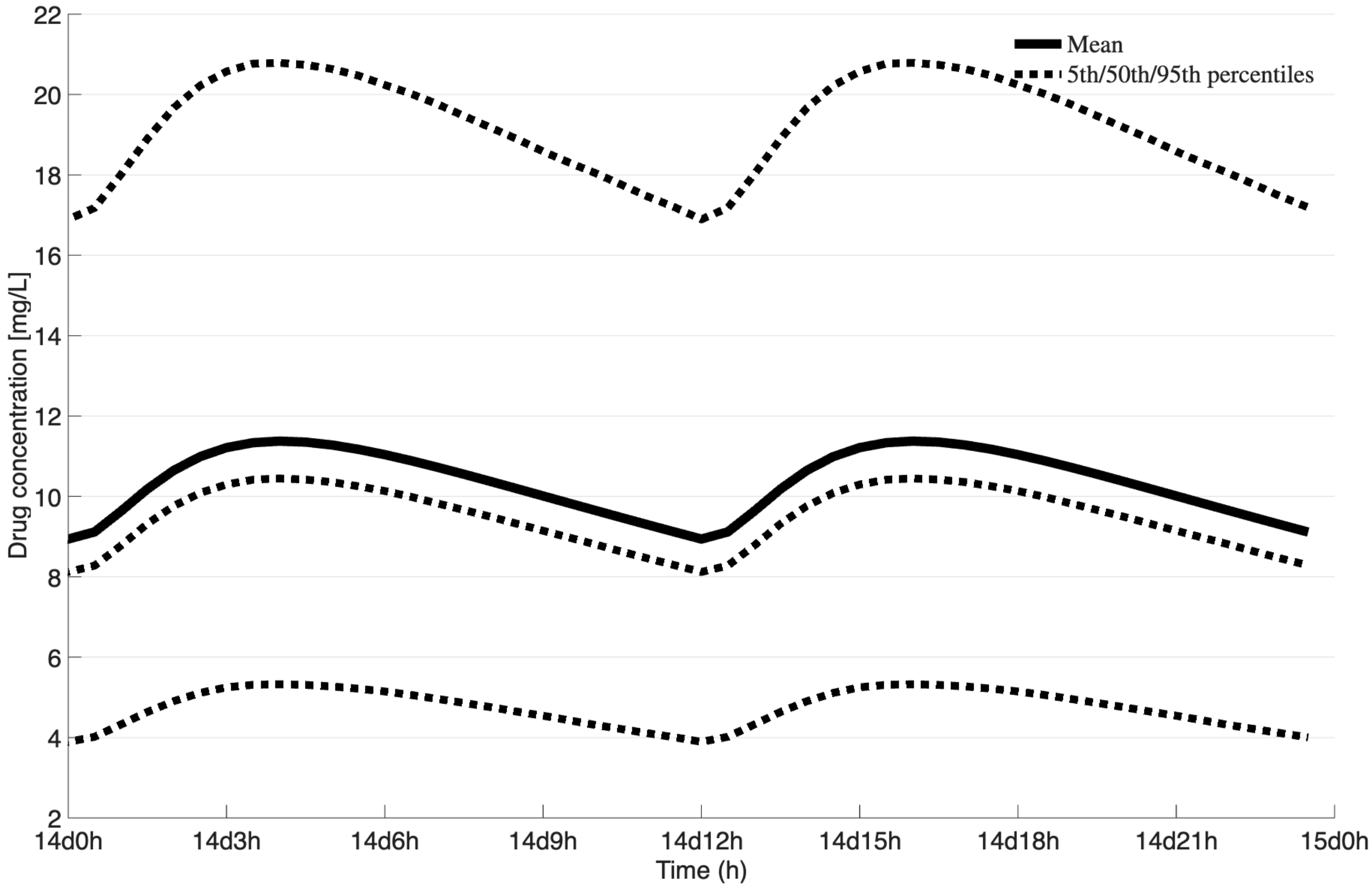

Figure S19: Simulated steady-state epithelial lining fluid concentration-time profiles for clarithromycin showing the mean and 90% prediction interval across 10,000 virtual patients receiving 500 mg q12h.

G Ethambutol

G.1 Ethambutol model parameters

Table S6: Population pharmacokinetic parameters for the transit-compartment ethambutol model. Values represent typical population means; IIV and IOV are expressed as percent coefficient of variation ($\%CV$).

Parameter	Mean	IIV % CV	IOV % CV	Units
Vc_F	82.4	–	–	L
Vp_F	623	–	–	L
CL_F	39.9	20	36	L/h
Q_F	34.3	–	–	L/h
k_a	0.474	39	–	1/h
MTT	0.789	93	–	h
HIV	-0.154	–	–	–

Vc_F is the volume of distribution for the central compartment. Vp_F is the volume of distribution for the peripheral compartment. CL_F is the clearance rate for the antibiotic from the central compartment. Q_F is the inter-compartmental clearance rate between the central and peripheral compartment in both directions. k_a is the absorption rate from the absorption compartment to the central compartment. MTT is the mean transit time between transit compartments. HIV is the parameter that affects the bio-absorption of the antibiotic in the system. For this parameter, if the patient is randomly sampled in the model to have HIV, then the value that HIV is multiplied by in Equation 22 is 1, otherwise, it is 0.

G.2 Ethambutol model equations

This model was recreated from the model proposed in [24]. The parameter values used in the patient simulations were calculated using Equation 4. The model structure consists of a transit-compartment absorption input, a central compartment (C), and a peripheral compartment (C_p), with an HIV covariate effect on bioavailability.

$$\mathtt{F} = 1 \cdot (1 - \mathtt{HIV} \cdot (1|0)) \tag{22}$$

$$\mathtt{k\_tr} = \frac{\mathtt{n} + 1}{\mathtt{MTT}} \tag{23}$$

$$\mathtt{q0\_frac} = \Big(\mathtt{k\_tr} \cdot t\Big)^{\mathtt{n}} \cdot \Big(\exp\left(-\mathtt{k\_tr} \cdot t\right)\Big)/\Gamma_{\mathtt{n}} \tag{24}$$

$$\frac{d(q)}{dt} = \mathtt{F} \cdot \mathtt{dose} \cdot \mathtt{k\_tr} \cdot \mathtt{q0\_frac} \tag{25}$$

Where k_tr was calculated using Equation 23, and q0_frac was calculated using Equation 24. n was set to 1 to represent 1 transit compartment.

$$\frac{d(C)}{dt} = \mathtt{k\_a} \cdot q - \Big(\mathtt{Q\_F} \cdot \frac{C}{\mathtt{Vc\_F}}\Big) + \Big(\mathtt{Q\_F} \cdot \frac{C_p}{\mathtt{Vp\_F}}\Big) - \Big(\mathtt{CL\_F} \cdot \frac{C}{\mathtt{Vc\_F}}\Big) \tag{26}$$

$$\frac{d(C_p)}{dt} = \mathtt{Q\_F} \cdot \frac{C}{\mathtt{Vc\_F}} - \Big(\mathtt{Q\_F} \cdot \frac{C_p}{\mathtt{Vp\_F}}\Big) \tag{27}$$

G.3 Ethambutol model flowchart

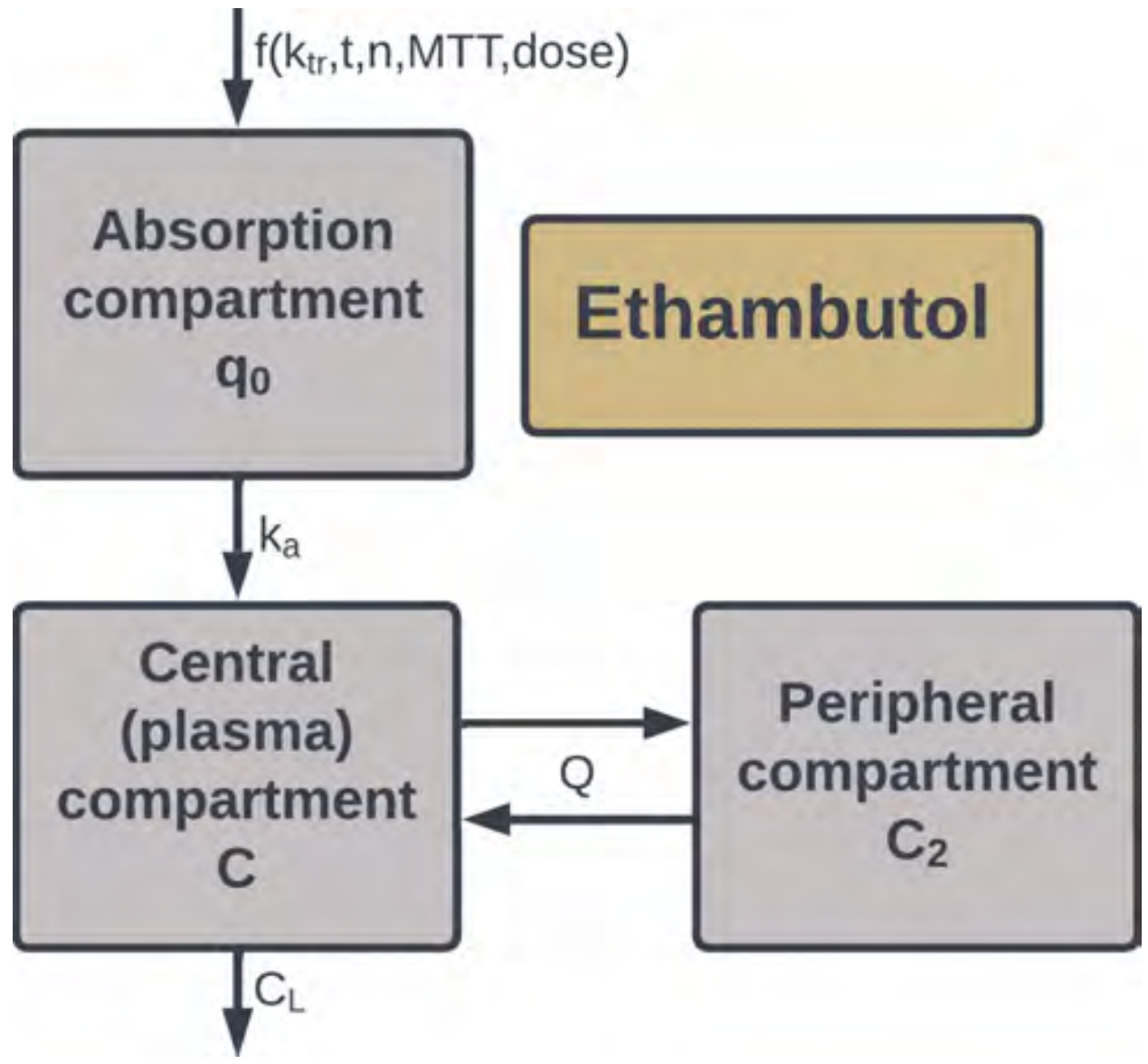

Figure S20: Schematic of the two-compartment population pharmacokinetic model for ethambutol with transit-compartment absorption, showing the absorption input (q), central compartment (C), and peripheral compartment (C_p), with systemic clearance (`CL_F`), intercompartmental clearance (`Q_F`), and HIV covariate effect on bioavailability (`F`).

G.4 Ethambutol model AUC distributions

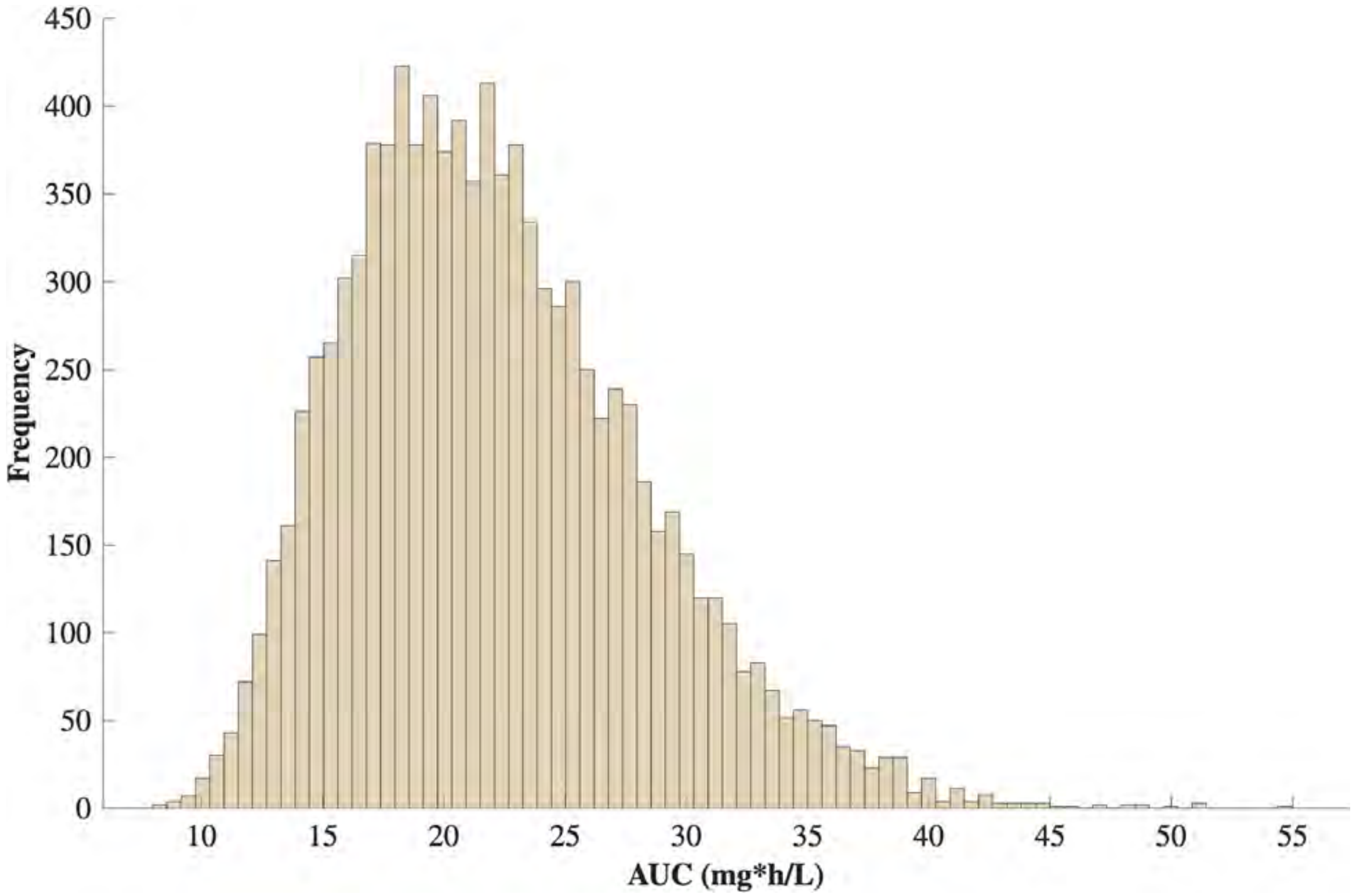

Figure S21: Steady-state AUC_{0-24} distributions across 10,000 simulated patient profiles for ethambutol at 15 mg/kg q24h. Distributions reflect interindividual variability drawn from the parameter distributions in Table S6.

G.5 Ethambutol model concentration time-courses

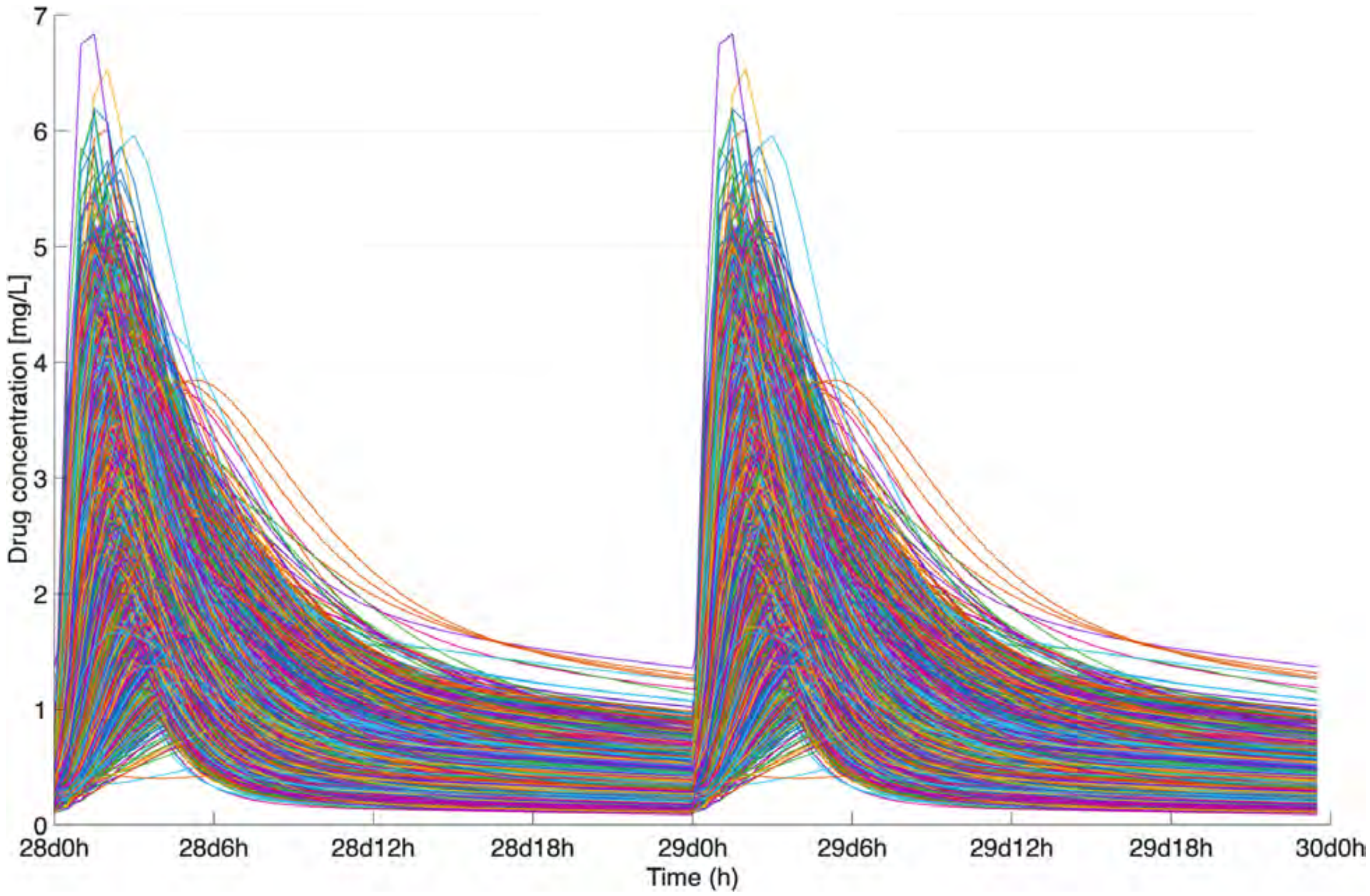

Figure S22: Simulated steady-state central compartment concentration-time profiles for ethambutol across 10,000 virtual patients at 15 mg/kg q24h.

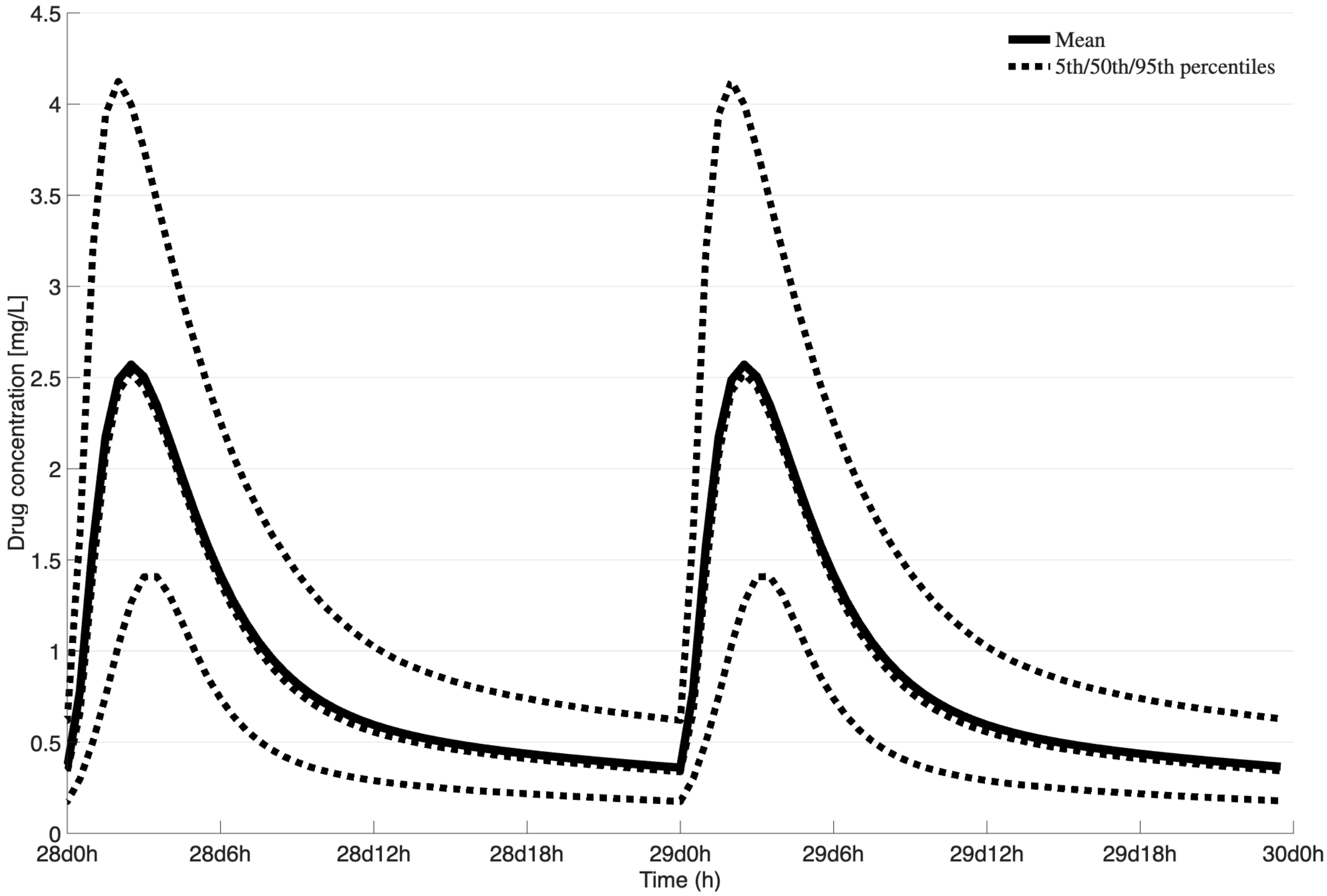

Figure S23: Simulated steady-state central compartment concentration-time profiles for ethambutol showing the mean and 90% prediction interval across 10,000 virtual patients at 15 mg/kg q24h.

H Cefoxitin

H.1 Cefoxitin model parameters

Table S7: Population pharmacokinetic parameters for the one-compartment cefoxitin model. Values are the typical population means reported in the original source [32]; interindividual variability (IIV) is expressed as percent coefficient of variation ($\%CV$).

Parameter	Mean	IIV % CV	Units
`V_c`	23.4	44.8	L
`CL`	10.9	56.3	L/h

The cefoxitin model parameters are presented in Table S7 as they appear in the reimplemented model. The structural parameters are the systemic clearance (`CL`) and the central volume of distribution (`V_c`). In the original analysis, none of the tested covariates—including body weight, body composition, serum creatinine, and the various estimates of glomerular filtration rate—significantly improved the model [32]; consequently, no covariate scaling is applied to the parameters here. For each simulated patient, `V_c` and `CL` are drawn independently from log-normal distributions about their population means according to the IIV model described in Equation 4.

H.2 Cefoxitin model equations

This model was recreated from the one-compartment model proposed in [32]. The model structure consists of a single central compartment (C) with first-order elimination, with drug administered as a short IV infusion described by Equation 1. The parameter values used in the patient simulations were generated using the interindividual variability model in Equation 4.

$$\frac{d(A)}{dt} = \texttt{infusion rate} - \texttt{CL} \cdot \frac{A}{\texttt{V\_c}} \tag{28}$$

A is the amount of cefoxitin in the central compartment.

$$C = \frac{A}{\texttt{V\_c}} \tag{29}$$

The reported plasma concentration C is obtained by dividing the central-compartment amount by the patient-specific volume of distribution.

H.3 Cefoxitin model flowchart

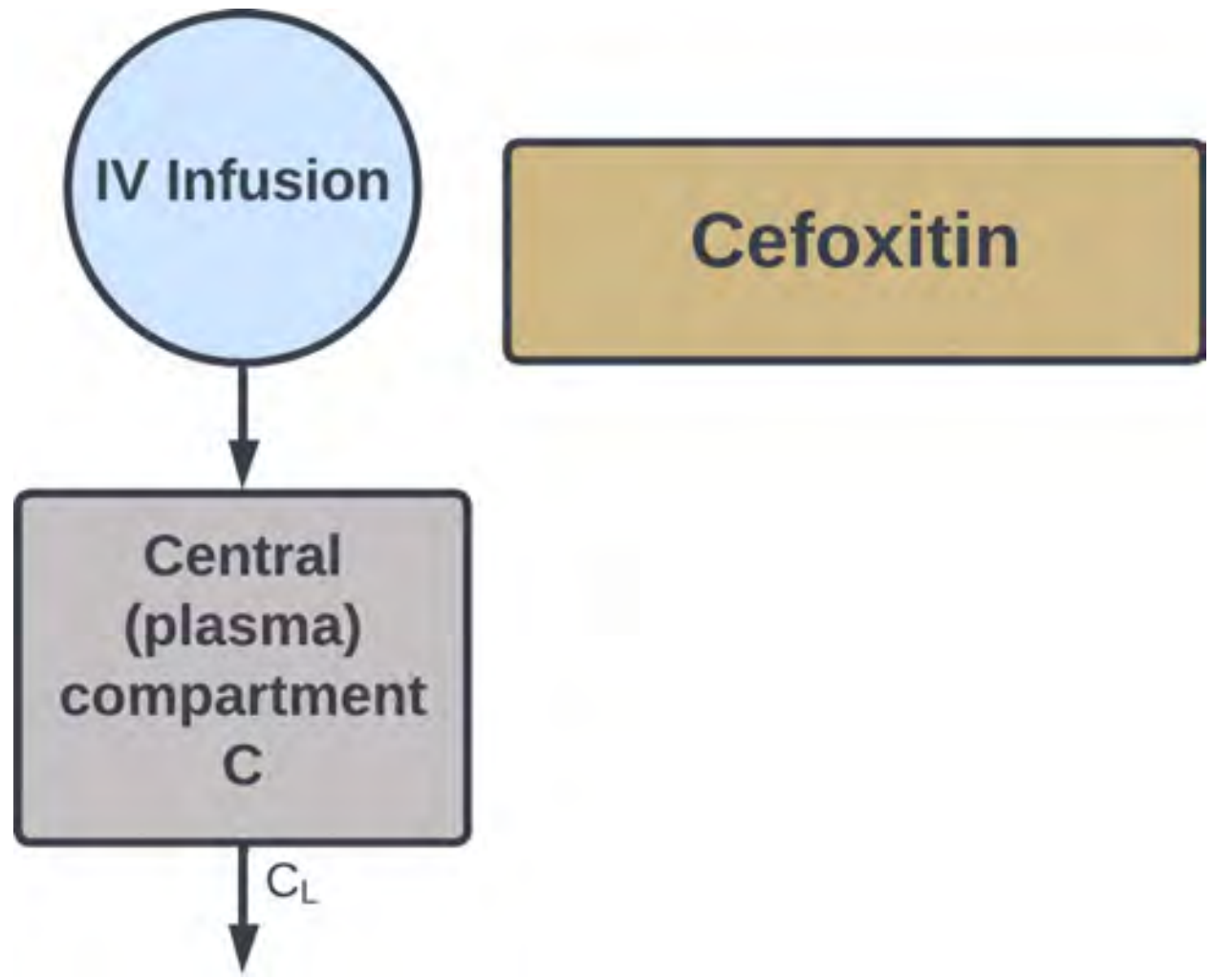

Figure S24: Schematic of the one-compartment population pharmacokinetic model for cefoxitin, showing the central compartment (C) with systemic clearance (CL) and a short IV infusion input. Drug is eliminated from the central compartment by a first-order process.

H.4 Cefoxitin model AUC distributions

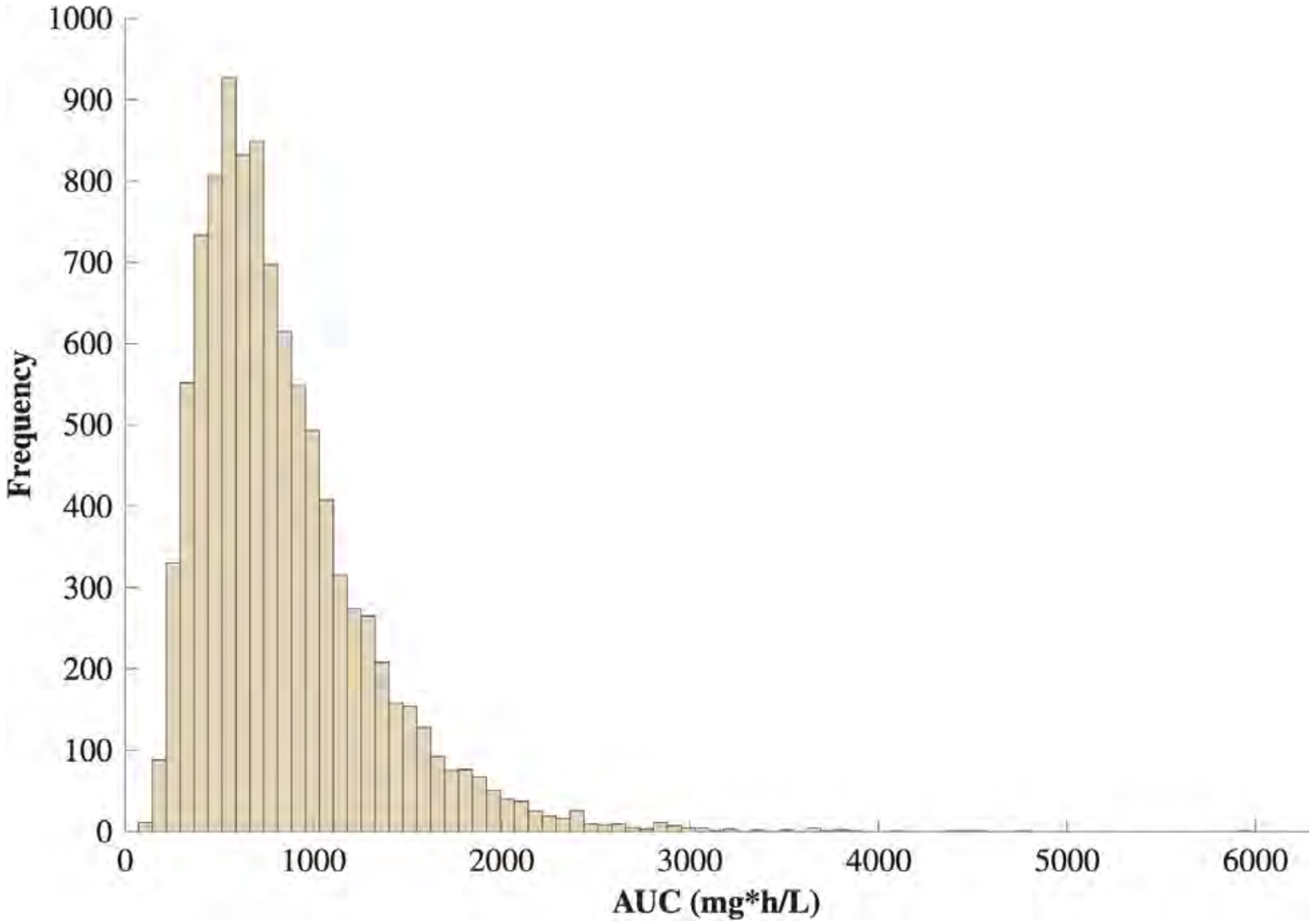

Figure S25: Steady-state AUC_{0-24} distribution across the simulated virtual-patient population for cefoxitin at 4 g q12h administered as a 3-hour IV infusion. The distribution reflects interindividual variability in `V_c` and `CL` drawn from the parameters in Table S7.

H.5 Cefoxitin model concentration time-courses

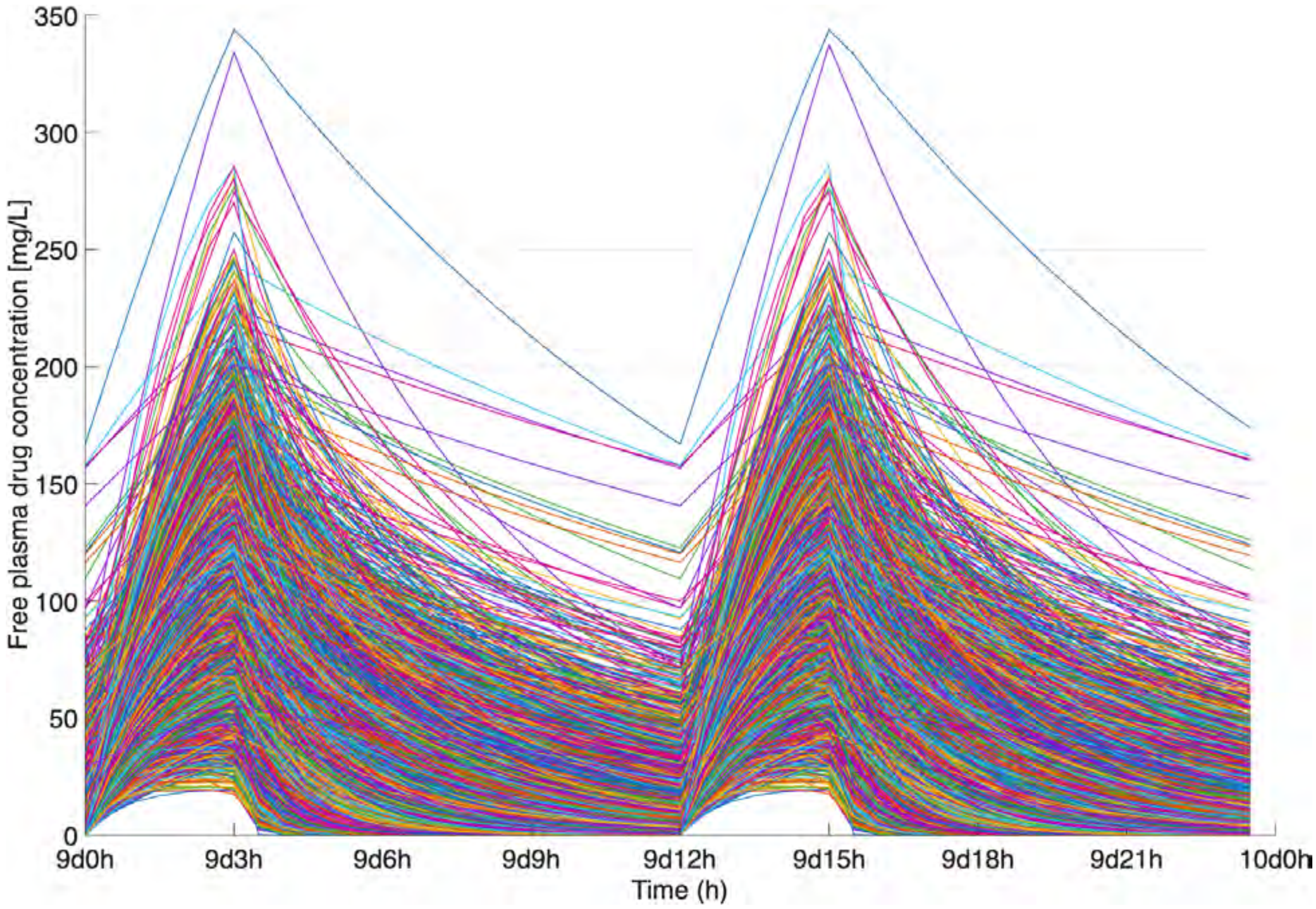

Figure S26: Simulated steady-state central-compartment concentration-time profiles for cefoxitin across the virtual-patient population receiving 4 g q12h as a 3-hour IV infusion.

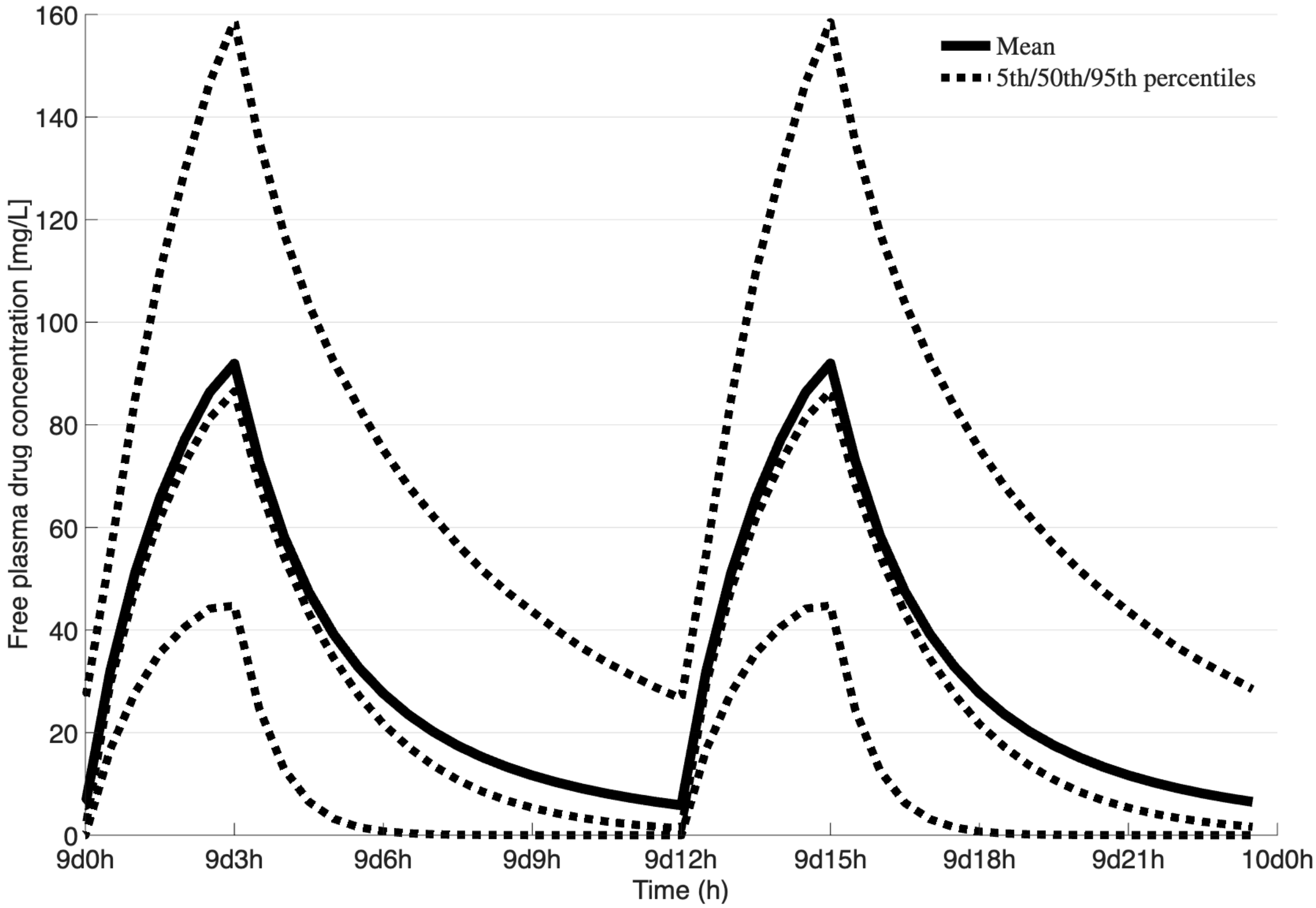

Figure S27: Simulated steady-state central-compartment concentration-time profiles for cefoxitin showing the median (50th percentile) and the 5th–95th percentile (90%) prediction interval across the virtual-patient population receiving 4 g q12h as a 3-hour IV infusion.

I Imipenem

I.1 Imipenem model parameters

Table S8: Population pharmacokinetic parameters for the two-compartment imipenem model. Values represent typical population means; IIV is expressed as percent coefficient of variation ($\%CV$).

Parameter	Mean	IIV % CV	Units
Vc_F	20.5	14.8	L
Vp_F	8.86	–	L
CL_F	8.88	17.7	L/h
Q_F	1.74	–	L/h

All parameters for the reimplementation of the imipenem model are presented in Table S8 as they appear in the model from [9]. These parameters are used in the following equations to generate the pharmacokinetic profiles seen in Figure S30 and used to calculate the AUC distribution in Figure S29.

I.2 Imipenem model equations

This model was recreated from the model proposed in [9]. The parameter values used in the patient simulations were calculated using Equation 4. The model structure consists of a central compartment (C) and a peripheral compartment (C_p), with drug administered as a 3-hour IV infusion as described by Equation 1.

$$\frac{d(C)}{dt} = \texttt{infusion rate} - \texttt{Q\_F} \cdot \frac{C}{\texttt{Vc\_F}} + \left(\texttt{Q\_F} \cdot \frac{C_p}{\texttt{Vc\_F}}\right) - \left(\texttt{CL\_F} \cdot \frac{C}{\texttt{Vc\_F}}\right) \tag{30}$$

$$\frac{d(C_p)}{dt} = \texttt{Q\_F} \cdot \frac{C}{\texttt{Vp\_F}} - \left(\texttt{Q\_F} \cdot \frac{C_p}{\texttt{Vp\_F}}\right) \tag{31}$$

I.3 Imipenem model flowchart

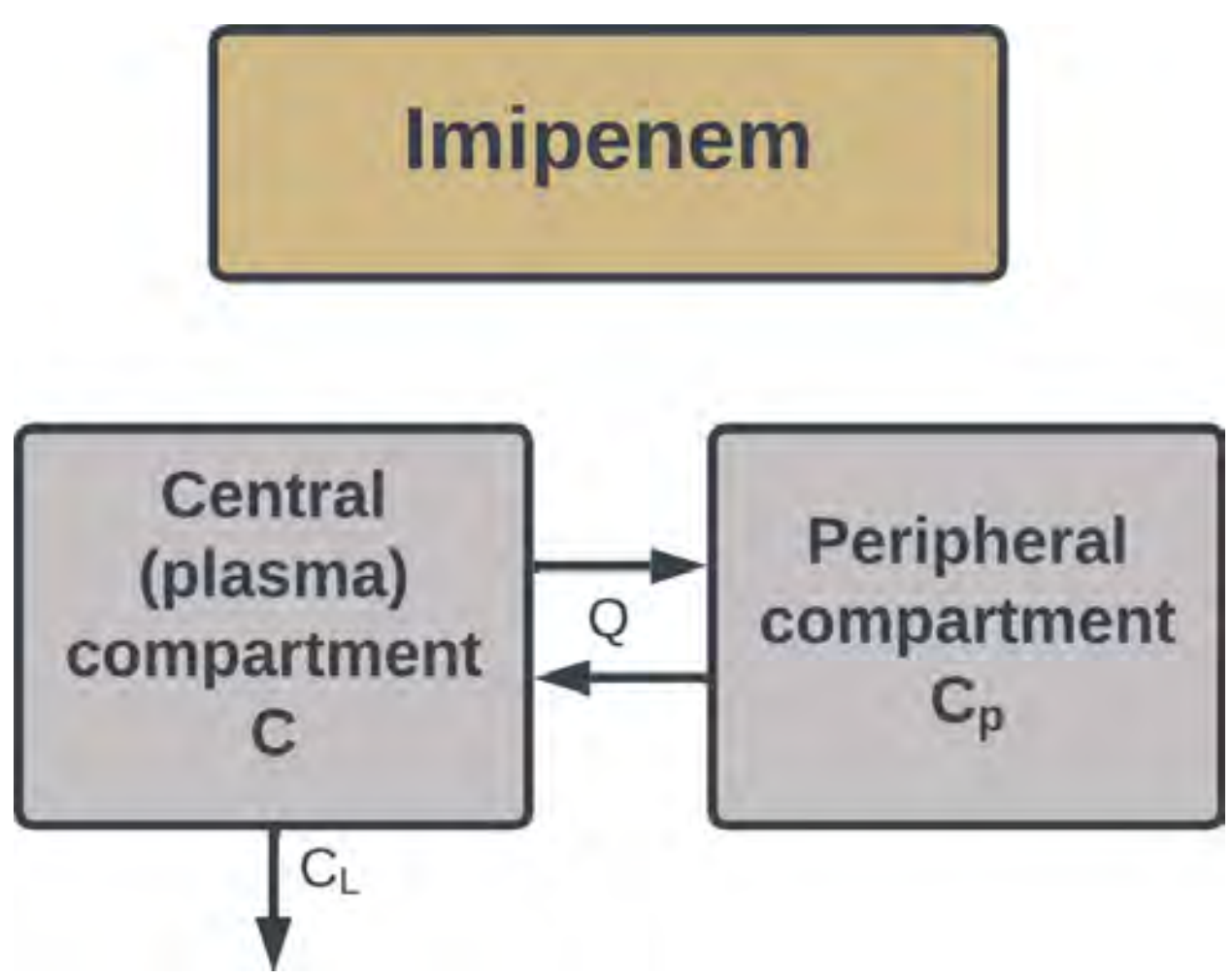

Figure S28: Schematic of the two-compartment population pharmacokinetic model for imipenem, showing the central compartment (C) and peripheral compartment (C_p), with systemic clearance (CL_F), intercompartmental clearance (Q_F), and 3-hour IV infusion input.

I.4 Imipenem model AUC distributions

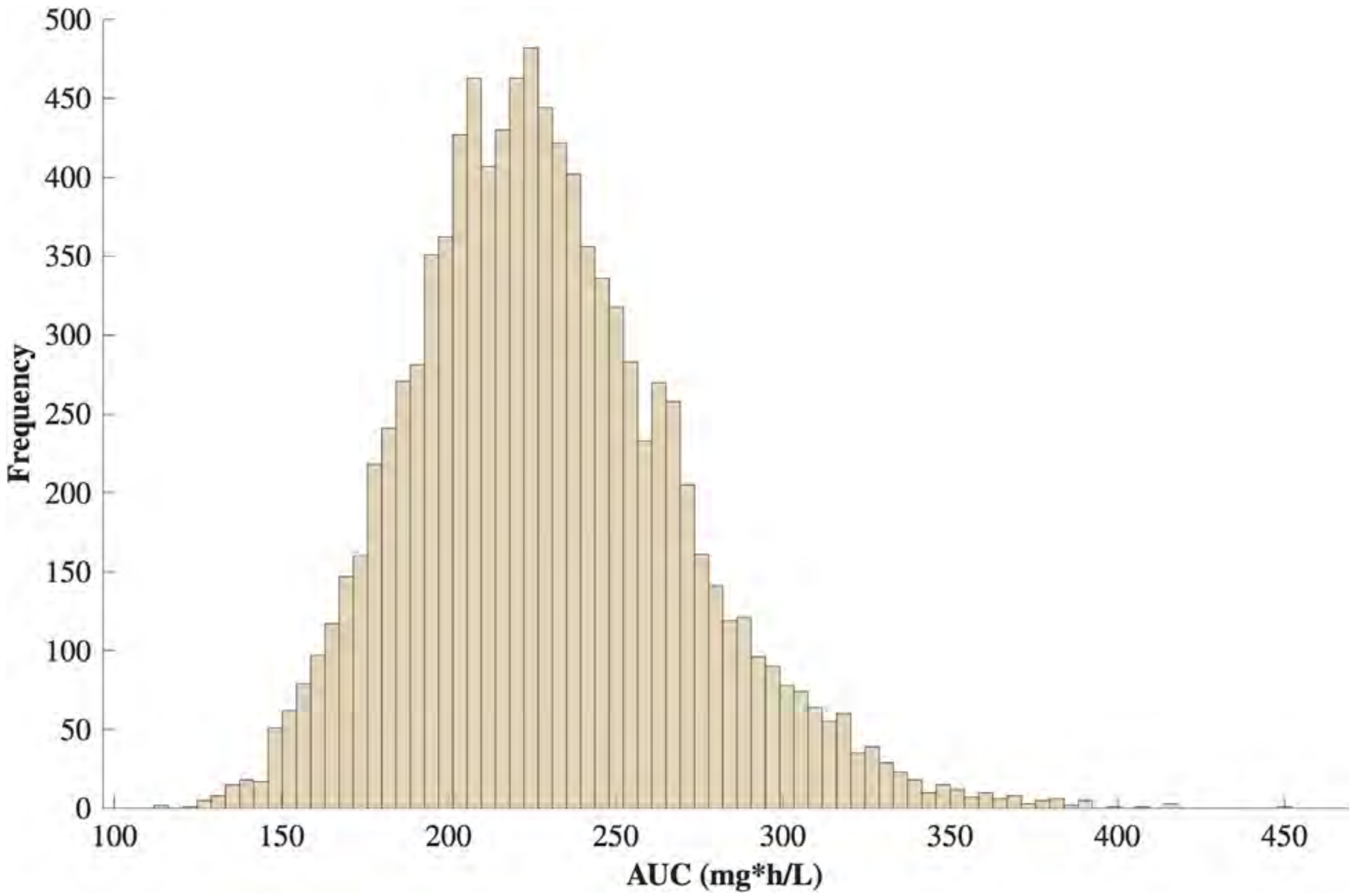

Figure S29: Steady-state AUC_{0-24} distributions across 10,000 simulated patient profiles for imipenem at 1000 mg q12h administered as a 3-hour IV infusion. Distributions reflect interindividual variability drawn from the parameter distributions in Table S8.

I.5 Imipenem model concentration time-courses

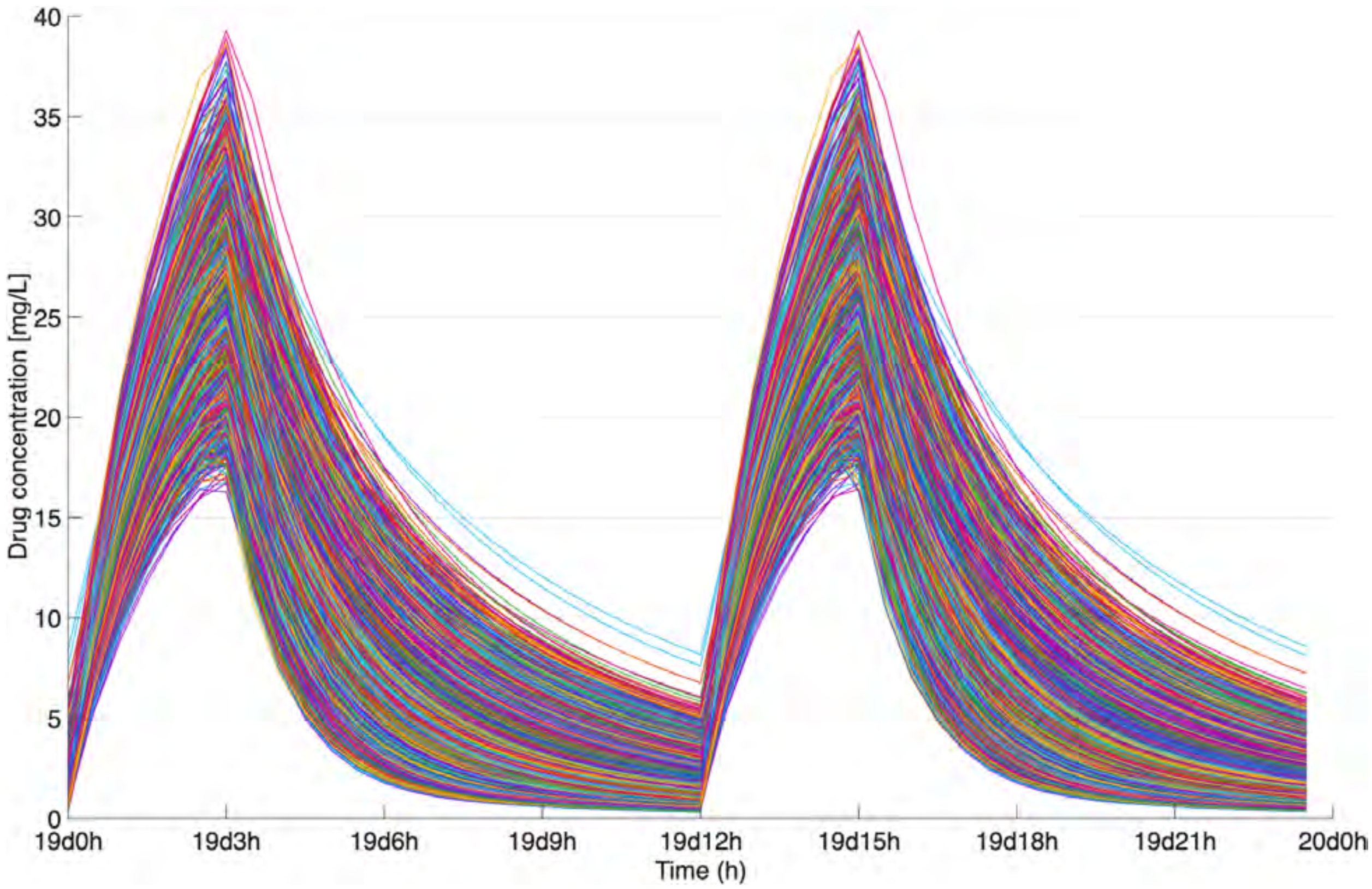

Figure S30: Simulated steady-state central compartment concentration-time profiles for imipenem across 10,000 virtual patients receiving 1000 mg q12h as a 3-hour IV infusion.

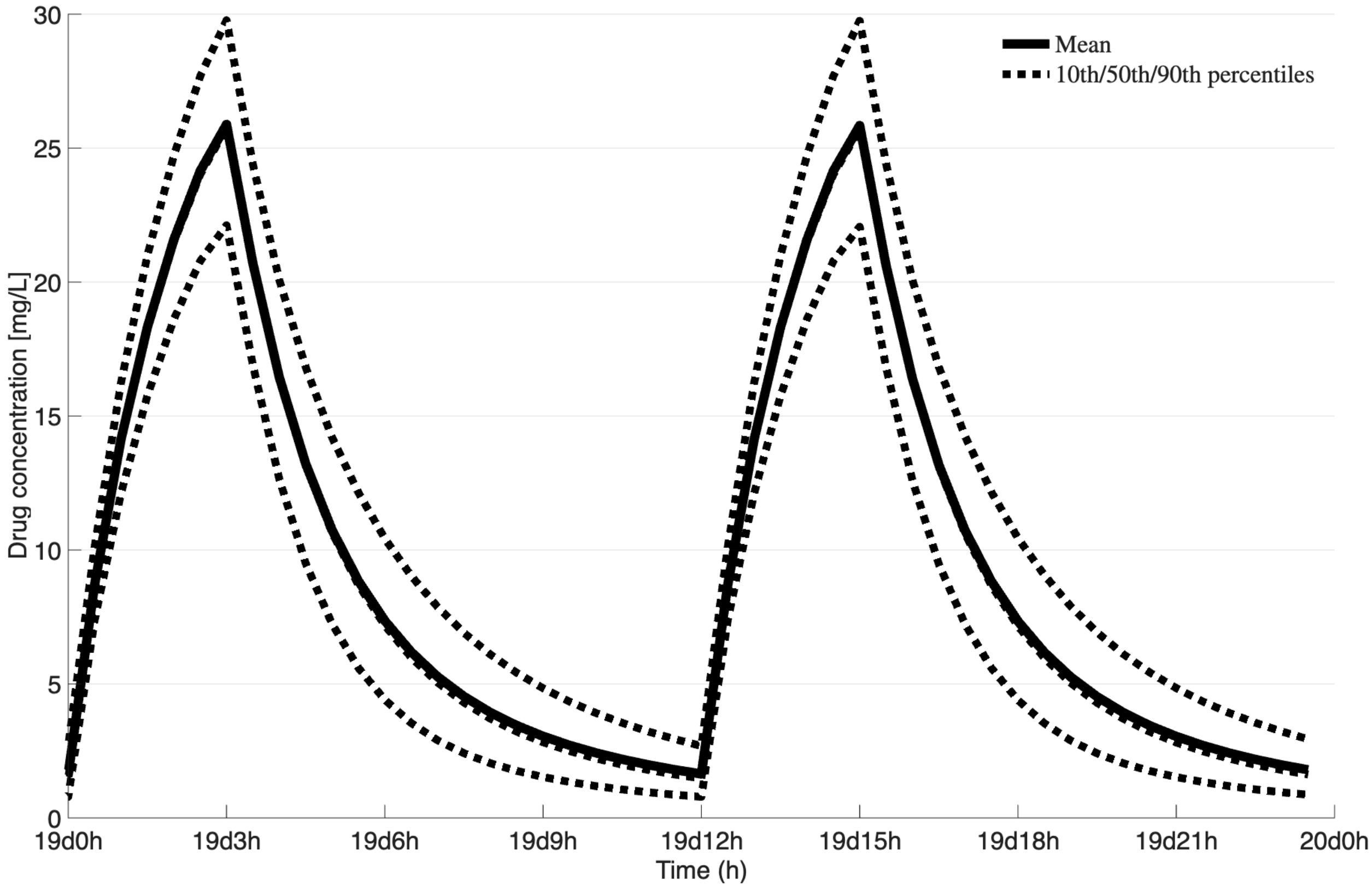

Figure S31: Simulated steady-state central compartment concentration-time profiles for imipenem showing the mean and 90% prediction interval across 10,000 virtual patients receiving 1000 mg q12h as a 3-hour IV infusion.

J Linezolid

J.1 Linezolid model parameters

Table S9: Population pharmacokinetic parameters for the transit-compartment linezolid model. Values represent typical population means; IIV and IOV are expressed as percent coefficient of variation (%CV).

Parameter	Mean	IIV % CV	IOV % CV	Units
V_C	40.2	–	–	L
MTT	0.528	–	56.8	h
n	5	–	–	–
K_a	1.22	–	78.5	1/h
F	1	–	22.0	–
CL	3.57	37.1	–	L/h

V_C is volume in central compartment; MTT is the absorption mean transit time; n is the number of transit compartments; K_a is the absorption rate constant; F is the bioavailability; CL is the clearance.

J.2 Linezolid model equations

This model was recreated from the model proposed in [3]. The parameter values used in the patient simulations were calculated using Equation 4. The model structure was an eight (8) compartment model with an absorption compartment (q_0), five (5) transit compartments (q_1–q_5), a final pre-absorption compartment (q), and a central compartment (C).

$$\frac{d(q_0)}{dt} = -\texttt{k\_tr} \cdot q_0 \tag{32}$$

$$\frac{d(q_i)}{dt} = \texttt{k\_tr}\Big(q_{i-1} - q_i\Big), \quad i = 1, \ldots, 5 \tag{33}$$

$$\frac{d(q)}{dt} = \texttt{k\_tr} \cdot q_5 - \texttt{K\_a} \cdot q \tag{34}$$

$$\frac{d(C)}{dt} = \texttt{F} \cdot \texttt{K\_a} \cdot q - C \cdot \left(\frac{\texttt{CL}}{\texttt{V\_C}}\right) \tag{35}$$

J.3 Linezolid model flowchart

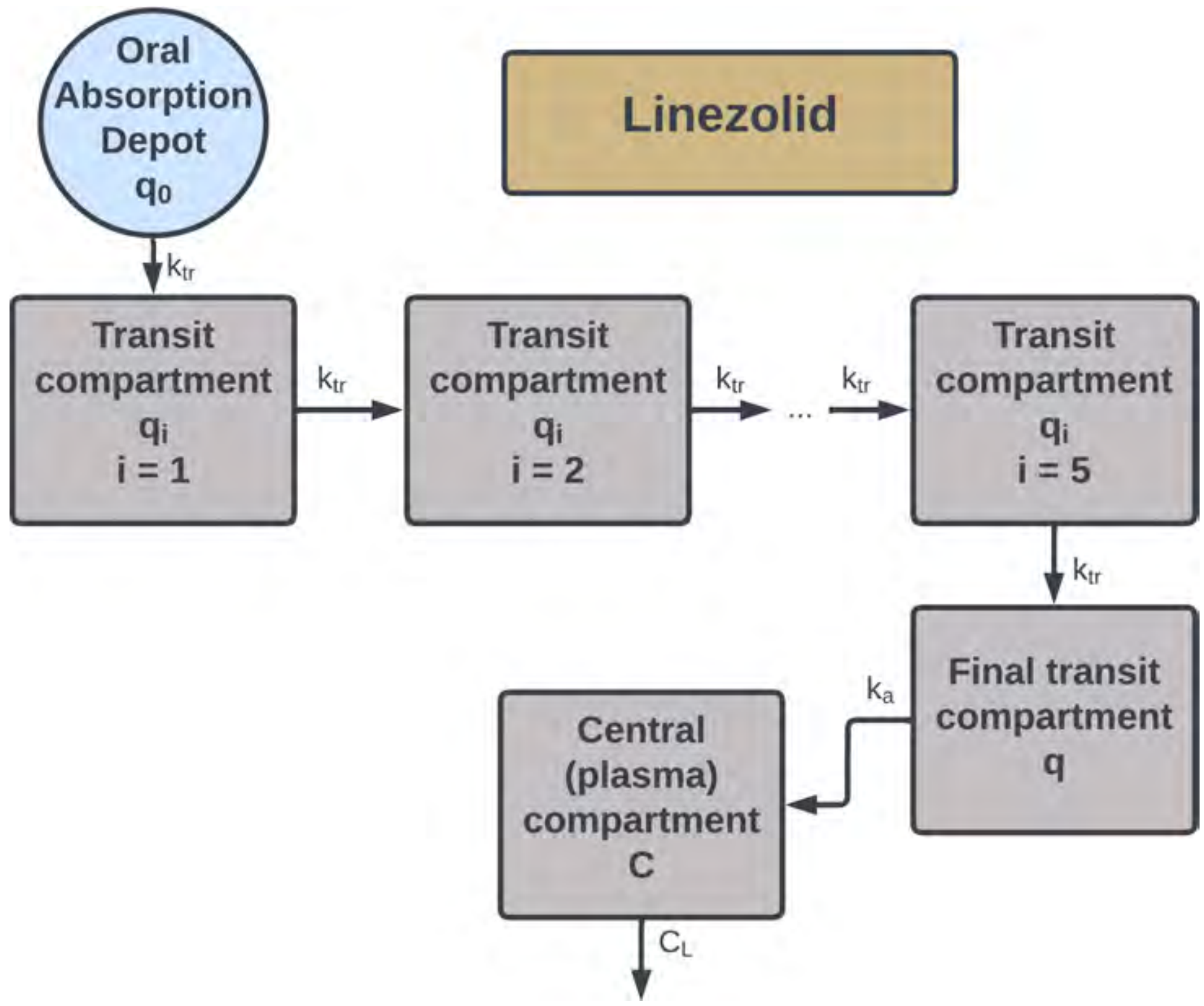

Figure S32: Schematic of the eight-compartment population pharmacokinetic model for linezolid, showing the absorption compartment (q_0), five transit compartments (q_1–q_5), final pre-absorption compartment (q), and central compartment (C), with absorption rate constant (`K_a`), transit rate constant (`k_tr`), bioavailability (`F`), and systemic clearance (`CL`).

J.4 Linezolid model AUC distributions

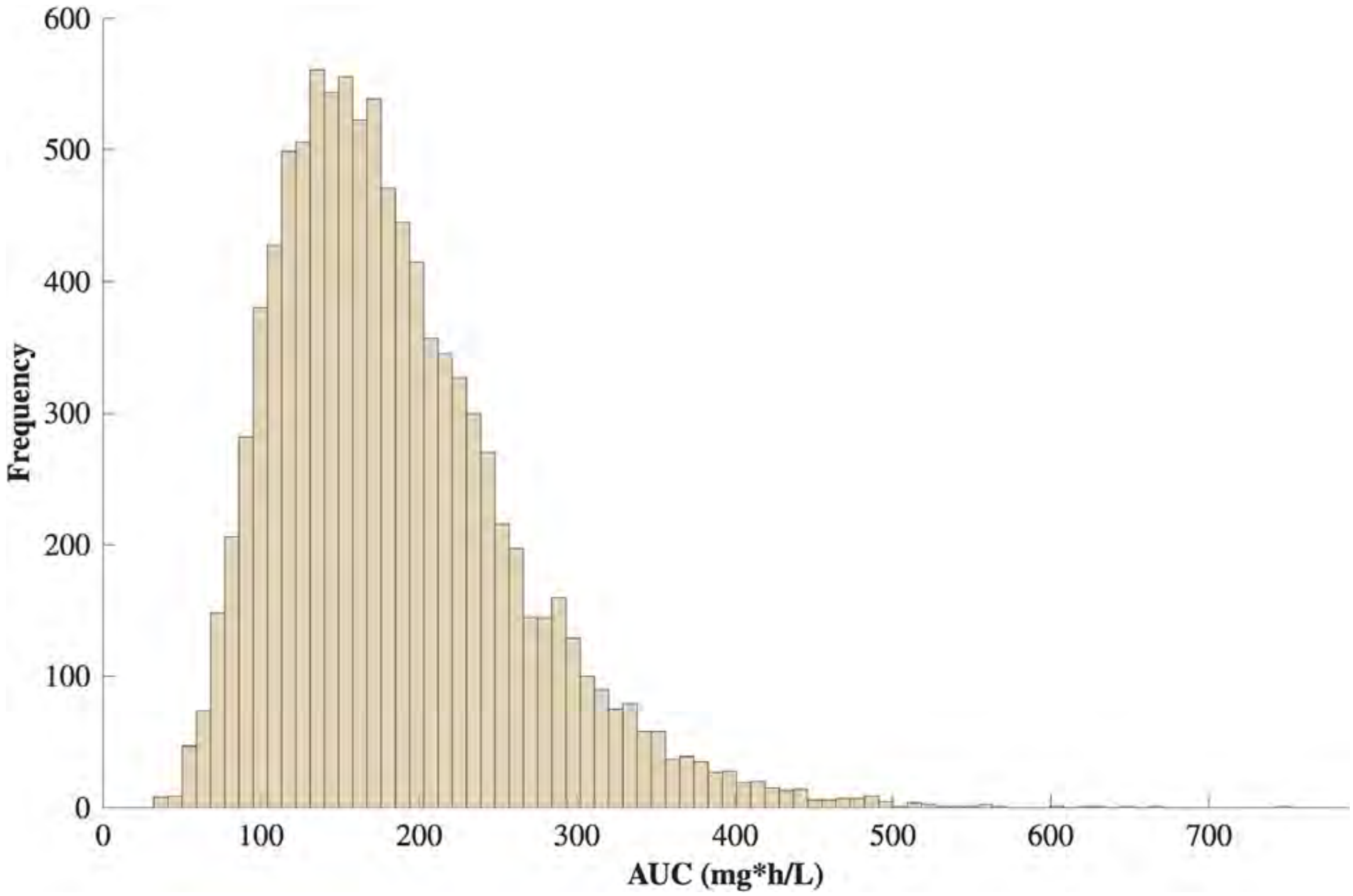

Figure S33: Steady-state AUC_{0-24} distributions across 10,000 simulated patient profiles for linezolid at 600 mg q24h. Distributions reflect interindividual variability drawn from the parameter distributions in Table S9.

J.5 Linezolid model concentration time-courses

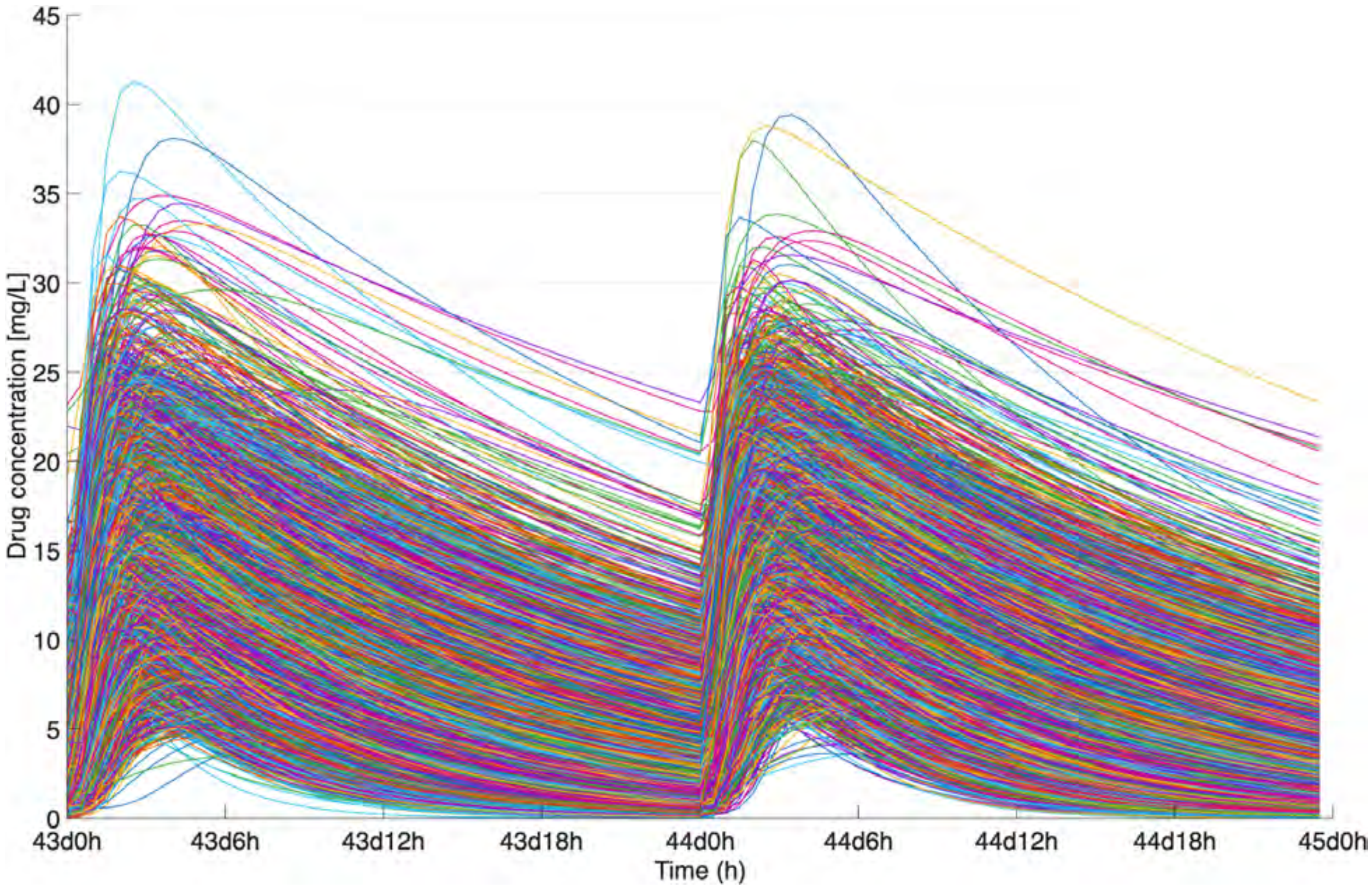

Figure S34: Simulated steady-state central compartment concentration-time profiles for linezolid across 10,000 virtual patients receiving 600 mg q24h.

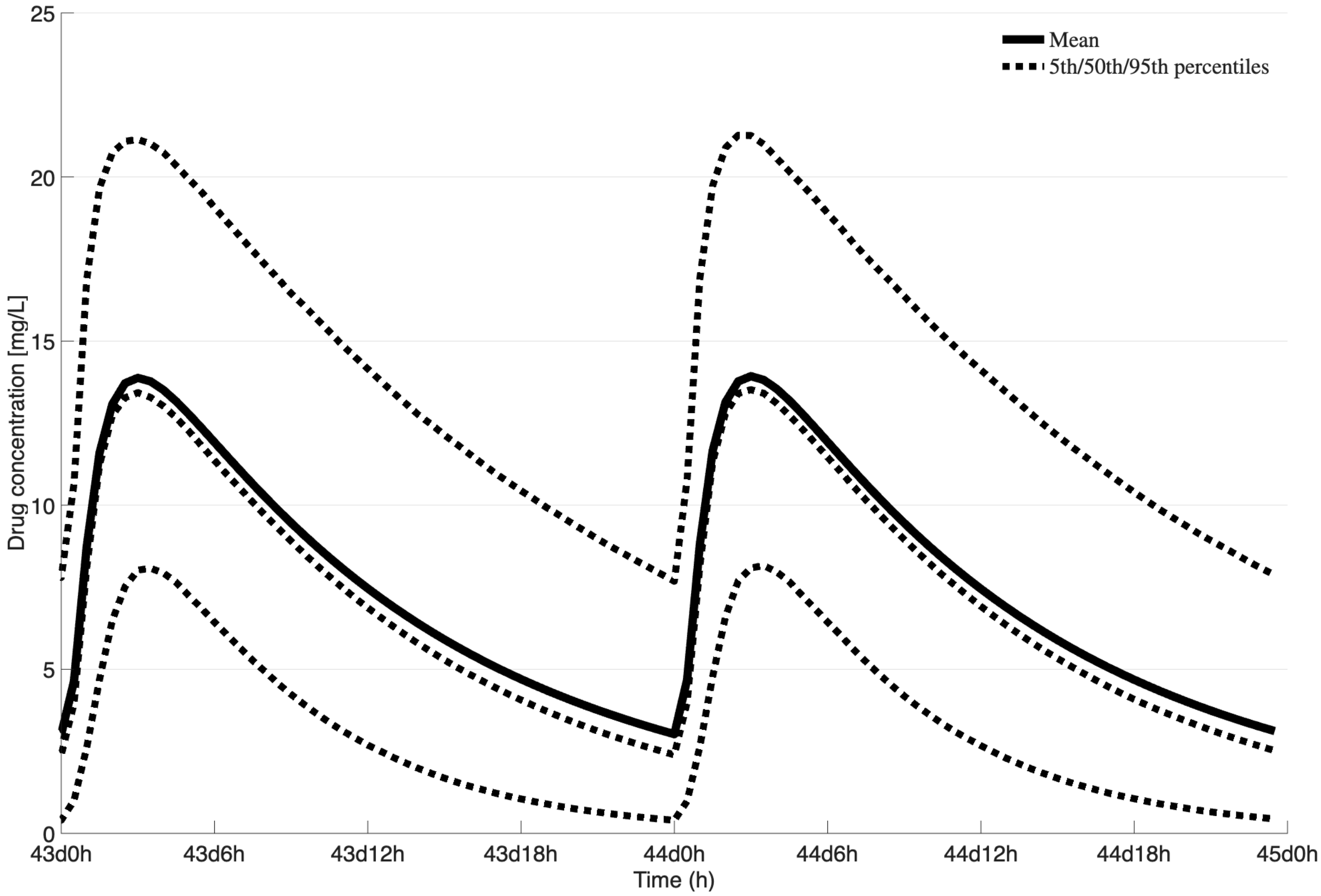

Figure S35: Simulated steady-state central compartment concentration-time profiles for linezolid showing the mean and 90% prediction interval across 10,000 virtual patients receiving 600 mg q24h.

K Moxifloxacin

K.1 Moxifloxacin model parameters

Table S10: Population pharmacokinetic parameters for the one-compartment moxifloxacin model. Values represent typical population means; IIV is expressed as percent coefficient of variation ($\%CV$).

Parameter	Mean	IIV % CV	Units
V_F	110	38	L
CL_F	9.59	30	L/h
ka	2.69	196	1/h

All parameters used for the reimplementation of the moxifloxacin model are presented in Table S10 as they appear in the model from [38]. These parameters are used in the following equations to generate the pharmacokinetic profiles seen in Figure S38 and used to calculate the AUC distributions in Figure S37.

K.2 Moxifloxacin model equations

This model was recreated from the model proposed in [38]. The parameter values used in the patient simulations were calculated using Equation 4. The model structure consists of an absorption compartment (C_q) and a central compartment (C).

$$\frac{d(C_q)}{dt} = -\texttt{ka} \cdot C_q \tag{36}$$

$$\frac{d(C)}{dt} = \texttt{ka} \cdot C_q - \left(\texttt{CL\_F} \cdot \frac{C}{\texttt{V\_F}}\right) \tag{37}$$

K.3 Moxifloxacin model flowchart

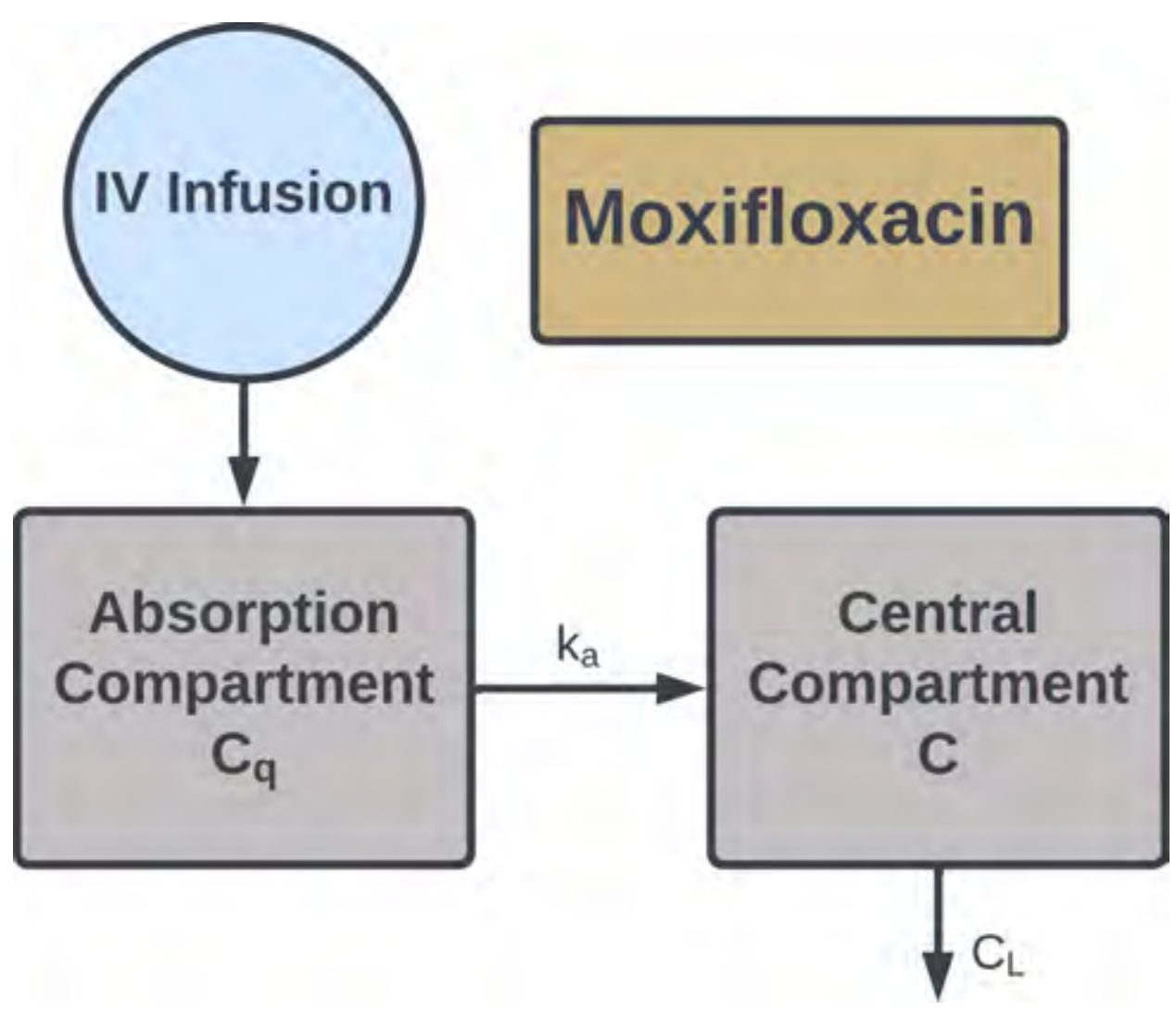

Figure S36: Schematic of the one-compartment population pharmacokinetic model for moxifloxacin, showing the absorption compartment (C_q) and central compartment (C), with first-order absorption rate constant (ka) and systemic clearance (CL_F).

K.4 Moxifloxacin model AUC distributions

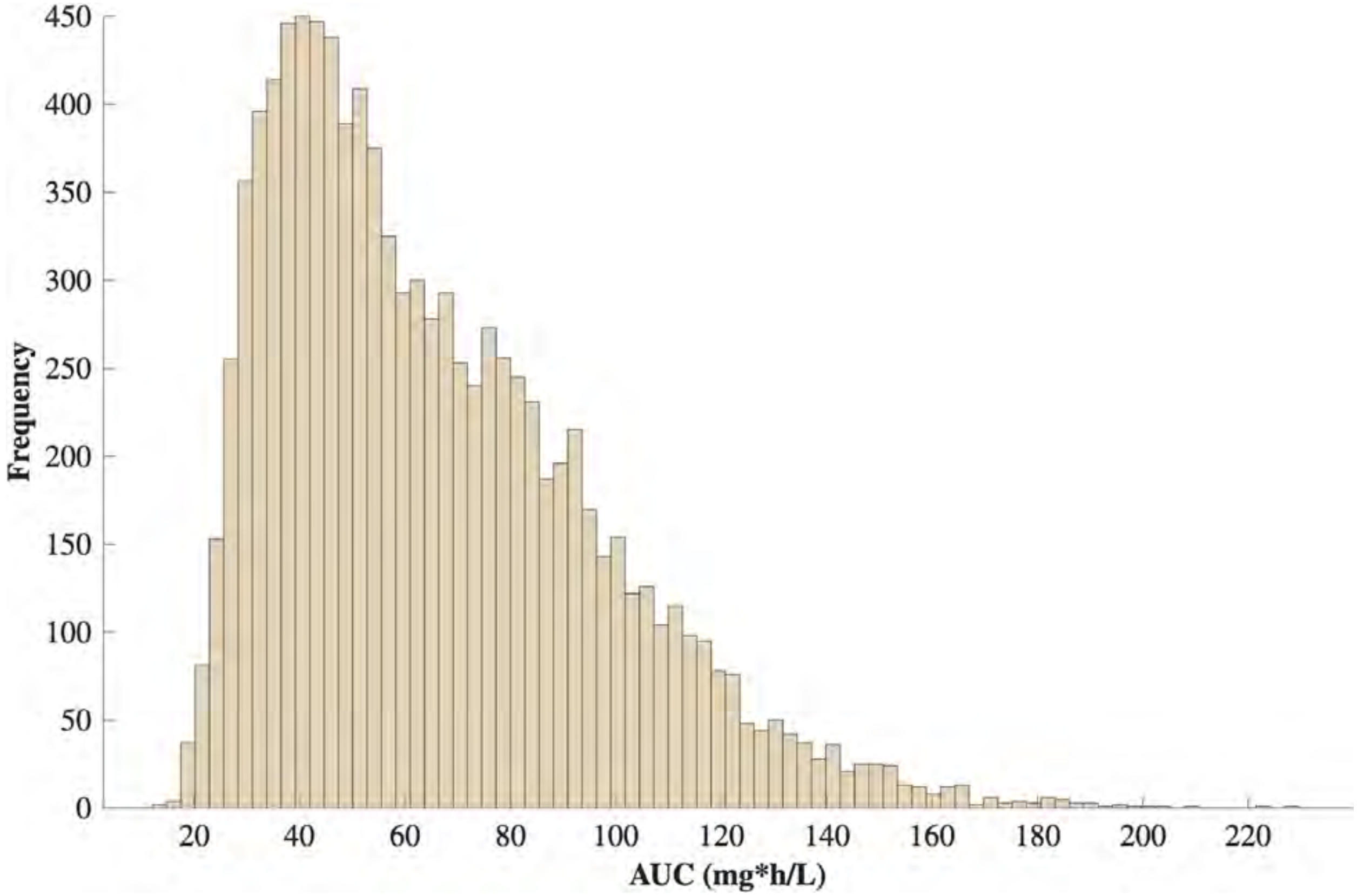

Figure S37: Steady-state AUC_{0-24} distributions across 10,000 simulated patient profiles for moxifloxacin at 400 mg q24h and 800 mg q24h. Distributions reflect interindividual variability drawn from the parameter distributions in Table S10.

K.5 Moxifloxacin model concentration time-courses

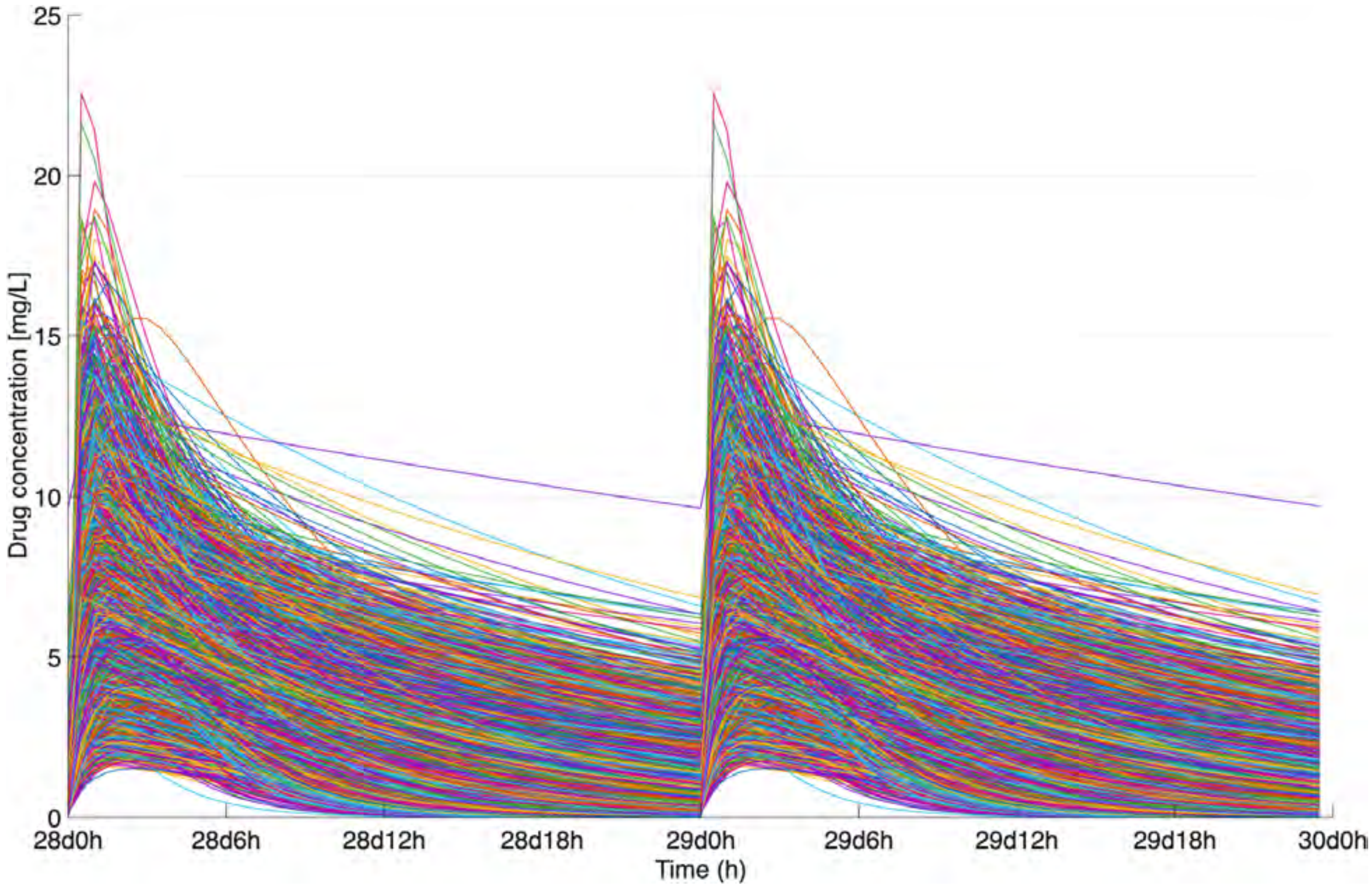

Figure S38: Simulated steady-state central compartment concentration-time profiles for moxifloxacin across 10,000 virtual patients receiving 400 mg q24h and 800 mg q24h.

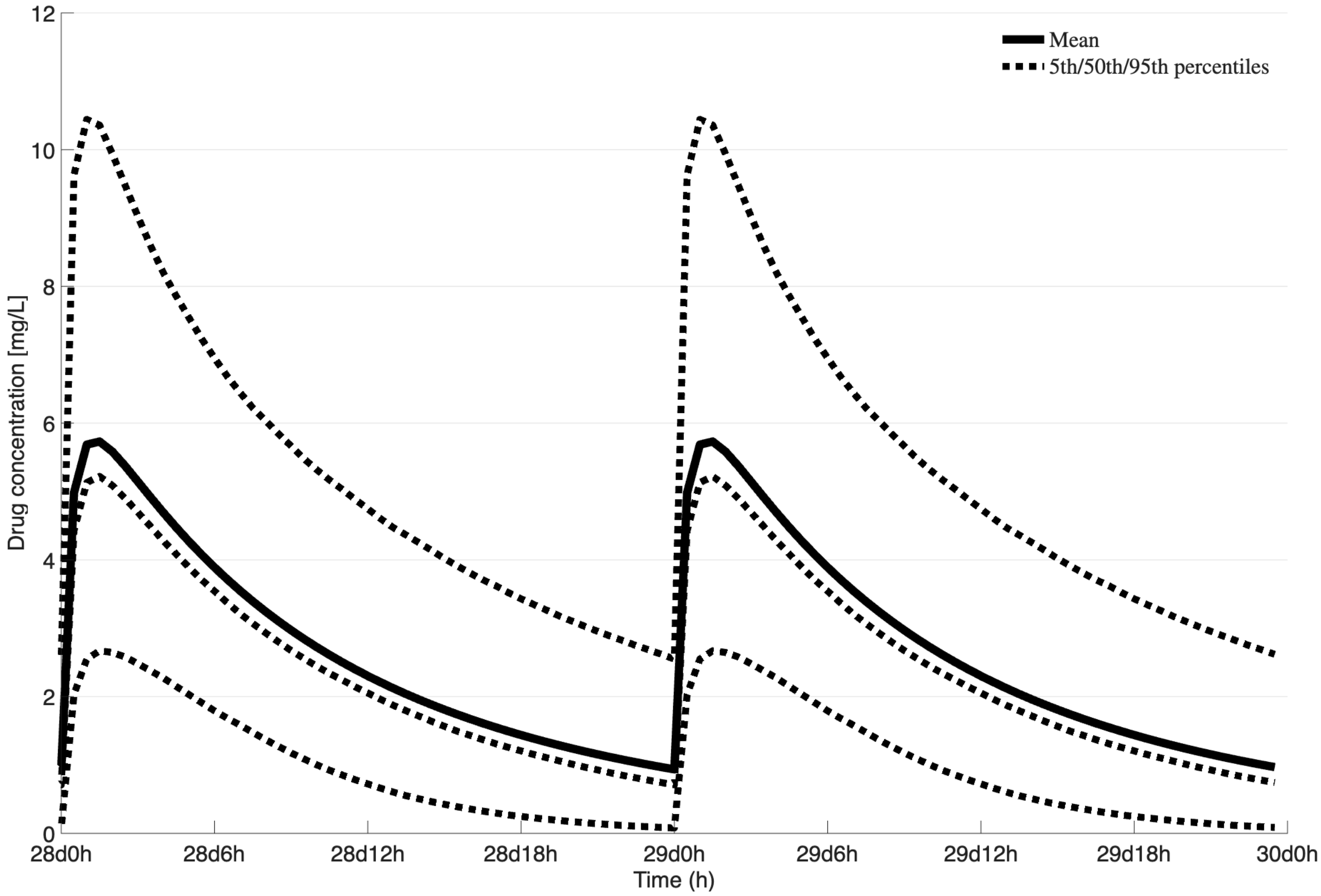

Figure S39: Simulated steady-state central compartment concentration-time profiles for moxifloxacin showing the mean and 90% prediction interval across 10,000 virtual patients receiving 400 mg q24h and 800 mg q24h.

L Rifabutin

L.1 Rifabutin model parameters

Table S11: Population pharmacokinetic parameters for the transit-compartment rifabutin model with parallel des-rifabutin metabolite tracking. Values represent typical population means; IIV is expressed as percent coefficient of variation ($\%CV$).

Parameter	Mean	IIV % CV	Units
MTT	2.16	0.518	h
n	7.15	–	–
k_tr	3.77	–	/h
k_a	0.25	0.310	/h
F_m	0.09	0.893	–
CL_1	58.80	0.540	L/h
CL_2	122.00	0.783	L/h
Q_1	62.21	0.602	L/h
Q_2	71.80	0.608	L/h
Q_3	5.76	0.515	L/h
Vd_C,RFB	6.55	1.691	L
Vd_C,dRFB	37.30	1.114	L
Vd_P,RFB	1580	0.656	L
Vd_P,dRFB	1220	0.268	L

MTT, mean transit time through transit compartments; n, number of transit compartments; k_tr, transit rate constant; k_a, absorption rate constant from the absorption compartment to the central compartment; F_m, first-pass metabolism fraction of rifabutin converted to des-rifabutin; CL_1, clearance rate of rifabutin from the central compartment; CL_2, clearance rate of des-rifabutin from the central compartment; Q_1, intercompartmental clearance between central and peripheral compartments for rifabutin; Q_2, intercompartmental clearance between central and peripheral compartments for des-rifabutin; Q_3, conversion clearance from rifabutin to des-rifabutin in the central compartment; Vd_C,RFB, volume of distribution for rifabutin in the central compartment; Vd_C,dRFB, volume of distribution for des-rifabutin in the central compartment; Vd_P,RFB, volume of distribution for rifabutin in the peripheral compartment; Vd_P,dRFB, volume of distribution for des-rifabutin in the peripheral compartment.

L.2 Rifabutin model equations

This model was recreated from the model proposed in [16]. The parameter values used in the patient simulations were calculated using Equation 4. The Stirling approximation for the gamma function is given by Equation 2 from the introduction. The model structure consists of a transit-compartment absorption input (m_{abs}), a rifabutin central compartment ($C_{C,RFB}$), a rifabutin peripheral compartment ($C_{P,RFB}$), a des-rifabutin central compartment ($C_{C,dRFB}$), and a des-rifabutin peripheral compartment ($C_{P,dRFB}$), with first-pass metabolism generating the des-rifabutin metabolite from both absorption and central compartment conversion.

$$\frac{d(m_{abs})}{dt} = \texttt{dose} \cdot \texttt{k\_tr} \cdot \frac{(\texttt{k\_tr} \cdot t)^n \cdot \exp(-\texttt{k\_tr} \cdot t)}{\Gamma_n} - \texttt{k\_a} \cdot m_{abs} \tag{38}$$

$$\frac{d(C_{C,RFB})}{dt} = \frac{\texttt{k\_a} \cdot (1 - \texttt{F\_m})}{\texttt{Vd\_C,RFB}} \cdot m_{abs} + \frac{-\texttt{Q\_1} - \texttt{CL\_1} - \texttt{Q\_3}}{\texttt{Vd\_C,RFB}} \cdot C_{C,RFB} + \frac{\texttt{Q\_1}}{\texttt{Vd\_C,RFB}} \cdot C_{P,RFB} \tag{39}$$

$$\frac{d(C_{C,dRFB})}{dt} = \frac{\texttt{k\_a} \cdot \texttt{F\_m}}{\texttt{Vd\_C,dRFB}} \cdot m_{abs} + \frac{\texttt{Q\_3}}{\texttt{Vd\_C,dRFB}} \cdot C_{C,RFB} + \frac{-\texttt{Q\_2} - \texttt{CL\_2}}{\texttt{Vd\_C,dRFB}} \cdot C_{C,dRFB} + \frac{\texttt{Q\_2}}{\texttt{Vd\_C,dRFB}} \cdot C_{P,dRFB} \tag{40}$$

$$\frac{d(C_{P,RFB})}{dt} = \frac{\texttt{Q\_1}}{\texttt{Vd\_P,RFB}} \cdot C_{C,RFB} - \frac{\texttt{Q\_1}}{\texttt{Vd\_P,RFB}} \cdot C_{P,RFB} \tag{41}$$

$$\frac{d(C_{P,dRFB})}{dt} = \frac{\texttt{Q\_2}}{\texttt{Vd\_P,dRFB}} \cdot C_{C,dRFB} - \frac{\texttt{Q\_2}}{\texttt{Vd\_P,dRFB}} \cdot C_{P,dRFB} \tag{42}$$

L.3 Rifabutin model flowchart

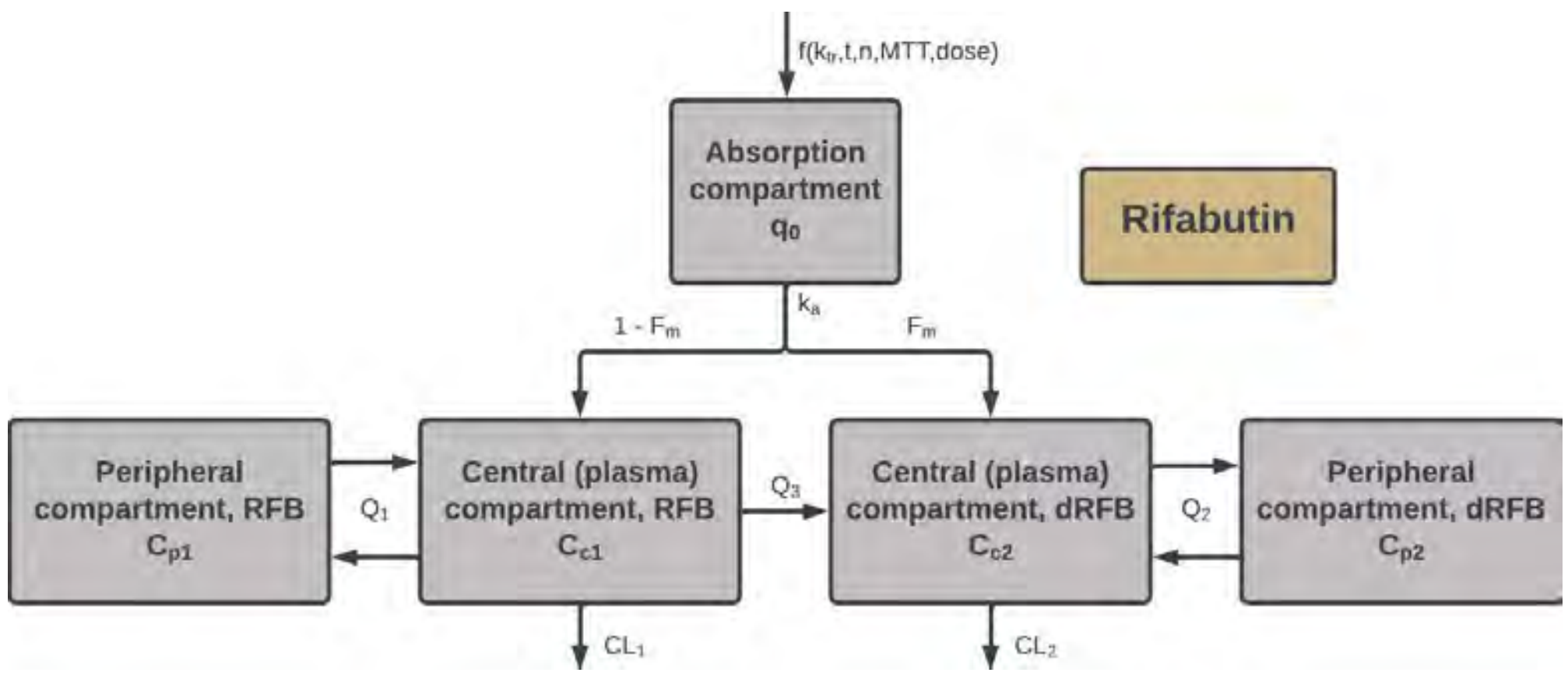

Figure S40: Schematic of the population pharmacokinetic model for rifabutin, showing the transit-compartment absorption input (m_{abs}), rifabutin central compartment ($C_{C,RFB}$), rifabutin peripheral compartment ($C_{P,RFB}$), des-rifabutin central compartment ($C_{C,dRFB}$), and des-rifabutin peripheral compartment ($C_{P,dRFB}$), with first-pass metabolism fraction (`F_m`), absorption rate constant (`k_a`), systemic clearances (`CL_1`, `CL_2`), intercompartmental clearances (`Q_1`, `Q_2`), and central compartment conversion clearance (`Q_3`).

L.4 Rifabutin model AUC distributions

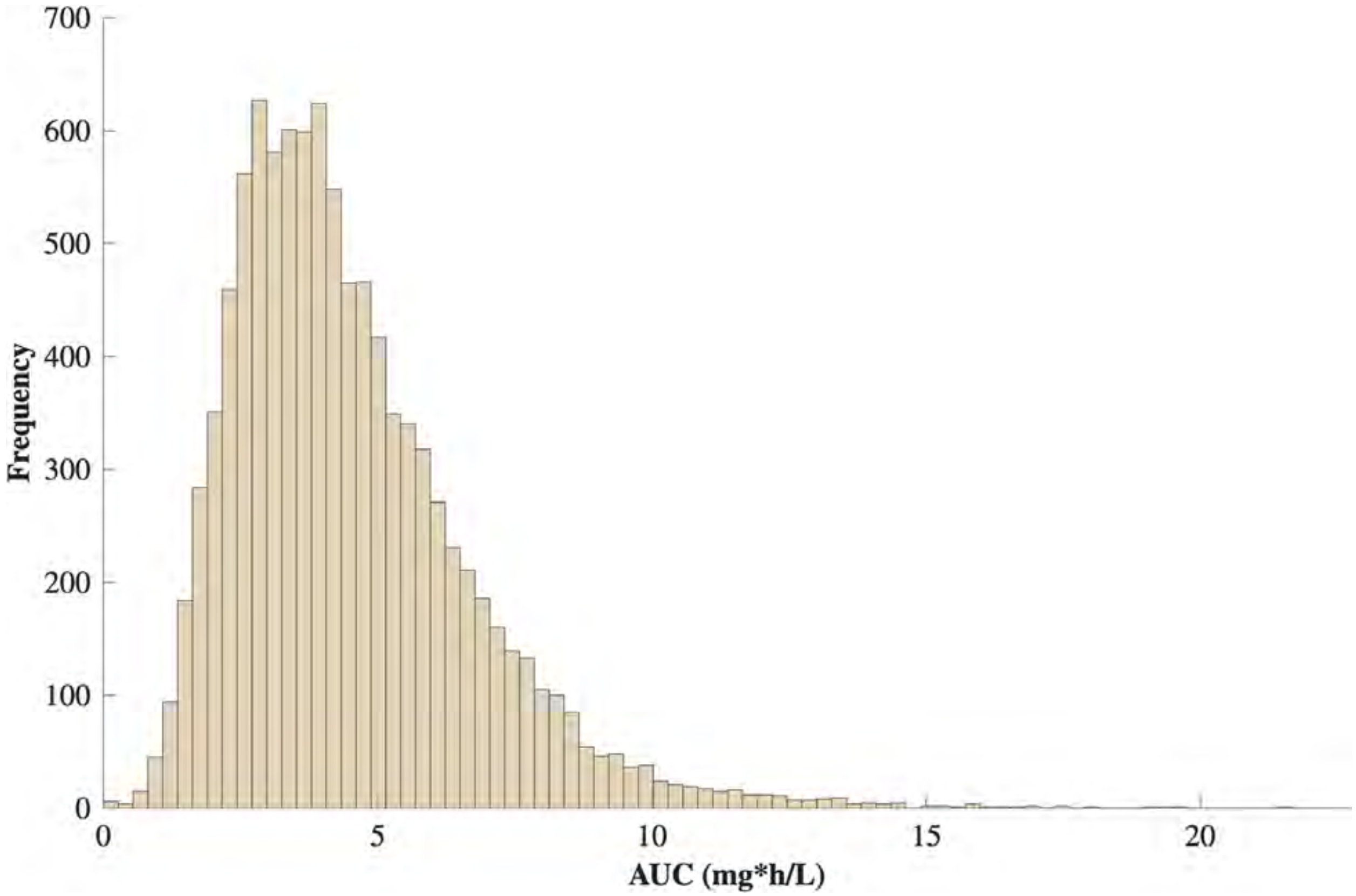

Figure S41: Steady-state AUC_{0-24} distributions across 10,000 simulated patient profiles for rifabutin at 300 mg q24h. Distributions reflect interindividual variability drawn from the parameter distributions in Table S11.

L.5 Rifabutin model concentration time-courses

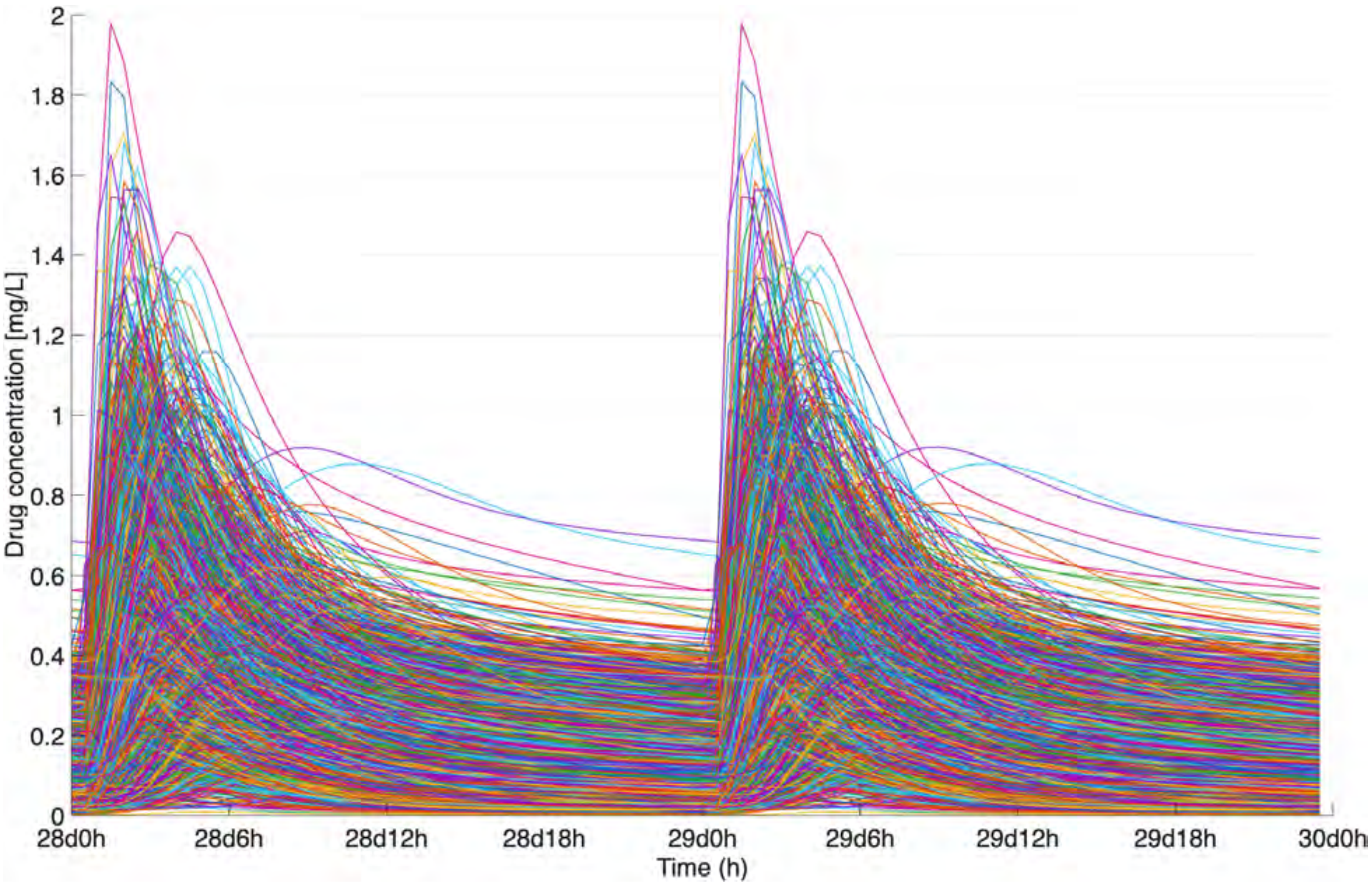

Figure S42: Simulated steady-state central compartment concentration-time profiles for rifabutin across 10,000 virtual patients receiving 300 mg q24h.

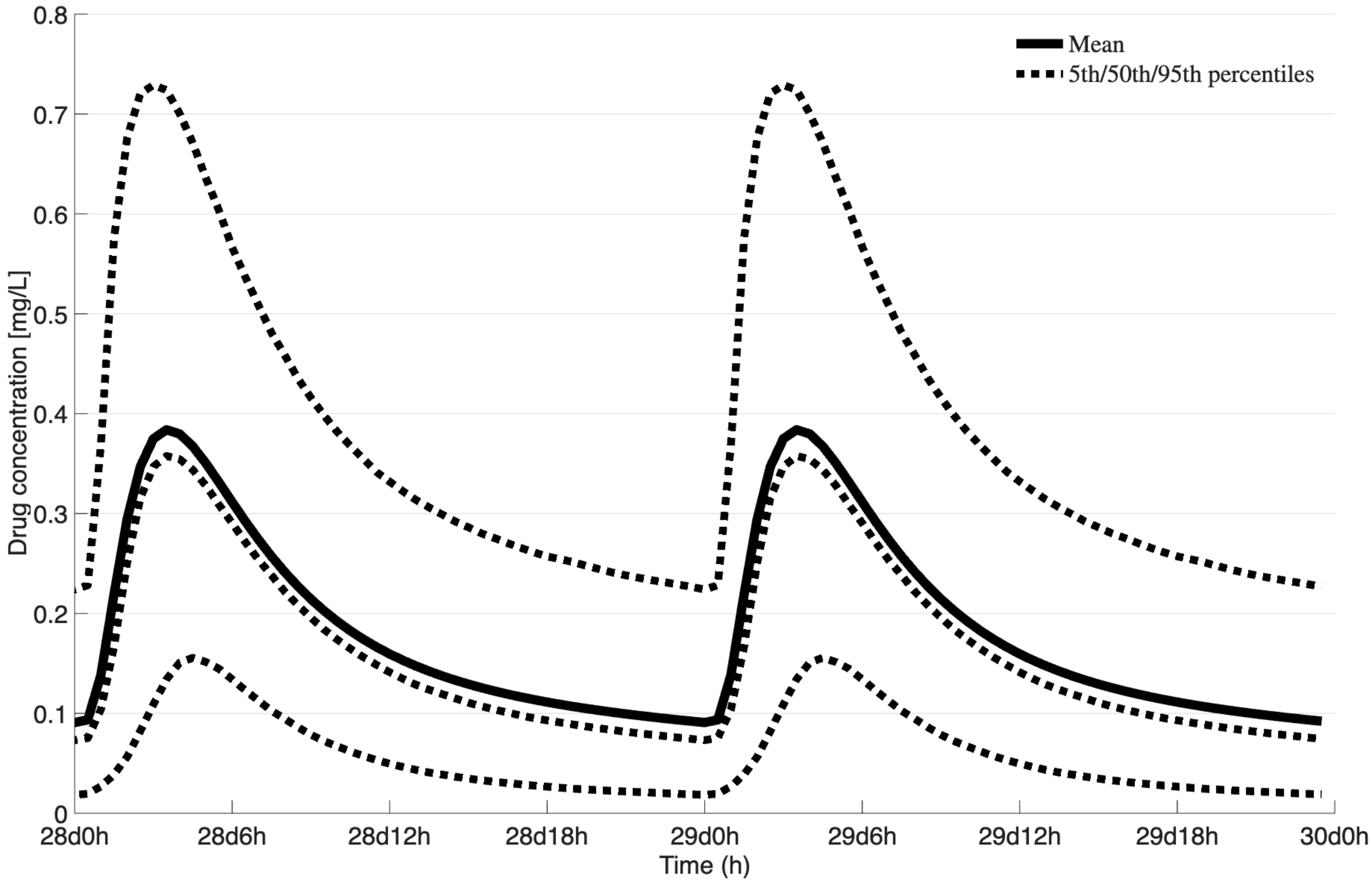

Figure S43: Simulated steady-state central compartment concentration-time profiles for rifabutin showing the mean and 90% prediction interval across 10,000 virtual patients receiving 300 mg q24h.

M Rifampin

M.1 Rifampin model parameters

Table S12: Population pharmacokinetic parameters for the transit-compartment rifampin model. Values represent typical population means; θ denotes the dose-type effect coefficient, η denotes the interindividual variability variance, and κ denotes the interoccasional variability variance.

Parameter	Mean	θ	η	κ	Units
CL/F	19.2	0.236	0.279	0.0508	L/hr
V/F	53.2	-	0.188	-	L/hr
k_a	1.15	-	0.439	-	1/hr
MTT	0.424	1.04	0.361	0.461	hr
n	7.13	-	2.44	-	-

`CL/F` represents clearance rate constant; `V/F` is the volume of distribution; `K_a` is the absorption rate constant; `MTT` is the mean transit time between `n` compartments; `n` is the number of theoretical transit compartments; `F` is bioavailability.

The parameters for this RIF model were calculated using the general form below.

$$\texttt{CL\_pt} = \texttt{CL\_mean} \cdot \Big(1 + \theta_{CL/F} \cdot \texttt{dose\_type}\Big) \cdot \Big(\exp(\texttt{random}\big(\texttt{`Normal'}, 0, \sqrt{\eta}\big)\Big) \tag{43}$$

Where the parameters listed in Table S12 that have θ values include that aspect of Equation 43 and parameters that have η values in Table S12 include that aspect of Equation 43. The η part of Equation 43 employs an exponential value from a randomly distributed normal distribution centered around 0 with standard deviation of η. κ values are used in the same manner as η values, but for interoccasional variances. An example of this is shown in Equation 44

$$\texttt{CL\_pt} = \texttt{CL\_pt} \cdot \exp(\texttt{random}(\texttt{`Normal'}, 0, \sqrt{\kappa})) \tag{44}$$

M.2 Rifampin model equations

The Rifampin population PK model was re-implemented from [46]. Briefly, the two compartment model contains an absorption compartment (q_0) (Equation 47) and a central compartment (C) (Equation 48). The model also accounts for n transit compartments.

$$\texttt{k\_tr} = \frac{\texttt{n} + 1}{\texttt{MTT}} \tag{45}$$

$$\texttt{q0\_frac} = \Big(\texttt{k\_tr} \cdot t\Big)^{\texttt{n}} \cdot \Big(\exp\left(-\texttt{ktr} \cdot t\right)\Big) / \Gamma_{\texttt{n}} \tag{46}$$

$$\frac{d(q_0)}{dt} = \texttt{dose} \cdot \texttt{F} \cdot \texttt{k\_tr} \cdot \texttt{q0\_frac} - \texttt{k\_a} \cdot (q_0) \tag{47}$$

$$\frac{d(C)}{dt} = \texttt{k\_a} \cdot (q_0) - \left(\frac{\texttt{CL\_F}}{\texttt{V\_F}}\right) \cdot (C) \tag{48}$$

M.3 Rifampin model flowchart

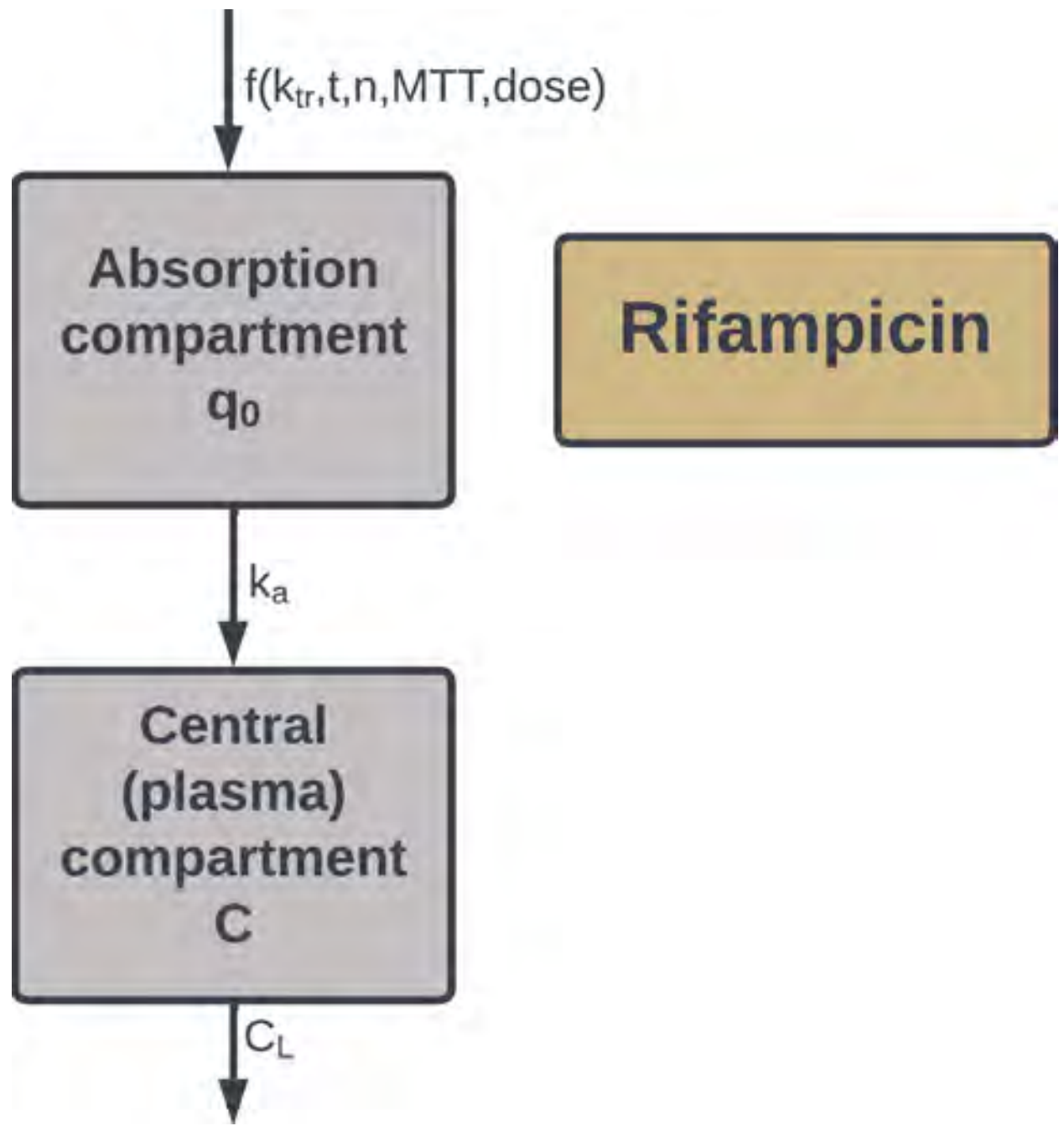

Figure S44: Schematic of the one-compartment population pharmacokinetic model for rifampin with transit-compartment absorption, showing the absorption compartment (q_0) and central compartment (C), with absorption rate constant (`k_a`), transit rate constant (`k_tr`), bioavailability (`F`), and systemic clearance (`CL/F`).

M.4 Rifampin model AUC distributions

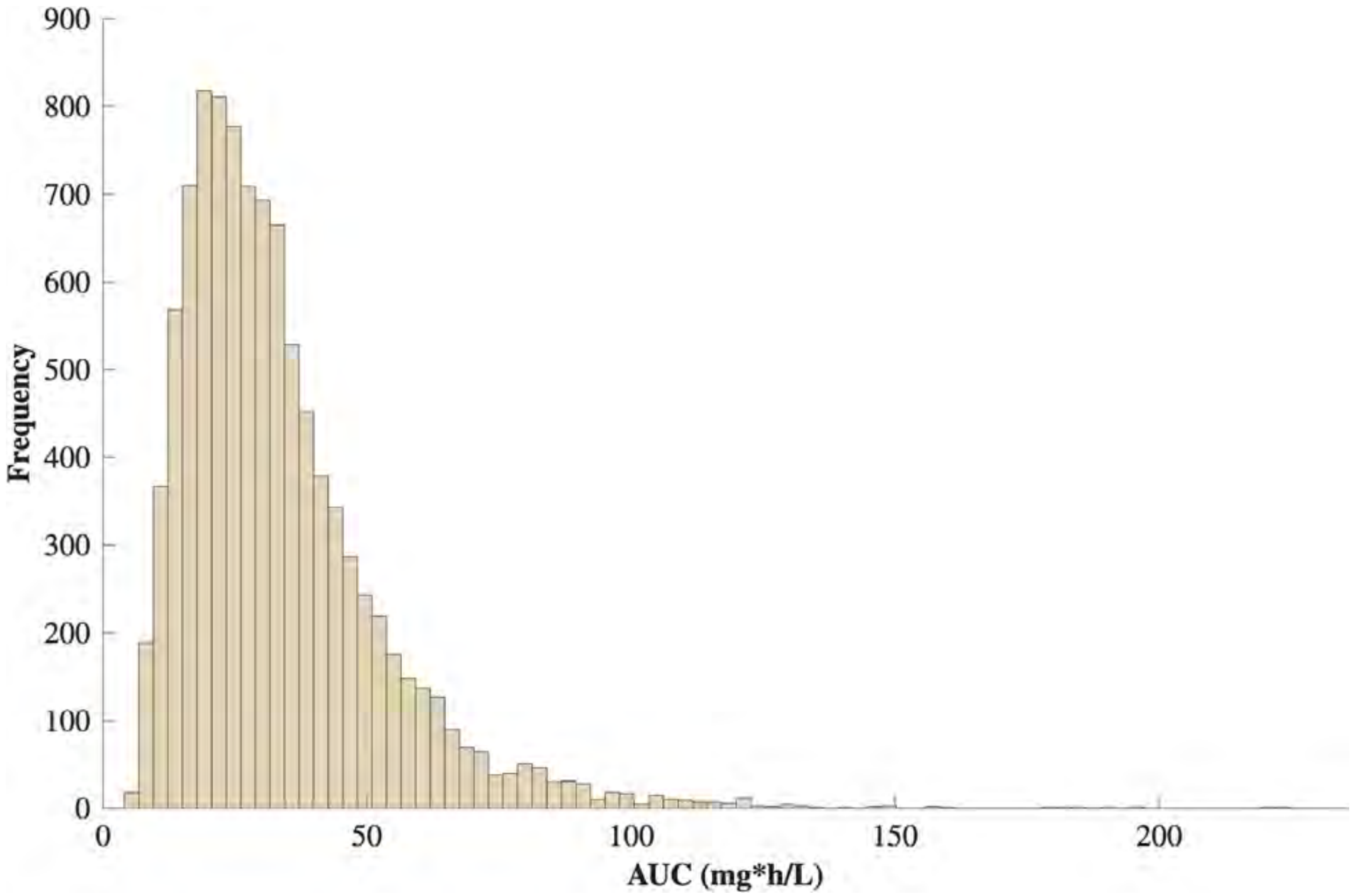

Figure S45: Steady-state AUC_{0-24} distributions across 10,000 simulated patient profiles for rifampin at 600 mg q24h. Distributions reflect interindividual variability drawn from the parameter distributions in Table S12.

M.5 Rifampin model concentration time-courses

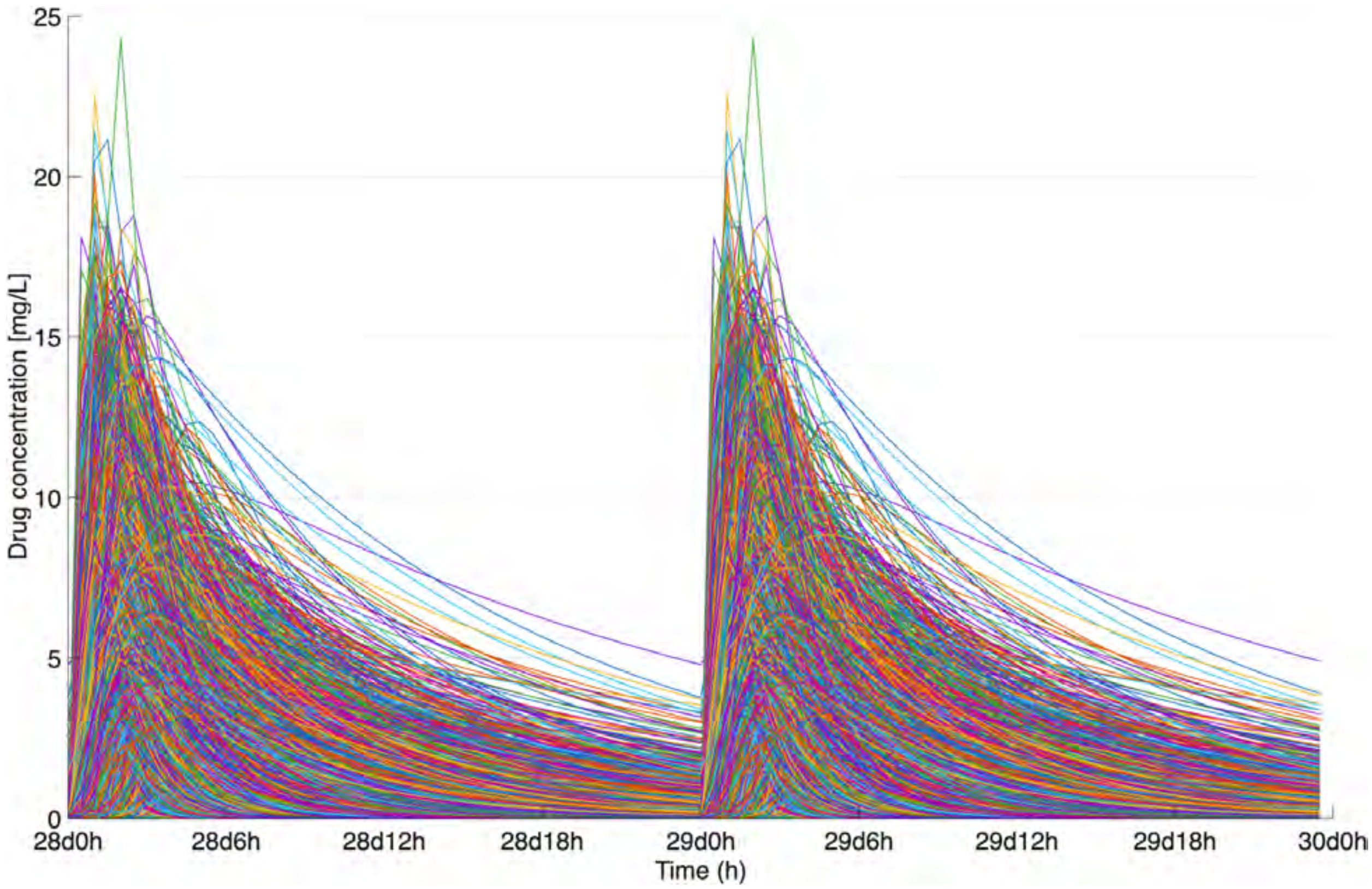

Figure S46: Simulated steady-state central compartment concentration-time profiles for rifampin across 10,000 virtual patients receiving 600 mg q24h.

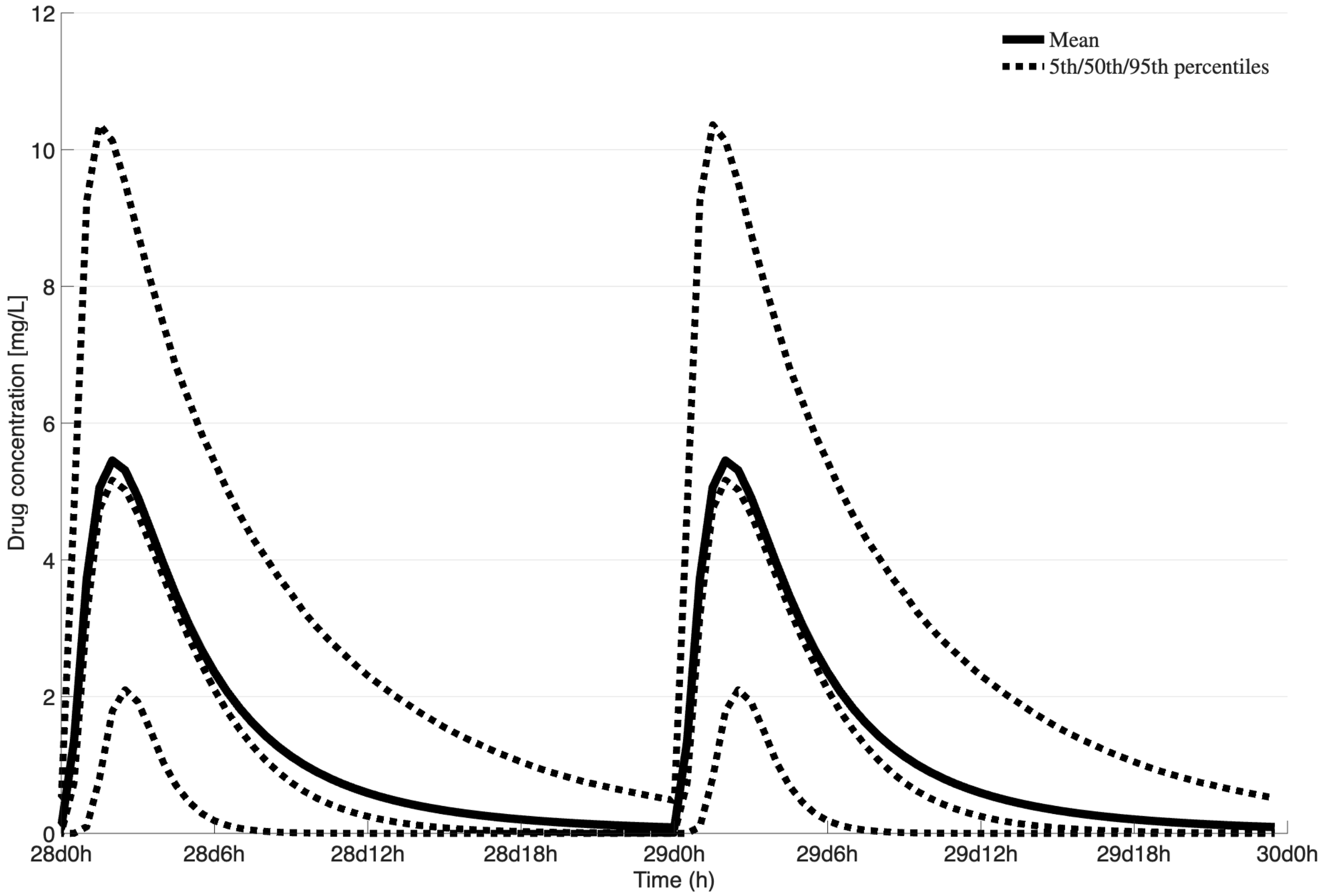

Figure S47: Simulated steady-state central compartment concentration-time profiles for rifampin showing the mean and 90% prediction interval across 10,000 virtual patients receiving 600 mg q24h.

N Tigecycline

N.1 Tigecycline model parameters

Table S13: Population pharmacokinetic parameters for the two-compartment tigecycline model. Values represent typical population means; IIV is expressed as percent coefficient of variation ($\%CV$).

Parameter	Mean	IIV % CV	Units
V_F,1	63.7	50.2	L
V_F,2	233	–	L
CL_F	14.8	46.6	L/h
Q_F	38.4	–	L/h

V_F,1 is the central compartment volume of distribution; V_F,2 is the peripheral compartment volume of distribution; CL_F is the clearance from the central compartment; Q_F is the intercompartmental clearance between central and peripheral compartments.

N.2 Tigecycline model equations

This model was recreated from the model proposed in [6]. The parameter values used in the patient simulations were calculated using Equation 4. The model structure consists of a central compartment (C) and a peripheral compartment (C_p), with drug administered as a loading dose followed by maintenance doses of 25 mg q12h as a 3-hour IV infusion as described by Equation 1.

$$\frac{dC}{dt} = \texttt{infusion\_rate} - \texttt{Q\_F} \cdot \frac{C}{\texttt{V\_F,1}} + \texttt{Q\_F} \cdot \frac{C_p}{\texttt{V\_F,2}} - \texttt{CL\_F} \cdot \frac{C}{\texttt{V\_F,1}} \tag{49}$$

$$\frac{dC_p}{dt} = \texttt{Q\_F} \cdot \frac{C}{\texttt{V\_F,1}} - \texttt{Q\_F} \cdot \frac{C_p}{\texttt{V\_F,2}} \tag{50}$$

N.3 Tigecycline model flowchart

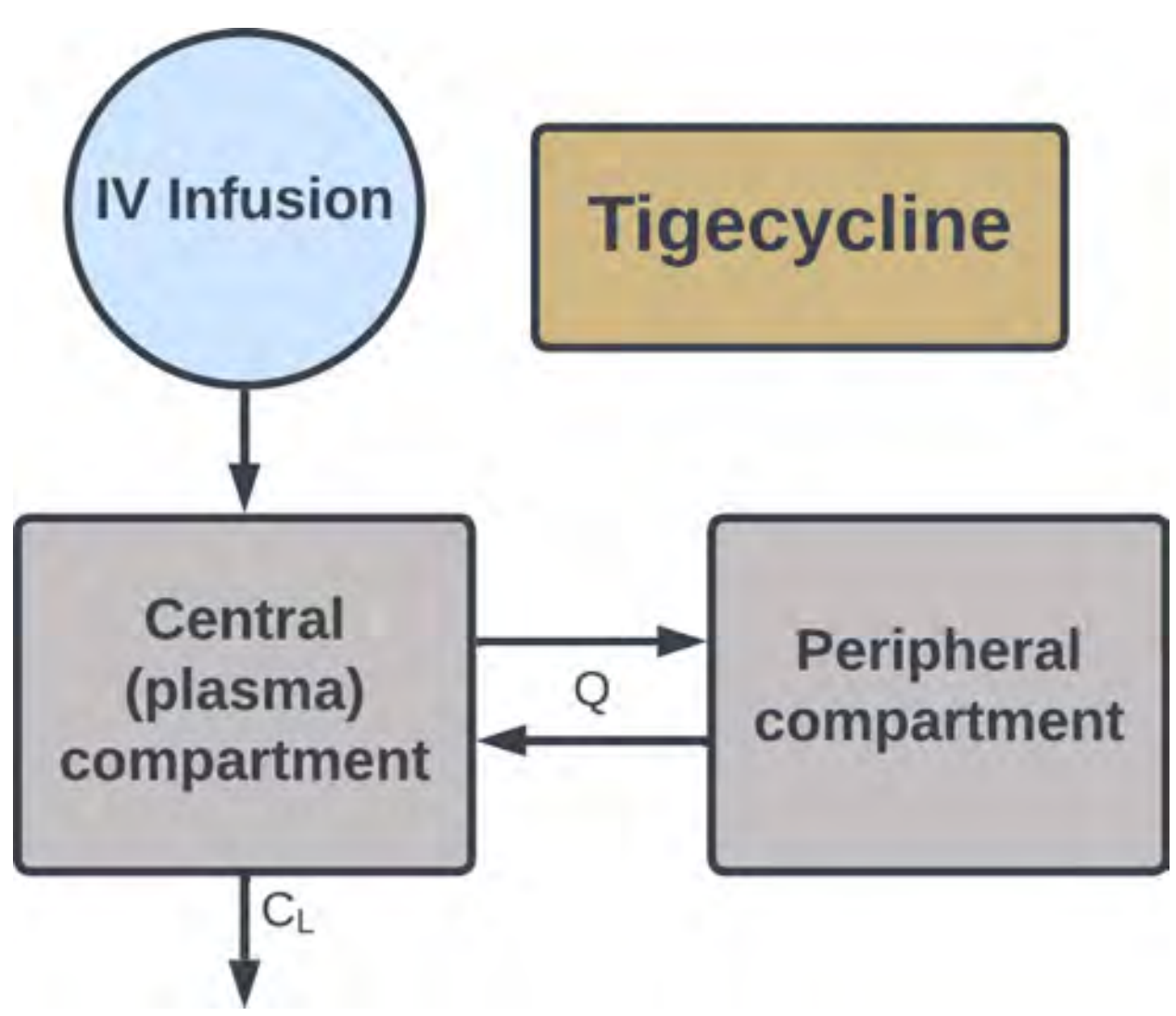

Figure S48: Schematic of the two-compartment population pharmacokinetic model for tigecycline, showing the central compartment (C) and peripheral compartment (C_p), with systemic clearance (CL_F), intercompartmental clearance (Q_F), and 3-hour IV infusion input with loading dose.

N.4 Tigecycline model AUC distributions

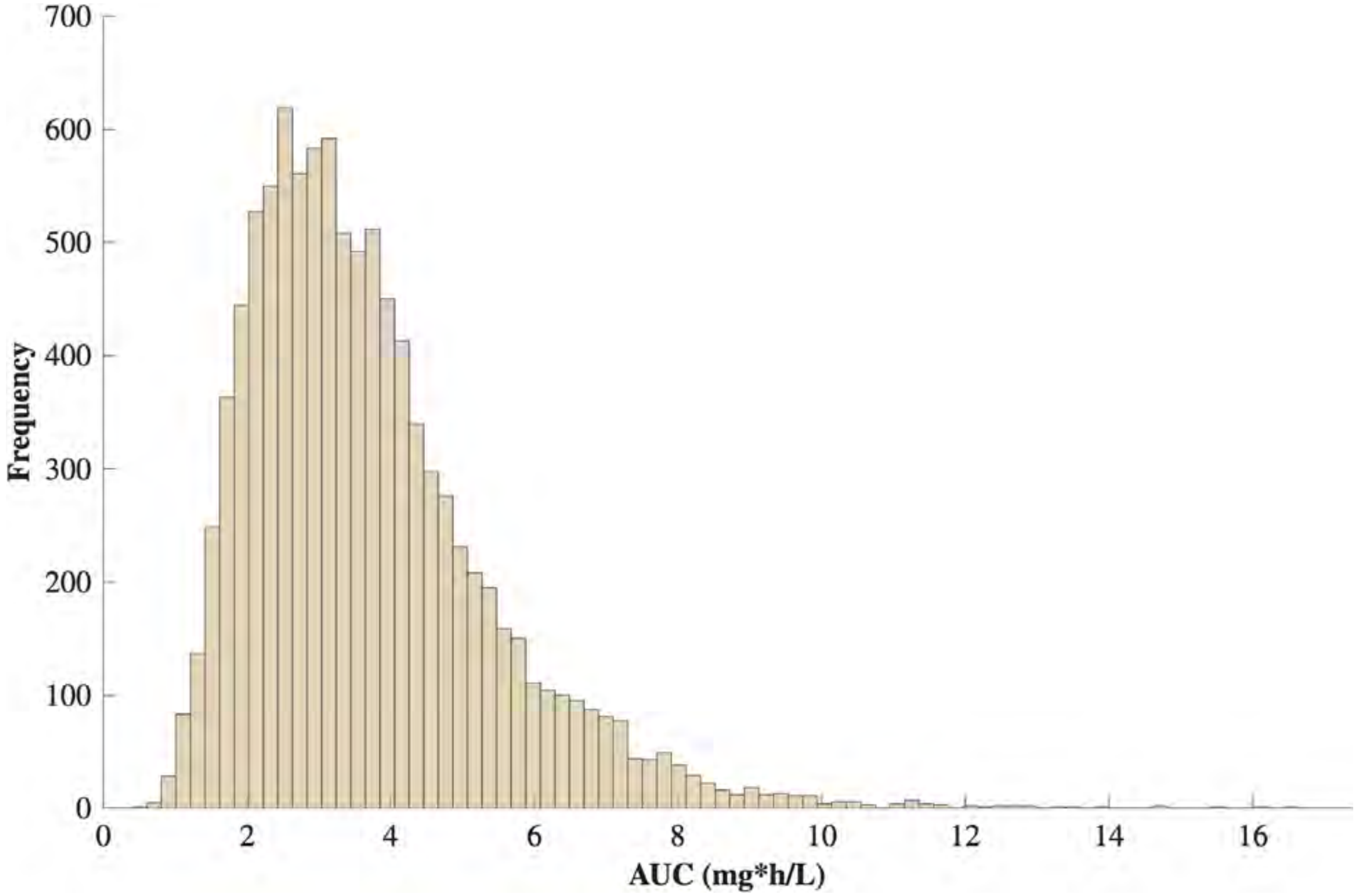

Figure S49: Steady-state $AUC_{0\text{–}24}$ distributions across 10,000 simulated patient profiles for tigecycline at 25 mg q12h administered as a 3-hour IV infusion. Distributions reflect interindividual variability drawn from the parameter distributions in Table S13.

N.5 Tigecycline model concentration time-courses

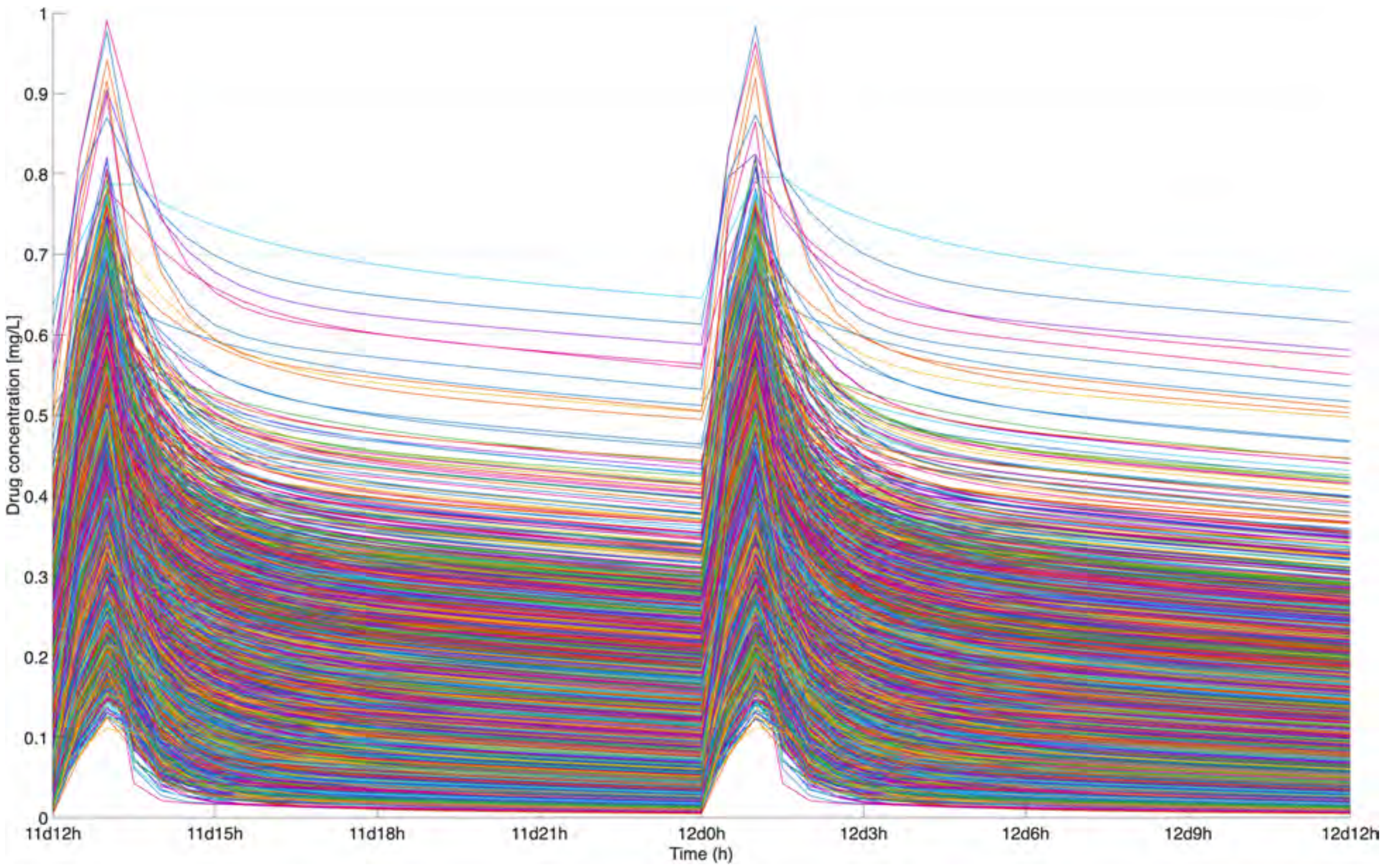

Figure S50: Simulated steady-state central compartment concentration-time profiles for tigecycline across 10,000 virtual patients receiving a loading dose followed by 25 mg q12h as a 3-hour IV infusion.

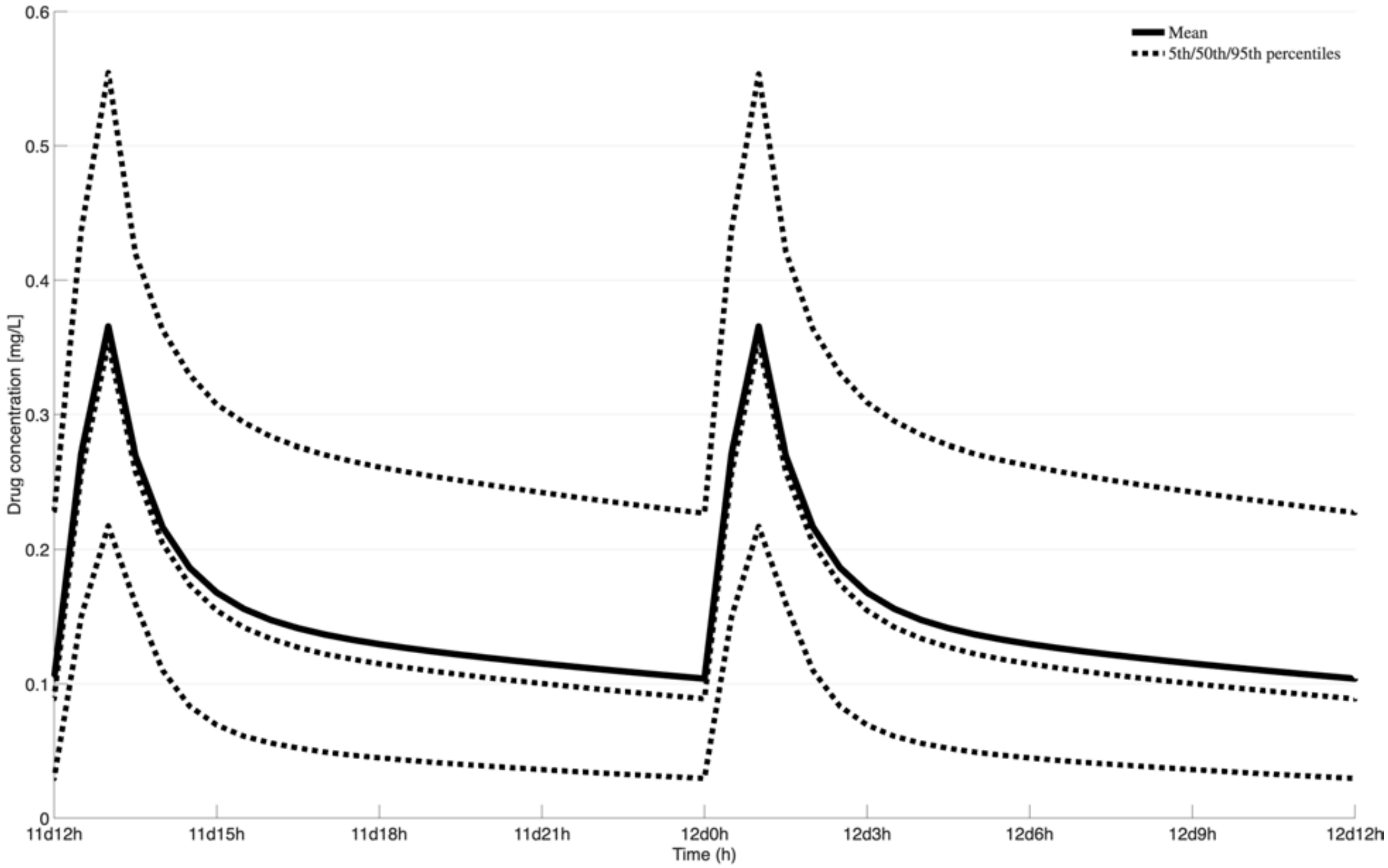

Figure S51: Simulated steady-state central compartment concentration-time profiles for tigecycline showing the mean and 90% prediction interval across 10,000 virtual patients receiving a loading dose followed by 25 mg q12h as a 3-hour IV infusion.

O Omadacycline

O.1 Omadacycline model parameters

The following table represents the omadacycline parameters for 300 mg q24h [48].

Table S14: Population pharmacokinetic parameters for the two-compartment omadacycline model with parallel absorption. Values represent typical population means; IIV is expressed as percent coefficient of variation ($\%CV$).

Parameter	Mean	IIV % CV	Units
V_F,1	103	17.0	L
V_F,2	244	30.1	L
CL_F,1	15.2	20.7	L/h
CL_F,2	34.2	23.8	L/h
k_a,1	0.342	57.2	1/h
k_a,2	4.58	72.1	1/h
P_1	0.786	10.8	–
T_lag	1.85	15.7	h

V_F,1 is the central compartment volume of distribution; V_F,2 is the peripheral compartment volume of distribution; CL_F,1 is the central compartment clearance; CL_F,2 is the intercompartmental clearance between central and peripheral compartments; k_a,1 and k_a,2 are the first-order absorption rate constants for the two parallel absorption compartments; P_1 is the fraction of dose entering the first absorption compartment; T_lag is the lag time applied to the second absorption compartment.

O.2 Omadacycline model equations

This model was recreated from the model proposed in [48]. The parameter values used in the patient simulations were calculated using Equation 4. The model structure was a 4-compartment model consisting of two parallel absorption compartments (q_1, q_2), a central compartment (C), and a peripheral compartment (P), with a lag time applied to the second absorption pathway.

$$\frac{d(q_1)}{dt} = -\texttt{k\_a,1} \cdot q_1 \tag{51}$$

$$\frac{d(q_2)}{dt} = -\texttt{k\_a,2} \cdot q_2, \quad t \geq \texttt{T\_lag} \tag{52}$$

$$\text{Rate In} = \texttt{k\_a,1} \cdot q_1 + 1_{(t \geq \texttt{T\_lag})} \cdot \texttt{k\_a,2} \cdot q_2 \tag{53}$$

$$\frac{dC}{dt} = \text{Rate In} - \texttt{CL\_F,1} \cdot \frac{C}{\texttt{V\_F,1}} - \texttt{CL\_F,2} \cdot \frac{C}{\texttt{V\_F,1}} + \texttt{CL\_F,2} \cdot \frac{P}{\texttt{V\_F,2}} \tag{54}$$

$$\frac{dP}{dt} = \texttt{CL\_F,2} \cdot \frac{C}{\texttt{V\_F,1}} - \texttt{CL\_F,2} \cdot \frac{P}{\texttt{V\_F,2}} \tag{55}$$

O.3 Omadacycline model flowchart

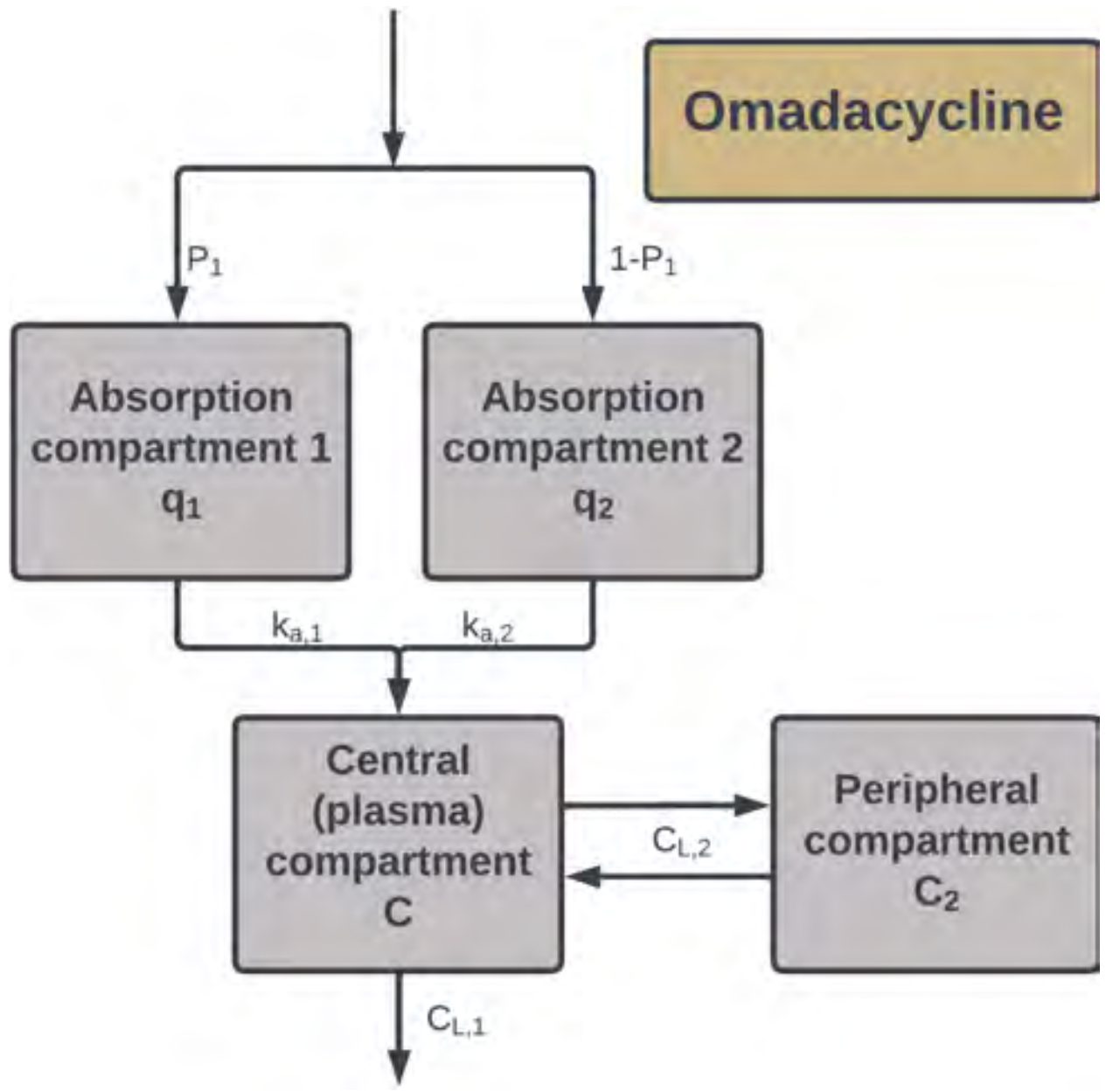

Figure S52: Schematic of the four-compartment population pharmacokinetic model for omadacycline, showing two parallel absorption compartments (q_1, q_2), central compartment (C), and peripheral compartment (P), with absorption rate constants (`k_a,1`, `k_a,2`), lag time (`T_lag`), systemic clearance (`CL_F,1`), and intercompartmental clearance (`CL_F,2`).

O.4 Omadacycline model AUC distributions

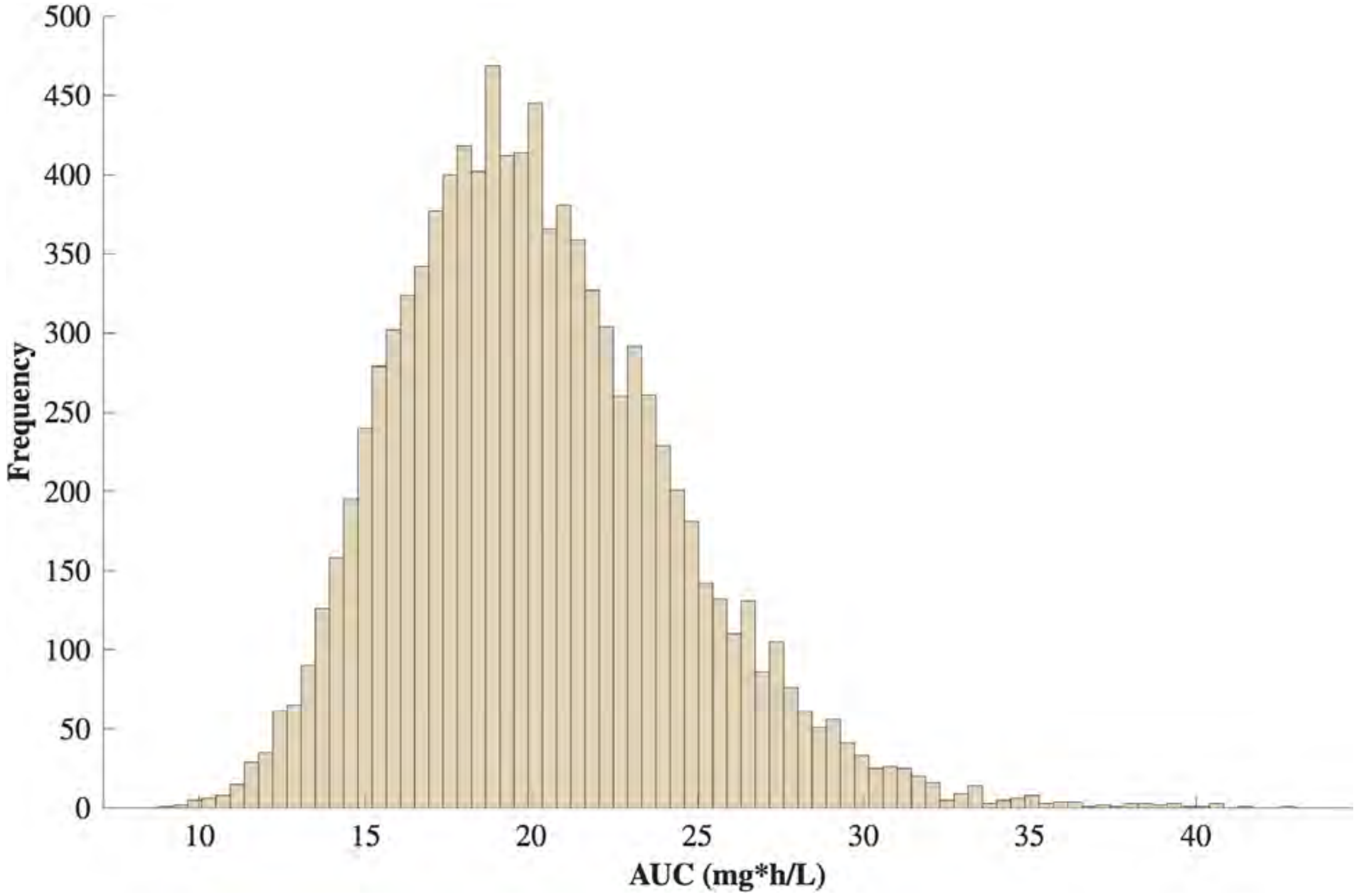

Figure S53: Steady-state AUC_{0-24} distributions across 10,000 simulated patient profiles for omadacycline at 300 mg q24h. Distributions reflect interindividual variability drawn from the parameter distributions in Table S14.

O.5 Omadacycline model concentration time-courses

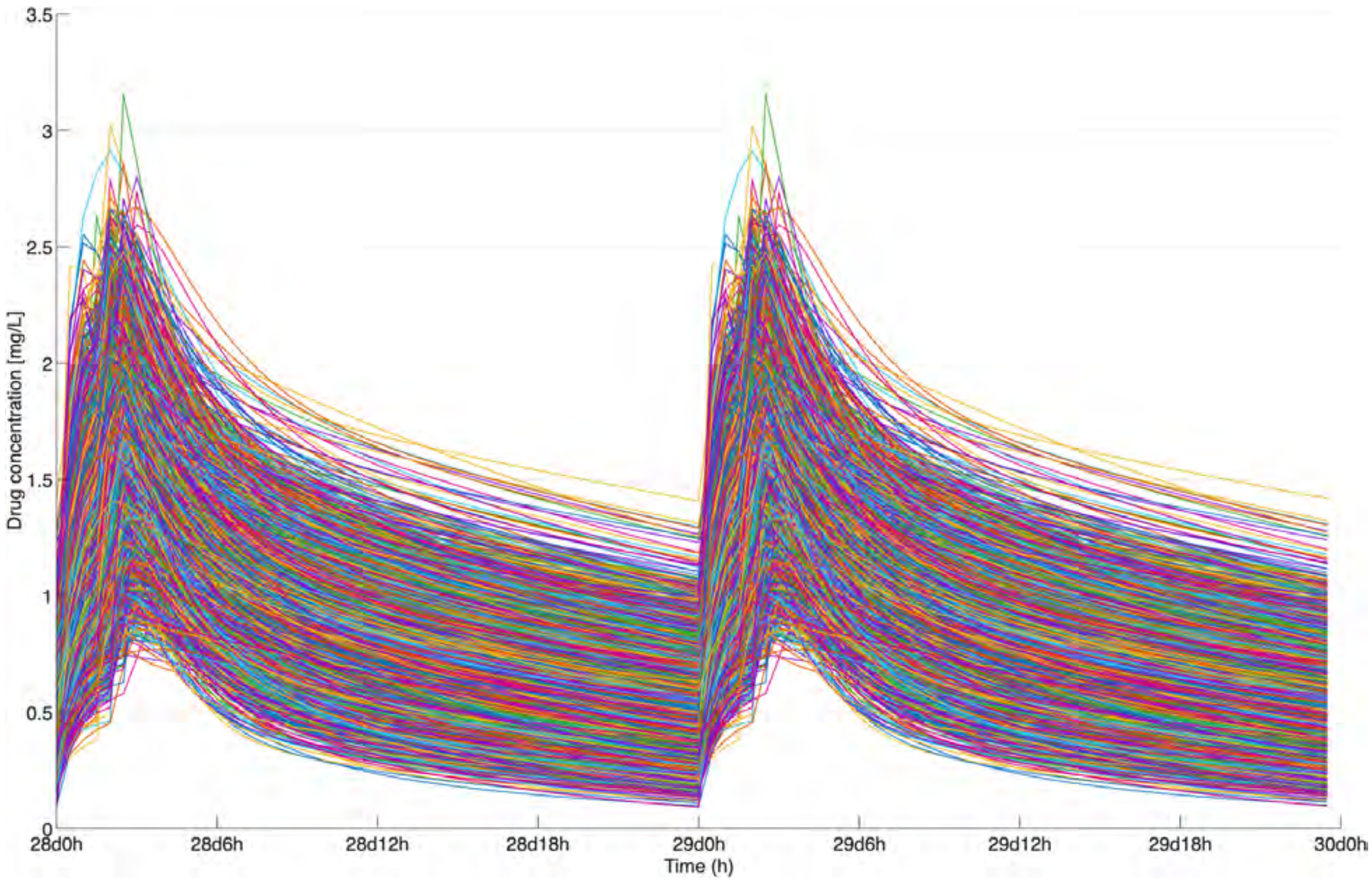

Figure S54: Simulated steady-state central compartment concentration-time profiles for omadacycline across 10,000 virtual patients receiving 300 mg q24h.

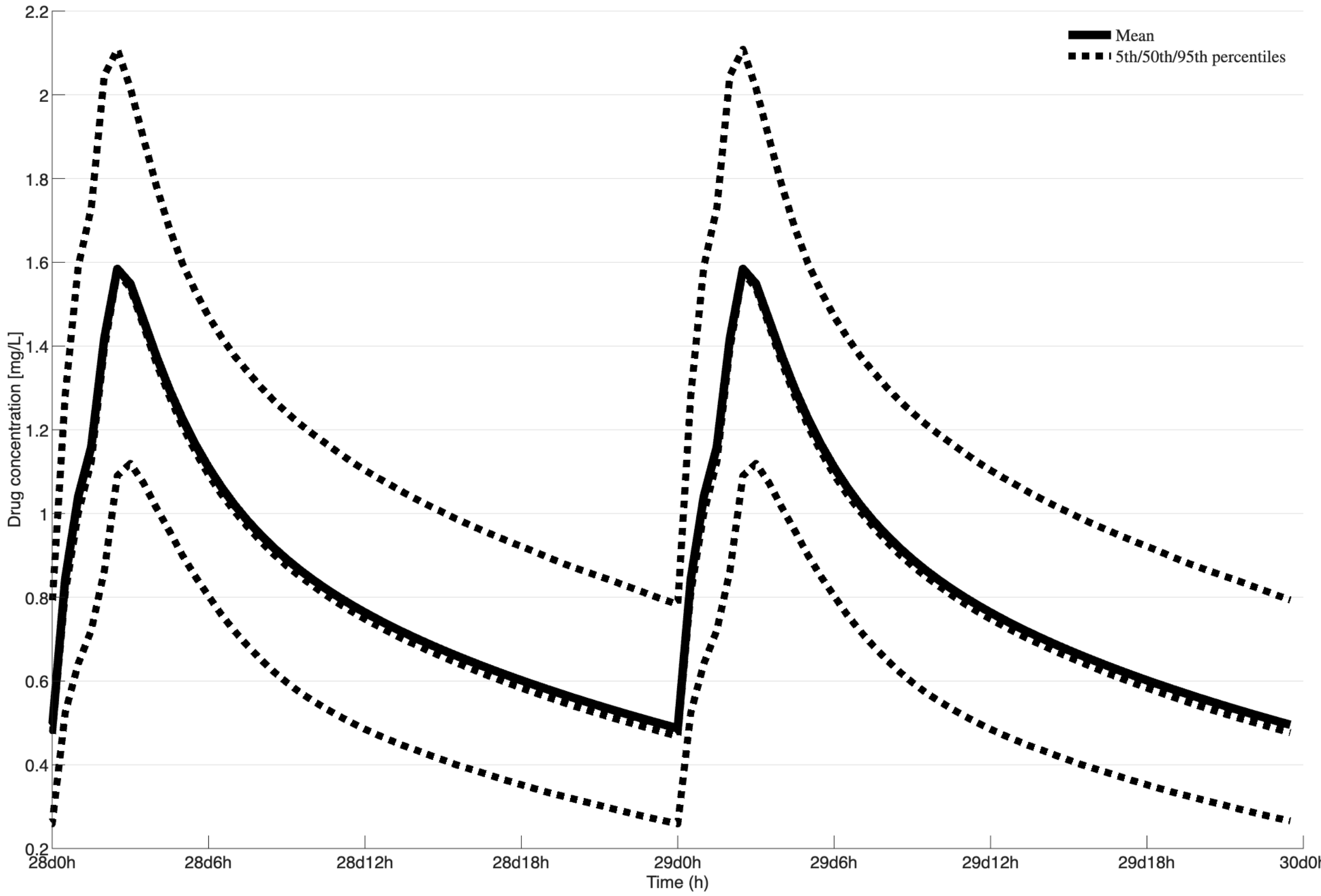

Figure S55: Simulated steady-state central compartment concentration-time profiles for omadacycline showing the mean and 90% prediction interval across 10,000 virtual patients receiving 300 mg q24h.

References

[1] Mahmoud Tareq Abdelwahab et al. “Clofazimine pharmacokinetics in patients with TB: dosing implications”. In: *Journal of Antimicrobial Chemotherapy* 75.11 (Aug. 2020), pp. 3269–3277. ISSN: 0305-7453. DOI: 10.1093/jac/dkaa310.

[2] Mahmoud Tareq Abdelwahab et al. “Clofazimine pharmacokinetics in patients with TB: dosing implications”. In: *Journal of Antimicrobial Chemotherapy* 75.11 (Nov. 2020), pp. 3269–3277. ISSN: 0305-7453. DOI: 10.1093/jac/dkaa310. eprint: https://academic.oup.com/jac/article-pdf/75/11/3269/33893035/dkaa310.pdf. URL: https://doi.org/10.1093/jac/dkaa310.

[3] Mahmoud Tareq Abdelwahab et al. “Linezolid population pharmacokinetics in South African adults with drug-resistant tuberculosis”. In: *Antimicrobial agents and chemotherapy* 65.12 (2021), pp. 10–1128.

[4] Khaled Abduljalil et al. “Modeling the Autoinhibition of Clarithromycin Metabolism during Repeated Oral Administration”. In: *Antimicrobial Agents and Chemotherapy* 53.7 (2009), pp. 2892–2901. DOI: 10.1128/aac.01193-08. eprint: https://journals.asm.org/doi/pdf/10.1128/aac.01193-08. URL: https://journals.asm.org/doi/abs/10.1128/aac.01193-08.

[5] Sujata M. Bhavnani et al. “Assessment of pharmacokinetics-pharmacodynamics to support omadacycline dosing regimens for the treatment of patients with acute bacterial skin and skin structure infections”. In: *Antimicrobial Agents and Chemotherapy* 68.9 (2024), e01281–23. DOI: 10.1128/aac.01281-23. eprint: https://journals.asm.org/doi/pdf/10.1128/aac.01281-23. URL: https://journals.asm.org/doi/abs/10.1128/aac.01281-23.

[6] Agnieszka Borsuk-De Moor et al. “Population pharmacokinetics of high-dose tigecycline in patients with sepsis or septic shock”. In: *Antimicrobial agents and chemotherapy* 62.4 (2018), pp. 10–1128.

[7] Catherine Boulanger et al. “Pharmacokinetic Evaluation of Rifabutin in Combination with Lopinavir-Ritonavir in Patients with HIV Infection and Active Tuberculosis”. In: *Clinical Infectious Diseases* 49.9 (Nov. 2009), pp. 1305–1311. ISSN: 1058-4838. DOI: 10.1086/606056. eprint: https://academic.oup.com/cid/article-pdf/49/9/1305/847795/49-9-1305.pdf. URL: https://doi.org/10.1086/606056.

[8] Olaf Burkhardt et al. “Single- and multiple-dose pharmacokinetics of linezolid and co-amoxiclav in healthy human volunteers”. In: *Journal of Antimicrobial Chemotherapy* 50.5 (Nov. 2002), pp. 707–712. ISSN: 0305-7453. DOI: 10.1093/jac/dkf163. eprint: https://academic.oup.com/jac/article-pdf/50/5/707/2265839/dkf163.pdf. URL: https://doi.org/10.1093/jac/dkf163.

[9] Wenqian Chen et al. “Imipenem population pharmacokinetics: therapeutic drug monitoring data collected in critically ill patients with or without extracorporeal membrane oxygenation”. In: *Antimicrobial agents and chemotherapy* 64.6 (2020), pp. 10–1128.

[10] M. T. Chirehwa et al. “Optimizing Moxifloxacin Dose in MDR-TB Participants with or without Efavirenz Coadministration Using Population Pharmacokinetic Modeling”. eng. In: *Antimicrobial agents and chemotherapy* 67.3 (2023), e0142622–. ISSN: 0066-4804.

[11] Christopher A. Czaja et al. “Cefoxitin Continuous Infusion for Lung Infection Caused by the Mycobacterium abscessus Group”. In: *Antimicrobial Agents and Chemotherapy* 58.6 (2014), pp. 3570–3571. DOI: 10.1128/aac.02763-14. eprint: https://journals.asm.org/doi/pdf/10.1128/aac.02763-14. URL: https://journals.asm.org/doi/abs/10.1128/aac.02763-14.

[12] Paolo Denti et al. “Pharmacokinetics of Isoniazid, Pyrazinamide, and Ethambutol in Newly Diagnosed Pulmonary TB Patients in Tanzania”. In: *PLOS ONE* 10 (Oct. 2015), pp. 1–19. DOI: 10.1371/journal.pone.0141002. URL: https://doi.org/10.1371/journal.pone.0141002.

[13] Andreas H. Diacon et al. “The Diarylquinoline TMC207 for Multidrug-Resistant Tuberculosis”. In: *New England Journal of Medicine* 360.23 (2009), pp. 2397–2405. DOI: 10.1056/NEJMoa0808427. eprint: https://www.nejm.org/doi/pdf/10.1056/NEJMoa0808427. URL: https://www.nejm.org/doi/full/10.1056/NEJMoa0808427.

[14] Haiyan Dong et al. "Clinical pharmacokinetic/pharmacodynamic profile of linezolid in severely ill Intensive Care Unit patients". eng. In: *International journal of antimicrobial agents* 38.4 (2011), pp. 296–300. ISSN: 0924-8579.

[15] R Garraffo et al. "Determination of optimal dosage regimen for amikacin in healthy volunteers by study of pharmacokinetics and bactericidal activity". In: *Antimicrobial agents and chemotherapy* 34.4 (1990), pp. 614–621.

[16] Stefanie Hennig et al. "Population pharmacokinetic drug–drug interaction pooled analysis of existing data for rifabutin and HIV PIs". In: *Journal of Antimicrobial Chemotherapy* 71.5 (2016), pp. 1330–1340.

[17] Stefanie Hennig et al. "Population pharmacokinetic drug–drug interaction pooled analysis of existing data for rifabutin and HIV PIs". In: *Journal of Antimicrobial Chemotherapy* 71.5 (May 2016), pp. 1330–1340. ISSN: 0305-7453. DOI: 10.1093/jac/dkv470. eprint: https://academic.oup.com/jac/article-pdf/71/5/1330/13762475/dkv470.pdf. URL: https://doi.org/10.1093/jac/dkv470.

[18] Jennifer A Hughes et al. "Pharmacokinetics and Safety of Clofazimine in Children With Rifampicin-Resistant Tuberculosis". In: *The Journal of Infectious Diseases* 231.5 (May 2025), e873–e881. ISSN: 0022-1899. DOI: 10.1093/infdis/jiaf057. eprint: https://academic.oup.com/jid/article-pdf/231/5/e873/61850888/jiaf057.pdf. URL: https://doi.org/10.1093/infdis/jiaf057.

[19] Tran Minh Hung et al. "Recommendation of pharmacokinetics/pharmacodynamics target of ethambutol to suppress tuberculosis resistance: A population pharmacokinetics study on a large prospective cohort". In: *International Journal of Antimicrobial Agents* 62.2 (2023), p. 106840. ISSN: 0924-8579. DOI: https://doi.org/10.1016/j.ijantimicag.2023.106840. URL: https://www.sciencedirect.com/science/article/pii/S092485792300119X.

[20] K. Ikawa et al. "Pharmacokinetic modelling of serum and bronchial concentrations for clarithromycin and telithromycin, and site-specific pharmacodynamic simulation for their dosages". In: *Journal of Clinical Pharmacy and Therapeutics* 39.4 (Mar. 2014), pp. 411–417. DOI: https://doi.org/10.1111/jcpt.12157.

[21] Jakko van Ingen et al. "The pharmacokinetics and pharmacodynamics of pulmonary Mycobacterium avium complex disease treatment". In: *American journal of respiratory and critical care medicine* 186.6 (2012), pp. 559–565.

[22] Sutep Jaruratanasirikul and Teeratad Sudsai. "Comparison of the pharmacodynamics of imipenem in patients with ventilator-associated pneumonia following administration by 2 or 0.5 h infusion". In: *Journal of Antimicrobial Chemotherapy* 63.3 (Mar. 2009), pp. 560–563. ISSN: 0305-7453. DOI: 10.1093/jac/dkn543. eprint: https://academic.oup.com/jac/article-pdf/63/3/560/2043484/dkn543.pdf. URL: https://doi.org/10.1093/jac/dkn543.

[23] Xuanyou Jin et al. "Population pharmacokinetic analysis of amikacin for optimal pharmacotherapy in Korean patients with nontuberculous mycobacterial pulmonary disease". In: *Antibiotics* 9.11 (2020), p. 784.

[24] Siv Jönsson et al. "Population Pharmacokinetics of Ethambutol in South African Tuberculosis Patients". In: *Antimicrobial Agents and Chemotherapy* 55.9 (2011), pp. 4230–4237. DOI: 10.1128/aac.00274-11.

[25] H Ko et al. "Pharmacokinetics of intravenously administered cefmetazole and cefoxitin and effects of probenecid on cefmetazole elimination". In: *Antimicrobial Agents and Chemotherapy* 33.3 (1989), pp. 356–361. DOI: 10.1128/aac.33.3.356. eprint: https://journals.asm.org/doi/pdf/10.1128/aac.33.3.356. URL: https://journals.asm.org/doi/abs/10.1128/aac.33.3.356.

[26] Ira M. Leviton and Maria Amodio-Groton. "Omadacycline Oral Dosing and Pharmacokinetics in Community-Acquired Bacterial Pneumonia and Acute Bacterial Skin and Skin Structure Infection". eng. In: *Clinical drug investigation* 42.3 (2022), pp. 193–197. ISSN: 1173-2563.

[27] Michael A. Lyons. "Pharmacodynamics and Bactericidal Activity of Bedaquiline in Pulmonary Tuberculosis". In: *Antimicrobial Agents and Chemotherapy* 66.2 (2022). DOI: 10.1128/aac.01636-21.

[28] Bryan McGee et al. “Population Pharmacokinetics of Linezolid in Adults with Pulmonary Tuberculosis”. In: *Antimicrobial Agents and Chemotherapy* 53.9 (2009), pp. 3981–3984. DOI: 10.1128/aac.01378-08. eprint: https://journals.asm.org/doi/pdf/10.1128/aac.01378-08. URL: https://journals.asm.org/doi/abs/10.1128/aac.01378-08.

[29] Sarah C. McLeay et al. “Population Pharmacokinetics of Bedaquiline (TMC207), a Novel Antituberculosis Drug”. In: *Antimicrobial Agents and Chemotherapy* 58.9 (2014), pp. 5315–5324. DOI: 10.1128/aac.01418-13. eprint: https://journals.asm.org/doi/pdf/10.1128/aac.01418-13. URL: https://journals.asm.org/doi/abs/10.1128/aac.01418-13.

[30] A. K. Meagher et al. “Exposure-Response Analyses of Tigecycline Efficacy in Patients with Complicated Skin and Skin-Structure Infections”. In: *Antimicrobial Agents and Chemotherapy* 51.6 (2007), pp. 1939–1945. DOI: 10.1128/aac.01084-06. eprint: https://journals.asm.org/doi/pdf/10.1128/aac.01084-06. URL: https://journals.asm.org/doi/abs/10.1128/aac.01084-06.

[31] PA Moise, MC Birmingham, and JJ Schentag. “Pharmacokinetics and metabolism of moxifloxacin.” In: *Drugs of today (Barcelona, Spain: 1998)* 36.4 (2000), pp. 229–244.

[32] Emmanuel Novy et al. “Population pharmacokinetics of prophylactic cefoxitin in elective bariatric surgery patients: a prospective monocentric study”. In: *Anaesthesia Critical Care & Pain Medicine* 43.3 (2024), p. 101376.

[33] Kristina Öbrink-Hansen et al. “Moxifloxacin Pharmacokinetic Profile and Efficacy Evaluation in Empiric Treatment of Community-Acquired Pneumonia”. In: *Antimicrobial Agents and Chemotherapy* 59.4 (2015), pp. 2398–2404. DOI: 10.1128/aac.04659-14. eprint: https://journals.asm.org/doi/pdf/10.1128/aac.04659-14. URL: https://journals.asm.org/doi/abs/10.1128/aac.04659-14.

[34] Charles A. Peloquin et al. “Pharmacokinetics of Ethambutol under Fasting Conditions, with Food, and with Antacids”. In: *Antimicrobial Agents and Chemotherapy* 43.3 (1999), pp. 568–572. DOI: 10.1128/aac.43.3.568. eprint: https://journals.asm.org/doi/pdf/10.1128/aac.43.3.568. URL: https://journals.asm.org/doi/abs/10.1128/aac.43.3.568.

[35] Charles A. Peloquin et al. “Population Pharmacokinetics of Levofloxacin, Gatifloxacin, and Moxifloxacin in Adults with Pulmonary Tuberculosis”. eng. In: *Antimicrobial Agents and Chemotherapy* 52.3 (2008), pp. 852–857. ISSN: 0066-4804.

[36] Keith A. Rodvold. “Clinical Pharmacokinetics of Clarithromycin”. eng. In: *Clinical pharmacokinetics* 37.5 (1999), pp. 385–398. ISSN: 0312-5963.

[37] Madeline Sanders et al. “Pharmacokinetics of Omadacycline in Adults with Cystic Fibrosis: Pharmacokinetic of Omadacycline in Cystic Fibrosis”. eng. In: *Clinical pharmacokinetics* 63.12 (2024), pp. 1701–1709. ISSN: 0312-5963.

[38] Mohammad H. Al-Shaer et al. “Fluoroquinolones in Drug-Resistant Tuberculosis: Culture Conversion and Pharmacokinetic/Pharmacodynamic Target Attainment To Guide Dose Selection”. In: *Antimicrobial Agents and Chemotherapy* 63.7 (2019). DOI: 10.1128/aac.00279-19.

[39] Hinke Siebinga, Fiona Robb, and Alison H Thomson. “Population pharmacokinetic evaluation and optimization of amikacin dosage regimens for the management of mycobacterial infections”. In: *Journal of Antimicrobial Chemotherapy* 75.10 (2020), pp. 2933–2940.

[40] S A Signs et al. “Pharmacokinetics of imipenem in serum and skin window fluid in healthy adults after intramuscular or intravenous administration”. In: *Antimicrobial Agents and Chemotherapy* 36.7 (1992), pp. 1400–1403. DOI: 10.1128/aac.36.7.1400. eprint: https://journals.asm.org/doi/pdf/10.1128/aac.36.7.1400. URL: https://journals.asm.org/doi/abs/10.1128/aac.36.7.1400.

[41] M H Skinner et al. “Pharmacokinetics of rifabutin”. In: *Antimicrobial Agents and Chemotherapy* 33.8 (1989), pp. 1237–1241. DOI: 10.1128/aac.33.8.1237. eprint: https://journals.asm.org/doi/pdf/10.1128/aac.33.8.1237. URL: https://journals.asm.org/doi/abs/10.1128/aac.33.8.1237.

[42] Dennis J Stalker and Gail L Jungbluth. “Clinical Pharmacokinetics of Linezolid, a Novel Oxazolidinone Antibacterial”. eng. In: *Clinical pharmacokinetics* 42.13 (2003), pp. 1129–1140. ISSN: 0312-5963.

[43] K E Stott et al. "Pharmacokinetics of rifampicin in adult TB patients and healthy volunteers: a systematic review and meta-analysis". In: *Journal of Antimicrobial Chemotherapy* 73.9 (Sept. 2018), pp. 2305–2313. ISSN: 0305-7453. DOI: 10.1093/jac/dky152. eprint: https://academic.oup.com/jac/article-pdf/73/9/2305/25523172/dky152.pdf. URL: https://doi.org/10.1093/jac/dky152.

[44] Michel Tod et al. "Population pharmacokinetic study of amikacin administered once or twice daily to febrile, severely neutropenic adults". In: *Antimicrobial agents and chemotherapy* 42.4 (1998), pp. 849–856.

[45] Xiaohong Wang et al. "A Single- and Multiple-Dose Study To Characterize the Pharmacokinetics, Safety, and Tolerability of Imipenem and Relebactam in Healthy Chinese Participants". In: *Antimicrobial Agents and Chemotherapy* 65.3 (2021), 10.1128/aac.01391–20. DOI: 10.1128/aac.01391-20. eprint: https://journals.asm.org/doi/pdf/10.1128/aac.01391-20. URL: https://journals.asm.org/doi/abs/10.1128/aac.01391-20.

[46] Justin J. Wilkins et al. "Population Pharmacokinetics of Rifampin in Pulmonary Tuberculosis Patients, Including a Semimechanistic Model To Describe Variable Absorption". In: *Antimicrobial Agents and Chemotherapy* 52.6 (2008), pp. 2138–2148. DOI: 10.1128/aac.00461-07.

[47] Noriaki Yamashita et al. "Tigecycline pharmacokinetics, tolerability, safety, and effect on intestinal microflora in healthy Japanese male subjects". In: *The Journal of Clinical Pharmacology* 54.5 (2014), pp. 513–519. DOI: https://doi.org/10.1002/jcph.236. eprint: https://accp1.onlinelibrary.wiley.com/doi/pdf/10.1002/jcph.236. URL: https://accp1.onlinelibrary.wiley.com/doi/abs/10.1002/jcph.236.

[48] Haijing Yang et al. "Pharmacokinetics, Safety and Pharmacokinetics/Pharmacodynamics Analysis of Omadacycline in Chinese Healthy Subjects". In: *Frontiers in Pharmacology* Volume 13 (2022). ISSN: 1663-9812. DOI: 10.3389/fphar.2022.869237.

[49] Hwi-Yeol Yun et al. "Model-Based Efficacy and Toxicity Comparisons of Moxifloxacin for Multidrug-Resistant Tuberculosis". In: *Open Forum Infectious Diseases* 9.3 (Mar. 2022), ofab660. ISSN: 2328-8957. DOI: 10.1093/ofid/ofab660. eprint: https://academic.oup.com/ofid/article-pdf/9/3/ofab660/42405541/ofab660.pdf. URL: https://doi.org/10.1093/ofid/ofab660.
